# TAMPA: interpretable analysis and visualization of metagenomics-based taxon abundance profiles

**DOI:** 10.1093/gigascience/giad008

**Published:** 2023-02-28

**Authors:** Varuni Sarwal, Jaqueline Brito, Serghei Mangul, David Koslicki

**Affiliations:** Department of Computer Science, University of California–Los Angeles, Los Angeles, CA 90095, USA; Titus Family Department of Clinical Pharmacy, USC Alfred E. Mann School of Pharmacy and Pharmaceutical Sciences,University of Southern California, Los Angeles, CA 90089, USA; Titus Family Department of Clinical Pharmacy, USC Alfred E. Mann School of Pharmacy and Pharmaceutical Sciences,University of Southern California, Los Angeles, CA 90089, USA; Department of Quantitative and Computational Biology, USC Dornsife College of Letters, Arts and Sciences, University of Southern California, Los Angeles, CA 90089, USA; Department of Computer Science and Engineering, The Pennsylvania State University, University Park, PA 16802, USA; Department of Biology, The Pennsylvania State University, University Park, PA 16802, USA; Huck Institutes of the Life Sciences, The Pennsylvania State University, University Park, PA 16802, USA

**Keywords:** Computational Metagenomics, Visualization, Interpretability

## Abstract

**Background:**

Metagenomic taxonomic profiling aims to predict the identity and relative abundance of taxa in a given whole-genome sequencing metagenomic sample. A recent surge in computational methods that aim to accurately estimate taxonomic profiles, called taxonomic profilers, has motivated community-driven efforts to create standardized benchmarking datasets and platforms, standardized taxonomic profile formats, and a benchmarking platform to assess tool performance. While this standardization is essential, there is currently a lack of tools to visualize the standardized output of the many existing taxonomic profilers. Thus, benchmarking studies rely on a single-value metrics to compare performance of tools and compare to benchmarking datasets. This is one of the major problems in analyzing metagenomic profiling data, since single metrics, such as the F1 score, fail to capture the biological differences between the datasets.

**Findings:**

Here we report the development of TAMPA (Taxonomic metagenome profiling evaluation), a robust and easy-to-use method that allows scientists to easily interpret and interact with taxonomic profiles produced by the many different taxonomic profiler methods beyond the standard metrics used by the scientific community. We demonstrate the unique ability of TAMPA to generate a novel biological hypothesis by highlighting the taxonomic differences between samples otherwise missed by commonly utilized metrics.

**Conclusion:**

In this study, we show that TAMPA can help visualize the output of taxonomic profilers, enabling biologists to effectively choose the most appropriate profiling method to use on their metagenomics data. TAMPA is available on GitHub, Bioconda, and Galaxy Toolshed at https://github.com/dkoslicki/TAMPA and is released under the MIT license.

## Introduction

Microorganisms live in complex communities and play a vital role in human and environmental health. Studying these communities is important to understand how microbes interact with each other, their host, and the environment. Metagenomics has become an essential tool to study microbiomes due to improvements in technology and bioinformatic algorithms. One of the first steps in investigating microbial community dynamics is to estimate the abundance of different species in the community; this process is called taxonomic profiling. Taxonomic metagenome profiling aims to predict the identity and relative abundances of taxa in a given whole-genome sequencing (WGS) metagenomic sample. A recent surge in computational methods that aim to accomplish this, called taxonomic profilers, has motivated community-driven efforts to create standardized benchmarking datasets [1–3], standardized taxonomic profile formats [4], and a benchmarking platform to assess tool performance on simulated data [5]. While this standardization is essential, there is currently a lack of tools to visualize the standardized output of the many existing taxonomic profilers, and benchmarking studies rely on a single-value metrics to compare performance of tools and compare to benchmarking datasets. Indeed, the only 2 such WGS taxonomic profiling visualization and analysis tools that do exist are either integrated into a single taxonomic profiling method [6] or lack the flexibility and interpretability for the analysis and visualization of multiple taxonomic profiles [7]. Neither of these methods is designed for or compatible with the community-driven output formats previously mentioned.

Despite the availability of flexible and interactive visualization tools in the area of amplicon microbial analysis (such as 16S ribosomal RNA studies), similar methods are yet to be developed for WGS metagenomics. For example, metacoder [8] is a tool that allows for visualizing, analyzing, and manipulating amplicon microbial data. However, metacoder is not designed for WGS metagenomic analyses and cannot be used for analysis and visualizing metagenomic taxonomic profiles due to amplicon analyses relying on Operational Taxonomic Units, a concept that is not relevant to metagenomic studies. Similarly, the recently published preprint for the software package EMPress [9] is an interactive phylogenetic tree viewer not explicitly intended for the visualization of WGS taxonomic profiles.

Additionally, lack of tools that provide an interpretable visualization of multiple taxonomic profiles limits the ability of the biomedical community to select a tool. As such, when WGS metagenomic data are generated and a scientist wishes to determine which of the dozens [10–23] of taxonomic profilers to use, they typically rely on benchmark studies [1, 24, 25]. These benchmark studies often use simulated data that do not accurately reflect their samples of interest. Alternatively, they can run their own simulation and benchmarking study tailor to their use case, but this requires significant time investment [2]. Scientists often resort to simply picking a familiar tool regardless of its performance characteristics. Given the substantial variability in the performance of taxonomic profiling tools [1, 24, 25], this may result in misinterpretation of their data and can potentially lead to unfortunate situations where utilizing a single low-accuracy taxonomic profiling tool can lead to an interpretation of data [26] (i.e., presence of bubonic plague in the New York subway system) that is later to be found to be inaccurate [27].

To empower biomedical researchers with a robust and easy-to-use metagenomic taxonomic profile analysis and visualization platform, we have developed a software package TAMPA (Taxonomic metagenome profiling evaluation). Our platform assists scientists in contextualizing, assessing, and extracting insight from taxonomic profiles produced by multiple taxonomic profilers when applied to either real or simulated data. TAMPA is designed to allow users to effectively analyze 1 or more taxonomic profiles produced by any of the numerous taxonomic profiling methods. Additionally, TAMPA can operate on the widely utilized and community-developed BIOM [29] and CAMI [1] profiling formats. We demonstrate the utility of TAMPA by showing how it illuminates the important biological differences between samples and conditions otherwise missed by commonly utilized statistical metrics. When gold-standard taxonomic profiles are available, we show how TAMPA can augment existing benchmarking platforms such as OPAL by being incorporated within the tool and providing an interpretable visualization of the profiles [5]. Additionally, we show that TAMPA can enable biologists to choose an appropriate profiling method to use on their real data when a ground truth taxonomic profile is not available, since TAMPA allows users to quickly ascertain similarities or differences in predictions made by multiple taxonomic tools.

## Results

TAMPA is a computational tool that allows the user to effectively visualize 1 or more taxonomic profiles produced by taxonomic profiling methods. TAMPA contextualizes, assesses, and extracts insight from multiple taxonomic profiler results. Here, we demonstrate 3 major ways in which TAMPA provides a novel way to visualize the outputs of existing profilers and visualization platforms.

### TAMPA enables effective comparison of the outputs of multiple profilers

The Critical Assessment of Metagenome Interpretation (CAMI) [1–3] provides the most comprehensive and in-depth evaluation of metagenomic profiling, binning, and assembly methods to date. In the profiling competition, many of the most well-known profiling methods were evaluated on a variety of simulated datasets that modeled real-life challenges, such as various community diversities and confounding sequences from high-abundance plasmids and novel viral strains. To demonstrate the ability of TAMPA to provide an interpretable analysis and visualization of metagenomics-based taxon abundance profiles, we apply it to the results of 3 profiles from the publicly available CAMI dataset [1–3]: MetaPhyler [14], mOTU [15], and Taxy-Pro [30].

**Figure 1:**
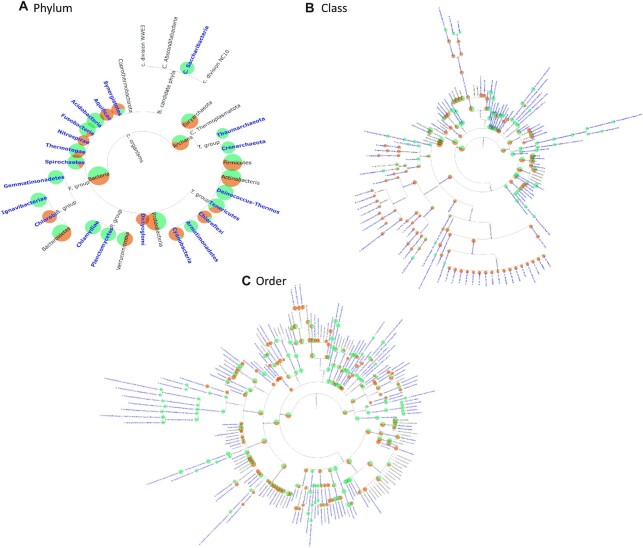
Visualization of the taxonomic profiles of tools with identical UniFrac scores of 4, Taxy-Pro (green) versus MetaPhyler (orange), using TAMPA on the CAMI dataset at the phylum, class, and order levels. The size of the discs represents the total amount of relative abundance at the corresponding clade in the output profiles. If the tool predictions agree, a disc is colored half orange and half green. The proportion of green to orange changes with respect to the disagreement in the prediction of that clade's relative abundance between the 2 tools is being compared. Highlighted blue text represents clades where the difference between the relative abundances of the predictions exceeds 30%.

TAMPA has 2 major modes for comparing output profiles. First, TAMPA can be used to compare the outputs of multiple profilers and reveal insight even when traditional metrics report no differences. TAMPA does this by computing the percentage relative abundance per taxa and identifying which specific clades contributed to metric values, thus revealing biological differences that could otherwise be overlooked when looking only at single-valued metrics. We choose 2 profilers with an identical UniFrac score on a particular sample [28], Taxy-Pro and Metaphyler, and demonstrate the specific differences in their predictions of taxonomic profiles using TAMPA on the phylum level (Fig. 1), as well as other taxonomic levels (Supplementary Figs. S1–S5). TAMPA can support up to 3 input profilers at once, illuminating differences in their relative abundances (Supplementary Fig. S17).

**Figure 2:**
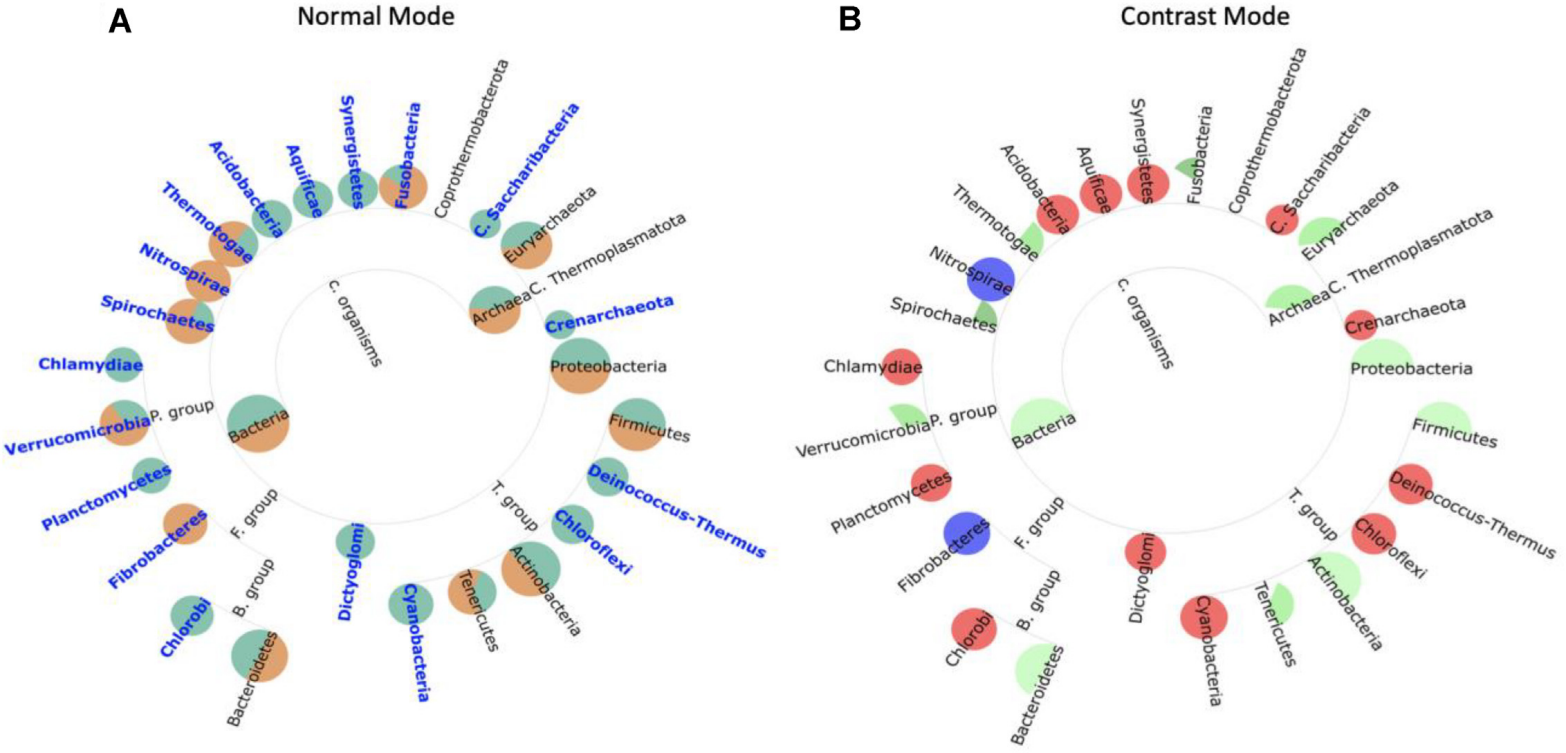
(A) Visualization of the taxonomic profile of a top-performing CAMI tool in terms of the L1 norm, MetaPhyler (green) versus the ground truth (orange), using TAMPA on the CAMI dataset at the phylum level. (B) Visualization of the taxonomic profile of a top-performing CAMI tool in terms of the L1 norm, MetaPhyler versus the ground truth, using TAMPA on the CAMI dataset at the phylum level using the contrast mode. False positives are denoted in red, false negatives in blue, and a gradient of white to green when the taxa expected from the ground truth are measured: in white if the relative abundance is the same and in green if the relative abundance is different between expected and measured, with the color intensity proportional to the error.

**Figure 3:**
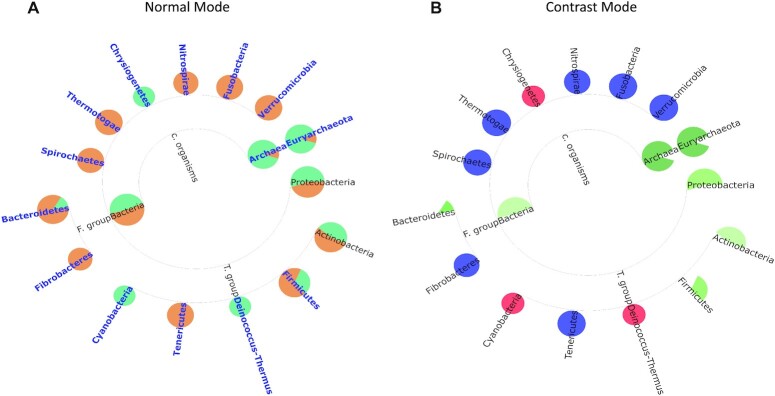
(A) Visualization of the taxonomic profile of the lowest-performing tool in terms of the L1 norm, mOTU (green) versus the ground truth (orange), using TAMPA on the CAMI dataset at the phylum level. (B) Visualization of the taxonomic profile of the lowest-performing tool in terms of the L1 norm, mOTU versus the ground truth, using TAMPA on the CAMI dataset at the phylum level using the contrast mode.

Second, even when tool performance is distinguishable by traditional numerical metrics, TAMPA can be used to quickly ascertain how tool predictions differ from the ground truth profile. For example, we chose both the top-performing (Fig. 2) and bottom-performing (Fig. 3) tools in terms of the L1 norm, according to the CAMI challenge—MetaPhyler and mOTU—and demonstrate that TAMPA can illuminate important biological differences between the 2 tools and the ground truth at the phylum level (Figs. 2 and 3), as well as at all other taxonomic ranks (Supplementary Figs. S6–S15). To better visualize the differences between the tool and the ground truth, we have created a special “contrast mode” in TAMPA. In the contrast mode, the false-positive taxa are represented as red circles, the false-negative taxa as blue circles, true positives as white, and the remaining taxa in a gradient of white to green, with the color intensity proportional to the relative error. This option is especially helpful when there are large trees, to identify problematic subregions (Supplementary Figs. S8–S10, S13–S15).

### TAMPA augments existing benchmarking platforms

Third, TAMPA can be used to augment existing benchmarking platforms. We have integrated TAMPA into the taxonomic profiling benchmarking platform OPAL [5] in order to provide biological insight when scientists and tool developers aim to benchmark and compare taxonomic profilers (Supplementary Fig. S16). OPAL is a popular web-based tool used to compute commonly used performance metrics for profiler outputs. While OPAL provides global metrics and visualizations, it is unable to provide specific information on the taxonomic differences in the profiles. Additionally, scientists can encounter difficulty when interpreting statistical measures of differences between the estimated taxonomic frequencies and the ground truth, as well as when comparing differences between tools. With the inclusion of TAMPA in OPAL, users can now quickly ascertain the performance of the tools being analyzed at a level of resolution not possible before. For example, by utilizing the figures returned by TAMPA, a user can quantify tool performance on a particular taxonomic clade of interest. Based on our results (e.g., Supplementary Fig. S1), we show that TAMPA can highlight important taxonomic differences easily missed by statistical metrics, thus enabling biologists to choose the most appropriate profiling method to use on their data.

### Marine metagenome prediction: A concept challenge

**Figure 4:**
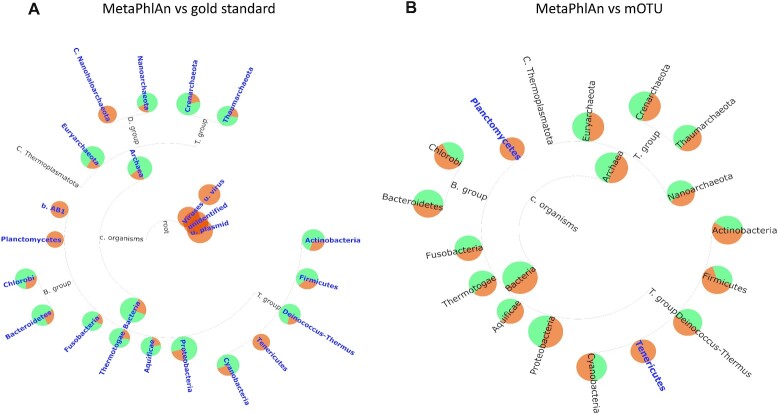
(A) Visualization of the taxonomic profile of the top-performing tool in term**s** of F1 score, MetaPhlAn (green) versus the ground truth (orange), using TAMPA on the CAMI marine dataset at the phylum level. (B) Visualization of the taxonomic profile of the 2 top-performing tools in terms of F1 score, MetaPhlAn (green) and mOTUs (orange), on the marine dataset at the phylum level.

Microbial communities are key drivers of marine biogeochemistry, and this will improve our understanding of the distribution of organisms in the oceans as well as the selective forces that structure community composition and distribution across space and time. We applied the TAMPA on the results of the 2 best-performing taxonomic profilers, MetaPhlAn 2.9.22 and mOTUs 2.5.1, on the marine dataset published in the “Critical Assessment of Metagenome Interpretation: The Second Round of Challenges” and demonstrate a case where TAMPA can provide different taxonomic interpretations of a microbial community that are biologically relevant. On comparing the output of MetaPhlAn with the gold standard, we use TAMPA to demonstrate that while MetaPhlAn was one of the top-performing profilers, it was not able to detect several clades, including Planctomycetes (Fig. 4A). Additionally, while MetaPhlAn and mOTUs were both the top-performing profilers in terms of single-valued metrics such as F1 score and L1 norm error, there were several critical differences between their outputs, highlighted by TAMPA at the phylum level (Fig. 4B). For example, MetaPhlAn failed to detect both the Tenericutes group and Planctomycetes. There was also a considerable difference in the percentage abundance of other groups, such as Firmicutes.

## Discussion

Metagenomics has emerged as a technology of choice for analyzing microbial communities, with thousands of WGS metagenomic samples being produced annually [31]. Taxonomic profiling is an important first step in analyzing metagenomic data since taxonomic profiles represent the taxonomic identities and relative abundances of microbial community members from metagenome samples. Comparing these taxonomic profiles with each other, as well as with the gold standard, is a nontrivial task, and there are no existing tools that provide a rigorous and intuitive analysis. Hence, TAMPA will be of broad interest to all scientists engaged in such research, thus allowing them to quickly contextualize, assess, and extract insight from taxonomic profiles instead of relying primarily on statistical summaries or manual manipulation. Indeed, TAMPA was effectively applied in the second round of the CAMI competition, where it was used to visualize the most difficult profile outputs to correctly classify taxa. In this article, TAMPA was validated on simulated data, with the corresponding ground truth not revealed to the taxonomic profilers while generating the output profiles. Using simulated data was necessary in order to know the true distribution of the ground truth. While TAMPA can be used for hypothesis generation on real biological data, further analysis and follow-up studies will be required to validate the hypotheses generated by TAMPA.

## Methods

TAMPA was run on the profiling datasets generated in the CAMI challenge. The profile files were extracted from the GitHub repo of the CAMI challenge: https://github.com/CAMI-challenge/firstchallenge_evaluation/tree/master/profiling/data/profile_submissions. The description.property file, found in the corresponding subdirectory of each tool at https://github.com/CAMI-challenge/firstchallenge_evaluation/tree/master/profiling/data/profile_submissions, was used to map the anonymous name to the tool name. We limited our analysis to sample 1 of the high-complexity dataset, denoted by CAMI_HIGH_S001. We studied tools with the highest and lowest precision, recall, and UniFrac score. The following command was used to run TAMPA:

python src/tampa.py -i tool.profile -g ground_truth rank -s CAMI_HIGH_S001 -b basename -k linear -r 1600 -o.

## Implementation and Features

The workflow of TAMPA is as follows: TAMPA takes in a series of input profile files for comparison from the user, along with several customizable visualization options. For each sample, the profile files and the NCBI taxdump database are used to create the percentage abundance predictions for each taxon. TAMPA then compares the percentage abundances of each tool to compute the relative abundances. These relative abundances are then used as input to the tree-building pipeline, which uses the ete3 toolkit to make the desired plot. The output file is rendered and saved according to the user-defined parameters.

Users can run TAMPA in 2 modes: a normal mode, which compares the output of several profilers and displays the relative abundance, and a contrast mode, which highlights the false positives and false negatives. For an input to TAMPA, the users can define the number of inputs (0–3), the sample of interest, and the threshold, at which the differences will be highlighted. TAMPA allows users to choose among multiple graph layout formats, including pie, bar, circle, and rectangular. Users can further customize the graph by choosing the scaling options for the graph (log, sqrt, power) and other parameters such as the vertical branch margin, leaf separation, label font size, figure width and height, and image resolution. In cases where the number of samples is very large and the graph becomes crowded, users can choose to display only the nodes with abundance higher than a particular threshold and/or add labels to specific parts of the graph such as only the leaf nodes. Users can also choose if they want to plot the L1 error or normalize the relative abundances of the samples. TAMPA allows users to analyze 1 or more samples of interest and allows for the analysis of both single-input taxonomic profiles, as well as input profiles with the ground truth. Users can also choose to decide alternate taxonomies and restrict visualization to a particular taxonomic rank. It can be used to study the impact of filtering low-abundance taxa. While the default database used for reading the input is the ncbi taxdump database, the users can specify a different database dump file. A comprehensive list of user-defined parameters and their descriptions can be produced by running:

python tampa.py –help

## Supplementary Material

giad008_GIGA-D-22-00116_Original_Submission

giad008_GIGA-D-22-00116_Revision_1

giad008_GIGA-D-22-00116_Revision_2

giad008_Response_to_Reviewer_Comments_Original_Submission

giad008_Response_to_Reviewer_Comments_Revision_1

giad008_Reviewer_1_Report_Original_Submission Alessio Milanese -- 5/26/2022 Reviewed

giad008_Reviewer_1_Report_Revision_1 Alessio Milanese -- 11/22/2022 Reviewed

giad008_Reviewer_2_Report_Original_Submission Francesco Asnicar, Ph.D. -- 6/7/2022 Reviewed

giad008_Reviewer_2_Report_Revision_1 Francesco Asnicar, Ph.D. -- 11/24/2022 Reviewed

giad008_Supplemental_File

## Data Availability

TAMPA was run on the profile files produced by the top- and bottom-performing taxonomic profilers. The taxonomic profiles represent the taxonomic identities and relative abundances of microbial community members from metagenome samples. The profiling files used to run TAMPA are freely available on the GitHub repo of the CAMI challenge [32, 33]. Benchmarking data from the CAMI challenge are available on GigaDB [34].

## Additional Files

**Supplementary Fig. S1**. Visualization of the taxonomic profiles of tools with identical UniFrac scores of 4, Taxy_pro versus Metaphyler, using TAMPA on the CAMI dataset at the class rank. Note the differences in taxa predictions, even though the tools have identical UniFrac scores.

**Supplementary Fig. S2**. Visualization of the taxonomic profiles of tools with identical UniFrac scores of 4, Taxy_pro versus Metaphyler, using TAMPA on the CAMI dataset at the order rank.

**Supplementary Fig. S3**. Visualization of the taxonomic profiles of tools with identical UniFrac scores of 4, Taxy_pro versus Metaphyler, using TAMPA on the CAMI dataset at the family rank.

**Supplementary Fig. S4**. Visualization of the taxonomic profiles of tools with identical UniFrac scores of 4, Taxy_pro versus Metaphyler, using TAMPA on the CAMI dataset at the genus rank.

**Supplementary Fig. S5**. Visualization of the taxonomic profiles of tools with identical UniFrac scores of 4, Taxy_pro versus Metaphyler, using TAMPA on the CAMI dataset at the species rank.

**Supplementary Fig. S6**. Visualization of the taxonomic profiles of a top-performing CAMI tool, Metaphyler versus the ground truth, using TAMPA on the CAMI dataset at the class level.

**Supplementary Fig. S7**. Visualization of the taxonomic profiles of a top-performing CAMI tool, Metaphyler versus the ground truth, using TAMPA on the CAMI dataset at the order level.

**Supplementary Fig. S8**. (A) Visualization of the taxonomic profiles of a top-performing CAMI tool, Metaphyler versus the ground truth, using TAMPA on the CAMI dataset at the family level. (B) With contrast mode.

**Supplementary Fig. S9**. (A) Visualization of the taxonomic profiles of a top-performing CAMI tool, Metaphyler versus the ground truth, using TAMPA on the CAMI dataset at the genus level. (B) With contrast mode.

**Supplementary Fig. S10**. Visualization of the taxonomic profiles of a top-performing CAMI tool, Metaphyler versus the ground truth, using TAMPA on the CAMI dataset at the species level. (B) With contrast mode.

**Supplementary Fig. S11**. Visualization of the taxonomic profiles of the lowest-performing tool, mOTU versus the ground truth, using TAMPA on the CAMI dataset at the class level.

**Supplementary Fig. S12**. Visualization of the taxonomic profiles of the lowest-performing tool, mOTU versus the ground truth, using TAMPA on the CAMI dataset at the order level.

**Supplementary Fig. S13**. (A) Visualization of the taxonomic profiles of the lowest-performing tool, mOTU versus the ground truth, using TAMPA on the CAMI dataset at the family level. (B) With contrast mode.

**Supplementary Fig. S14**. (A) Visualization of the taxonomic profiles of the lowest-performing tool, mOTU versus the ground truth, using TAMPA on the CAMI dataset at the genus level. (B) With contrast mode.

**Supplementary Fig. S15**. Visualization of the taxonomic profiles of the lowest-performing tool, mOTU versus the ground truth, using TAMPA on the CAMI dataset at the species level. (B) With contrast mode.

**Supplementary Fig. S16**. Incorporation of TAMPA into OPAL.

**Supplementary Fig. S17**. Visualization of the taxonomic profiles of 3 tools: Taxy_pro (green) versus Metaphyler (orange) versus mOUT (blue), using TAMPA on the CAMI dataset at the class rank.

## Abbreviations

CAMI: Critical Assessment of Metagenome Interpretation; TAMPA: Taxonomic metagenome profiling evaluation; WGS: whole-genome sequencing.

## Code Availability

Project name: TAMPA (TAxonoMic Profiling Analysis)

Project homepage: https://github.com/dkoslicki/TAMPA

Operating system(s): unix based

Other requirements: etetoolkit, python = 3.7, numpy, ete3, seaborn, pandas, matplotlib, biom-format

License: MIT

RRID: SCR_023201

Biotools: tampa

TAMPA is provided in a platform-independent fashion via Bioconda [35]:

Bioconda link: https://anaconda.org/vsarwal/tampa

as well as integrated into the Galaxy Toolshed [36] for easy “point-and-click” analysis for less computationally inclined users:

Galaxy Toolshed link: https://toolshed.g2.bx.psu.edu/repository?repository_id = 7b5054a8c1e84051

All code required to produce the figures and analysis performed in this article is freely available at [37].

An archival copy of the code is also available via the GigaScience database GigaDB [38].
