# Supplementary material for: TAMPA: interpretable analysis and visualization of metagenomics-based taxon abundance profiles
Source: Gigascience. 2023 Feb 28;12:giad008. doi: 10.1093/gigascience/giad008 (PMC9972184; doi:10.1093/gigascience/giad008)
Supplement: giad008_GIGA-D-22-00116_Revision_1 [file giad008_giga-d-22-00116_revision_1.pdf]

GigaScience

TAMPA: interpretable analysis and visualization of metagenomics-based taxon abundance profiles

--Manuscript Draft--

Manuscript Number:	GIGA-D-22-00116R1
Full Title:	TAMPA: interpretable analysis and visualization of metagenomics-based taxon abundance profiles
Article Type:	Technical Note
Funding Information:	
Abstract:	Abstract<br><br>Metagenomic taxonomic profiling aims to predict the identity and relative abundance of taxa in a given whole genome sequencing metagenomic sample. A recent surge in computational methods that aim to accurately estimate taxonomic profiles, called taxonomic profilers, have motivated community driven efforts to create standardized benchmarking datasets and platforms, standardized taxonomic profile formats, as well as a benchmarking platform to assess tool performance. While this standardization is essential, there is currently a lack of tools to visualize the standardized output of the many existing taxonomic profilers. Thus, benchmarking studies rely on a single value metrics to compare performance of tools and compare to benchmarking datasets. This is one of the major problems in analyzing metagenomic profiling data, since single metrics, such as the F1 score, fail to capture the biological differences between the datasets. Here we report the development of TAMPA (Taxonomic metagenome profiling evaluation) , a robust and easy-to-use method that allows scientists to easily interpret and interact with taxonomic profiles produced by the many different taxonomic profiler methods beyond the standard metrics used by the scientific community. We demonstrate the unique ability of TAMPA to generate novel biological hypothesis by highlighting the taxonomic differences between samples otherwise missed by commonly utilized metrics. Additionally, we show that TAMPA can help visualize the output of taxonomic profilers, enabling biologists to effectively choose the most appropriate profiling method to use on their metagenomics data. TAMPA is available on GitHub, Bioconda and Galaxy Toolshed at https://github.com/dkoslicki/TAMPA, and is released under the MIT license.
Corresponding Author:	Serghei Mangul<br>University of Southern California<br>Santa Monica, California UNITED STATES
Corresponding Author Secondary Information:	
Corresponding Author's Institution:	University of Southern California
Corresponding Author's Secondary Institution:	
First Author:	Varuni Sarwal
First Author Secondary Information:	
Order of Authors:	Varuni Sarwal
	Jaqueline Brito
	Serghei Mangul
	David Koslicki
Order of Authors Secondary Information:	
Response to Reviewers:	Reviewer 1 Comment 1:<br>The tool should be easy to install, I would suggest to add it to bioconda, which allow the installation of all dependencies (see CODE EVALUATION below).<br>CODE EVALUATION --

Powered by Editorial Manager® and ProduXion Manager® from Aries Systems Corporation

I downloaded with conda (`conda install -c vsarwal tampa`) and have the following error:
$ profile_to_plot
Traceback (most recent call last):
  File "/miniconda3/bin/profile_to_plot", line 33, in <module>
    sys.exit(load_entry_point('CAMIViz==0.1.0', 'console_scripts', 'profile_to_plot')())
  File "/miniconda3/bin/profile_to_plot", line 25, in importlib_load_entry_point
    return next(matches).load()
  File "/miniconda3/lib/python3.7/site-packages/importlib_metadata/__init__.py", line 167, in load
    module = import_module(match.group('module'))
  File "/miniconda3/lib/python3.7/importlib/__init__.py", line 127, in import_module
    return _bootstrap._gcd_import(name[level:], package, level)
  File "<frozen importlib._bootstrap>", line 1006, in _gcd_import
  File "<frozen importlib._bootstrap>", line 983, in _find_and_load
  File "<frozen importlib._bootstrap>", line 967, in _find_and_load_unlocked
  File "<frozen importlib._bootstrap>", line 677, in _load_unlocked
  File "<frozen importlib._bootstrap_external>", line 728, in exec_module
  File "<frozen importlib._bootstrap>", line 219, in _call_with_frames_removed
  File "/miniconda3/lib/python3.7/site-packages/src/profile_to_plot.py", line 2, in <module>
    from ProfilesLayout import ProfilesLayout
ModuleNotFoundError: No module named 'ProfilesLayout'

I have the same error if I follow the installation in https://github.com/dkoslicki/TAMPA.

In order to make the tool easier to use I would suggest to:
- change the name of the executable from `profile_to_plot` to `tampa`
- you could add the package to bioconda to have all the dependencies installed
- change the version to 1.0.0 for the release

Response: We thank the reviewer for pointing out these errors. We have added the package on bioconda with dependencies installed, changed the name of the executable from profile_to_plot to tampa, and changed the version to be 1.0.0 for the release.

Reviewer 1 Comment 2:
TAMPA has limited applicability when comparing to Ground Truth. The tool allow to compare two different profiles, but there are conceptually two different evaluations: 1) compare two different profiles, and 2) compare a profile to the Ground Truth. The current implementation works good for the first evaluation, but I can see it can be improved for the second. For example, it would be interesting to visualise as a red circle false positive taxa (for example in Figure 2 Cyanobacteria), in blue false negatives (Nitrospirae in Figure 2) and in a gradient of white to green when the taxa expected from the Ground truth is measured: in white if the relative abundance is the same and in green if the relative abundance is different between expected and measured (with the colour intensity proportional to the error). In this way all correct taxa will be white, and looking at the tree colors we could understand the issue: red (false positive), blue (false negative), green (measure the correct taxa, but with wrong relative abundance). In this way, it would be much easier to spot issues in the tree, which is one of the main applications of TAMPA within the CAMI benchmark. This would be even more beneficial when there are large trees (example Figure S3), to quickly identify which part of the tree is the most problematic.

Response: We thank the reviewer for the great ideas. We have created a new mode for TAMPA called “contrast”, for better visualization of the comparison of the profile output with the ground truth, with a color spectrum. We have updated figure 2,3 with plots using the contrast mode, as well as added new text and images to the results and supplementary section (highlighted in red in the updated manuscript).

Reviewer 1 Comment 3:
In the main text there are references to Figure 1b and 1c, but the figures are labeled 2 and 3 instead.

Response: We thank the reviewer for pointing this out. We have changed the references to figure 2 and 3.

Reviewer 1 Comment 4:
The paper present a tool, as such I would expect a more detailed description of the tool implementation and options in the methods. This would be helpful for people to use the tool.

Response: We thank the reviewer for their comment. We have created a new subsection in the Methods section, called Implementation and Features (highlighted in red in the updated manuscript), outlining how the tool has been implemented, and the various options available to the user.

Reviewer 2 Comment 1:
It would be interesting to see (if possible) the comparison of three (or more) taxonomic profiles at the same time. The evaluations shown are always binary, but in a real-case scenario where a user would like to evaluate 3 or 4 different taxonomic profiling tools on his community, it would be great to be able to do it.

Response: We thank the reviewer for their comment. We have modified the tool so that 3 tools can be compared at once. Visualization of 4 and more tools is out of scope of this paper, we recommend that users perform multiple comparisons using TAMPA for improved visibility. We have added new figures and text to the results section (highlighted in red in the updated manuscript).

Reviewer 2 Comment 2:
Other than the evaluation on the agreement between two (or more) taxonomic profiling tools, it is not clear how TAMPA can drive improvement over biologically-relevant question. Although it is clear, as the authors stated in the introduction, that different taxonomic profilers (with different parameters settings) can produce very different taxonomic representations, to support this statement it will be important to be able to show, at least one case, where TAMPA can suggest a different taxonomic interpretation of a microbial community that is also biologically relevant.

Response: We thank the reviewer for this insightful comment. We have included a new subsection in our Results section containing an example where results from TAMPA is able to provide additional biologically relevant insights compared to standard metrics of a microbial community that are biologically relevant. In particular, we applied the two best-performing taxonomic profilers MetaPhyler 2.2 and mOTUs2.5.1 to the marine dataset published in the “Critical Assessment of Metagenome Interpretation: the second round of challenges” (https://doi.org/10.1038/s41592-022-01431-4). On comparing the output of MetaPhlAn with the gold standard, we use TAMPA to demonstrate that while MetaPhlAn was one of the top-performing profilers, it was not able to detect several clades, including Planctomycetes (Figure 4a). Additionally, while MetaPhlAn and mOTUs were both the top-performing profilers in terms of single-valued metrics such as F1-score and L1 norm error, there were several critical differences between their outputs, highlighted by TAMPA at the phylum level (Figure 4 b). For example, mOTUs failed to detect the Tenericutes group, while MetaPhlAn failed to detect Planctomycetes. There was also a considerable difference in the percentage abundance of other groups, such as Firmicutes.Note that this data set is simulated, though the corresponding ground truth was not revealed to the taxonomic profilers. Using simulated data is required in this setting since we need to know for sure what the “correct” answer is. If we used real biological data, further analyses and followup studies would be required to validate the hypothesis generated by TAMPA, which is beyond the scope of this manuscript. We have added text to the discussion mentioning how TAMPA can be used for hypothesis generation, since confirming biological discoveries certainly requires more analyses than the visualizations returned by TAMPA.

Powered by Editorial Manager® and ProduXion Manager® from Aries Systems Corporation

	Reviewer 2 Comment 3:<br>Figures in general appear to be of low-quality and stretched, please consider improving them as they are the main point of TAMPA.<br><br>Response: We thank the reviewer for this insight. We have replaced the figures in the manuscript with high-quality figures. We have also attached the high-quality figures separately in our submission for your reference.
Additional Information:	
Question	Response
Are you submitting this manuscript to a special series or article collection?	No
**Experimental design and statistics**<br><br>Full details of the experimental design and statistical methods used should be given in the Methods section, as detailed in our Minimum Standards Reporting Checklist. Information essential to interpreting the data presented should be made available in the figure legends.<br><br>Have you included all the information requested in your manuscript?	Yes
**Resources**<br><br>A description of all resources used, including antibodies, cell lines, animals and software tools, with enough information to allow them to be uniquely identified, should be included in the Methods section. Authors are strongly encouraged to cite Research Resource Identifiers (RRIDs) for antibodies, model organisms and tools, where possible.<br><br>Have you included the information requested as detailed in our Minimum Standards Reporting Checklist?	No
If not, please give reasons for any omissions below.<br><br>as follow-up to "**Resources**	n/a

Powered by Editorial Manager® and ProduXion Manager® from Aries Systems Corporation

A description of all resources used, including antibodies, cell lines, animals and software tools, with enough information to allow them to be uniquely identified, should be included in the Methods section. Authors are strongly encouraged to cite Research Resource Identifiers (RRIDs) for antibodies, model organisms and tools, where possible.<br><br>Have you included the information requested as detailed in our Minimum Standards Reporting Checklist?<br><br>"	
**Availability of data and materials**<br><br>All datasets and code on which the conclusions of the paper rely must be either included in your submission or deposited in publicly available repositories (where available and ethically appropriate), referencing such data using a unique identifier in the references and in the “Availability of Data and Materials” section of your manuscript.<br><br>Have you have met the above requirement as detailed in our Minimum Standards Reporting Checklist?	Yes

Powered by Editorial Manager® and ProduXion Manager® from Aries Systems Corporation

Manuscript Click here to access/download;Manuscript;TAMPA.docx

TAMPA: interpretable analysis and visualization of metagenomics-based taxon abundance profiles

Varuni Sarwal[1], Jaqueline Brito[2], Serghei Mangul[2,3*], David Koslicki[4,5,6*]

1. Department of Computer Science, University of California Los Angeles, 580 Portola Plaza, Los Angeles, CA 90095, USA
2. Department of Clinical Pharmacy, University of Southern California, Los Angeles, California, 90089, United States
3. Department of Quantitative and Computational Biology, USC Dornsife College of Letters, Arts and Sciences University of Southern California, Los Angeles, California, 90089, United States
4. Department of Computer Science and Engineering, The Pennsylvania State University, University Park, PA, USA
5. Department of Biology, The Pennsylvania State University, University Park, PA, USA
6. Huck Institutes of the Life Sciences, The Pennsylvania State University, University Park, PA, USA

* These authors jointly supervised this work.

Abstract

Metagenomic taxonomic profiling aims to predict the identity and relative abundance of taxa in a given whole genome sequencing metagenomic sample. A recent surge in computational methods that aim to accurately estimate taxonomic profiles, called taxonomic profilers, have motivated community driven efforts to create standardized benchmarking datasets and platforms, standardized taxonomic profile formats, as well as a benchmarking platform to assess tool performance. While this standardization is essential, there is currently a lack of tools to visualize the standardized output of the many existing taxonomic profilers. Thus, benchmarking studies rely on a single value metrics to compare performance of tools and compare to benchmarking datasets. This is one of the major problems in analyzing metagenomic profiling data, since single metrics, such as the F1 score, fail to capture the biological differences between the datasets. Here we report the development of TAMPA (**Ta**xonomic **m**etagenome **p**rofiling evalu**a**tion) , a robust and easy-to-use method that allows scientists to easily interpret and interact with taxonomic profiles produced by the many different taxonomic profiler methods beyond the standard metrics used by the scientific community. We demonstrate the unique ability of TAMPA to generate novel biological hypothesis by highlighting the taxonomic differences between samples otherwise missed by commonly utilized metrics. Additionally, we show that TAMPA can help visualize

the output of taxonomic profilers, enabling biologists to effectively choose the most appropriate profiling method to use on their metagenomics data. TAMPA is available on GitHub, Bioconda and Galaxy Toolshed at https://github.com/dkoslicki/TAMPA, and is released under the MIT license.

Introduction

Microorganisms live in complex communities and play a vital role in human and environmental health. Studying these communities is important to understand how microbes interact with each other, their host, and the environment. Metagenomics has become an essential tool to study microbiomes due to improvements in technology and bioinformatic algorithms. One of the first steps in investigating microbial community dynamics is to estimate the abundance of different species in the community; this process is called taxonomic profiling. Taxonomic metagenome profiling aims to predict the identity and relative abundances of taxa in a given whole genome sequencing (WGS) metagenomic sample. A recent surge in computational methods that aim to accomplish this, called taxonomic profilers, have motivated community-driven efforts to create standardized benchmarking datasets [11–3], standardized taxonomic profile formats [4], as well as a benchmarking platform to assess tool performance on simulated data [5]. While this standardization is essential, there is currently a lack of tools to visualize the standardized output of the many existing taxonomic profilers, and benchmarking studies rely on a single value metrics to compare performance of tools and compare to benchmarking datasets. Indeed, the only two such WGS taxonomic profiling visualization and analysis tools that do exist are either integrated into a single taxonomic profiling method [6], or else lack the flexibility and interpretability for the analysis and visualization of multiple taxonomic profiles [7]. Neither of these methods are designed for or compatible with the community-driven output formats previously mentioned.

Despite the availability of flexible and interactive visualization tools in the area of amplicon microbial analysis (such as 16S rRNA studies), similar methods are yet to be developed for WGS metagenomics. For example, metacoder [8] is a tool that allows for visualizing, analyzing, and manipulating amplicon microbial data. However, metacoder is not designed for WGS metagenomic analyses and cannot be used for analysis and visualizing metagenomic taxonomic profiles due to amplicon analyses relying on Operational Taxonomic Units, a concept that is not relevant to metagenomic studies. Similarly, the recently published preprint for the software package EMPress[9] is an interactive phylogenetic tree viewer not explicitly intended for the visualization of WGS taxonomic profiles.

Additionally, lack of tools that provide an interpretable visualization of multiple taxonomic profiles limits the ability of the biomedical community to select a tool. As such, when WGS metagenomic data is generated and a scientist wishes to determine which of the dozens [10–23] of taxonomic profilers to use, they typically rely on benchmark studies [1,24,25] . These benchmark studies often use simulated data that does not accurately reflect their samples of interest. Alternatively, they can run their own simulation and benchmarking study tailor to their use-case, but this requires significant time investment [2]. Scientists often resort to simply picking a familiar tool regardless of its performance characteristics. Given the substantial variability in the performance of taxonomic profiling tools [1,24,25], this may result in misinterpretation of their data and can potentially lead to unfortunate situations where utilizing a single low accuracy taxonomic profiling tool can lead to an interpretation of data [26] (i.e. presence of Bubonic plague in the New York subway system) that is later to be found to be inaccurate [27].

To empower biomedical researchers with a robust and easy to use metagenomic taxonomic profile analysis and visualization platform, we have developed a software package TAMPA (Taxonomic metagenome profiling evaluation). Our platform assists scientists in contextualizing, assessing, and extracting insight from taxonomic profiles produced by multiple taxonomic profilers when applied to either real or simulated data. TAMPA is designed to allow users to effectively analyze one or more taxonomic profiles produced by any of the numerous taxonomic profiling methods. Additionally, TAMPA can operate on the widely utilized and community developed BIOM [29] and CAMI [1] profiling formats. We demonstrate the utility of TAMPA by showing how it illuminates the important biological differences between samples and conditions otherwise missed by commonly utilized statistical metrics. When gold standard taxonomic profiles are available, we show how TAMPA can augment existing benchmarking platforms such as OPAL by being incorporated within the tool and providing an interpretable visualization of the profiles [5]. Additionally, we show that TAMPA can enable biologists to choose an appropriate profiling method to use on their real data when a ground truth taxonomic profile is not available, since TAMPA allows users to quickly ascertain similarities or differences in predictions made by multiple taxonomic tools.

Results

TAMPA is a computational tool that allows the user to effectively visualize one or more taxonomic profiles produced by taxonomic profiling methods. TAMPA contextualizes, assesses, and extracts insight

from multiple taxonomic profiler results. Here, we demonstrate three major ways in which TAMPA provides a novel way to visualize the outputs of existing profilers and visualization platforms.

TAMPA enables effective comparison of the outputs of multiple profilers

The Critical Assessment of Metagenome Interpretation(CAMI)[1–3] provides the most comprehensive and in-depth evaluation of metagenomic profiling, binning, and assembly methods to date. In the profiling competition, many of the most well-known profiling methods were evaluated on a variety of simulated datasets that modeled real-life challenges, such as various community diversities and confounding sequences from high-abundance plasmids and novel viral strains. To demonstrate the ability of TAMPA to provide an interpretable analysis and visualization of metagenomics-based taxon abundance profiles, we apply it to the results of three profiles from the publicly available CAMI dataset[1–3]: MetaPhyler [14]; mOTU [15]; and Taxy-Pro [30].

TAMPA has 2 major modes for comparing output profiles. First, TAMPA can be used to compare the outputs of multiple profilers and reveal insight even when traditional metrics report no differences. TAMPA does this by computing the percentage relative abundance per taxa and identifying which specific clades contributed to metric values, thus revealing biological differences that could otherwise be overlooked when looking only at single-valued metrics. We choose two profilers with an identical UniFrac score on a particular sample, Taxy-Pro and Metaphyler, and demonstrate the specific differences in their predictions of taxonomic profiles using TAMPA on the phylum level (Figure 1), as well as other taxonomic levels (Figure S1-S5). TAMPA can support up to 3 input profilers at once, illuminating differences in their relative abundances (Figure S17).

Second, even when tools performance is distinguishable by traditional numerical metrics, TAMPA can be used to quickly ascertain how tool predictions differ from the ground truth profile. For example, we chose both the top (Figure 2) and bottom (Figure 3) performing tools in terms of the L1 Norm, according to the CAMI challenge: MetaPhyler, and mOTU, and demonstrate that TAMPA can illuminate important biological differences between the two tools and the ground truth at the phylum level (Figure 2,3), as well as at all other taxonomic ranks (Figure S6-S15). To better visualize the differences between the tool and the ground truth, we have created a special “contrast mode” in TAMPA. In the contrast mode, the false positive taxas are represented as red circles, the false negative taxas as blue circles, true positives as white, and the remaining taxas in a gradient of white to green, with the color intensity proportional

to the relative error. This option is especially helpful when there are large trees, to identify problematic subregions (Figure S8-10, S13-15).

TAMPA augments existing benchmarking platforms

Third, TAMPA can be used to augment existing benchmarking platforms. We have integrated TAMPA into the taxonomic profiling benchmarking platform OPAL[5] in order to provide biological insight when scientists and tool developers aim to benchmark and compare taxonomic profilers (Figure S16). OPAL is a popular web-based tool used to compute commonly used performance metrics for profiler outputs. While OPAL provides global metrics and visualizations, it is unable to provide specific information on the taxonomic differences in the profiles. Additionally, scientists can encounter difficulty when interpreting statistical measures of differences between the estimated taxonomic frequencies and the ground truth, as well as when comparing differences between tools. With the inclusion of TAMPA in OPAL, users can now quickly ascertain the performance of the tools being analyzed at a level of resolution not possible before. For example, by utilizing the figures returned by TAMPA, a user can quantify tool performance on a particular taxonomic clade of interest. Based on our results (eg., Supplementary Figure S1), we show that TAMPA can highlight important taxonomic differences easily missed by statistical metrics, thus enabling biologists to choose the most appropriate profiling method to use on their data.

Marine metagenome prediction: a concept challenge

Microbial communities are key drivers of marine biogeochemistry, and this will improve our understanding of the distribution of organisms in the oceans as well as the selective forces that structure community composition and distribution across space and time. We applied the TAMPA on the results of the two best-performing taxonomic profilers MetaPhlAn 2.9.22 and mOTUs2.5.1 on the marine dataset published in the "Critical Assessment of Metagenome Interpretation: the second round of challenges" demonstrate a case where TAMPA can provide a different taxonomic interpretations of a microbial community that are biologically relevant. On comparing the output of MetaPhlAn with the gold standard, we use TAMPA to demonstrate that while MetaPhlAn was one of the top-performing profilers, it was not able to detect several clades, including Planctomycetes (Figure 4a). Additionally, while MetaPhlAn and mOTUs were both the top-performing profilers in terms of single-valued metrics such as F1-score and L1 norm error, there were several critical differences between their outputs, highlighted by TAMPA at the phylum level (Figure 4 b). For example, mOTUs failed to detect the

Tenericutes group, while MetaPhlAn failed to detect Planctomycetes. There was also a considerable difference in the percentage abundance of other groups, such as Firmicutes.

Discussion

Metagenomics has emerged as a technology of choice for analyzing microbial communities, with thousands of WGS metagenomic samples being produced annually[31]. Taxonomic profiling is an important first step in analyzing metagenomic data since taxonomic profiles represent the taxonomic identities and relative abundances of microbial community members from metagenome samples. Comparing these taxonomic profiles with each other, as well as with the gold standard is a nontrivial task, and there are no existing tools that provide a rigorous and intuitive analysis. Hence, TAMPA will be of broad interest to all scientists engaged in such research, thus allowing them to quickly contextualize, assess, and extract insight from taxonomic profiles instead of relying primarily on statistical summaries or manual manipulation. Indeed, TAMPA was effectively applied in the second round of the Critical Assessment of Metagenome Interpretation (CAMI) competition where it was used to visualize the most difficult profile outputs to correctly classify taxa. In this paper, TAMPA was validated on simulated data, with the corresponding ground truth not revealed to the taxonomic profilers while generating the output profiles. Using simulated data was necessary in order to know the true distribution of the ground truth. While TAMPA can be used for hypothesis generation on real biological data, further analysis and follow up studies will be required to validate the hypotheses generated by TAMPA.

Figures

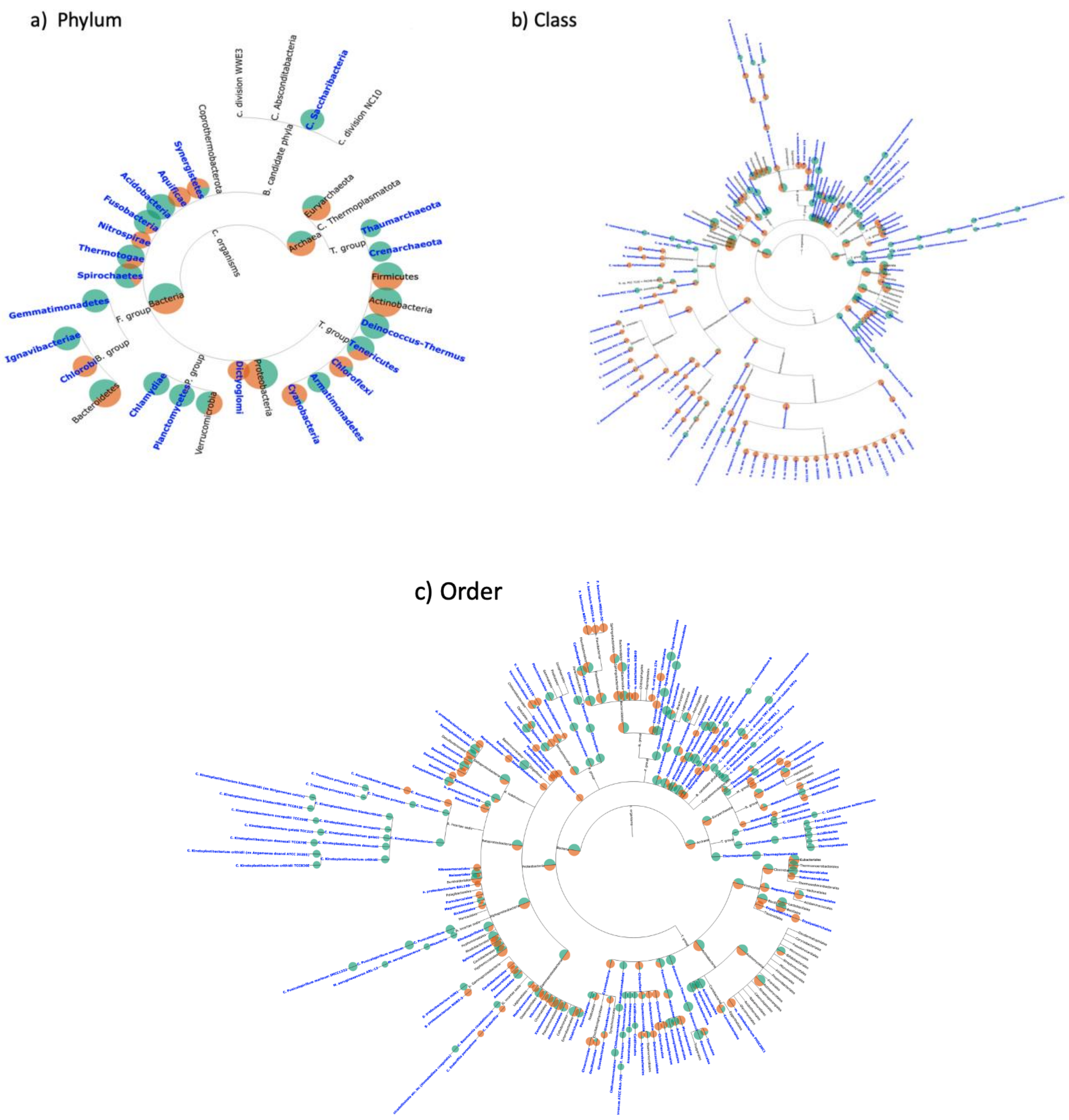

Figure 1: Visualization of the taxonomic profiles of **tools with identical UniFrac scores** of 4, Taxy-Pro (green) vs MetaPhyler (orange) using TAMPA on the CAMI dataset at the phylum, class, and order level. The size of the discs represents the total amount of relative abundance at the corresponding clade in the output profiles. If the tool predictions agree, a disc is colored half orange and half teal. The proportion of teal to orange changes with respect to the disagreement in the prediction of that clade's relative abundance between the two tools being compared. Highlighted blue text represents clades where the difference between the relative abundances of the predictions exceeds 30%.

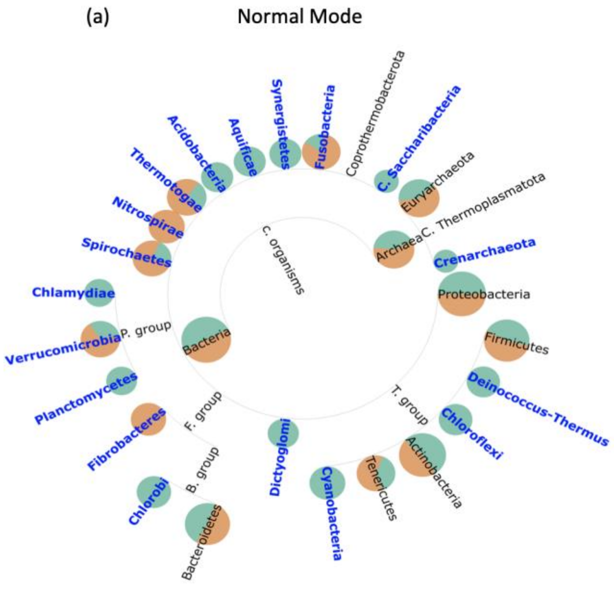

Figure 2: a) Visualization of the taxonomic profile of a **top performing CAMI tool** in terms of L1 norm, MetaPhyler (green) vs the ground truth (orange) using TAMPA on the CAMI dataset at the phylum level. The size of the discs represents the total amount of relative abundance at the corresponding clade in the ground truth, or the tool prediction if that clade is not in the ground truth. If the tool predictions agree, a disc is colored half orange and half teal. The proportion of teal to orange changes with respect to the disagreement in the prediction of that clade's relative abundance between the two tools being compared. Highlighted blue text represents clades where the difference between the relative abundances of the prediction and ground truth exceeds 30%.

b) Visualization of the taxonomic profile of a **top performing CAMI tool** in terms of L1 norm, MetaPhyler vs the ground truth using TAMPA on the CAMI dataset at the phylum level using the contrast mode. False positives are denoted in red, false negatives in blue, and a gradient of white to green when the taxa expected from the ground truth is measured: in white if the relative abundance is the same and in green if the relative abundance is different between expected and measured, with the color intensity proportional to the error.

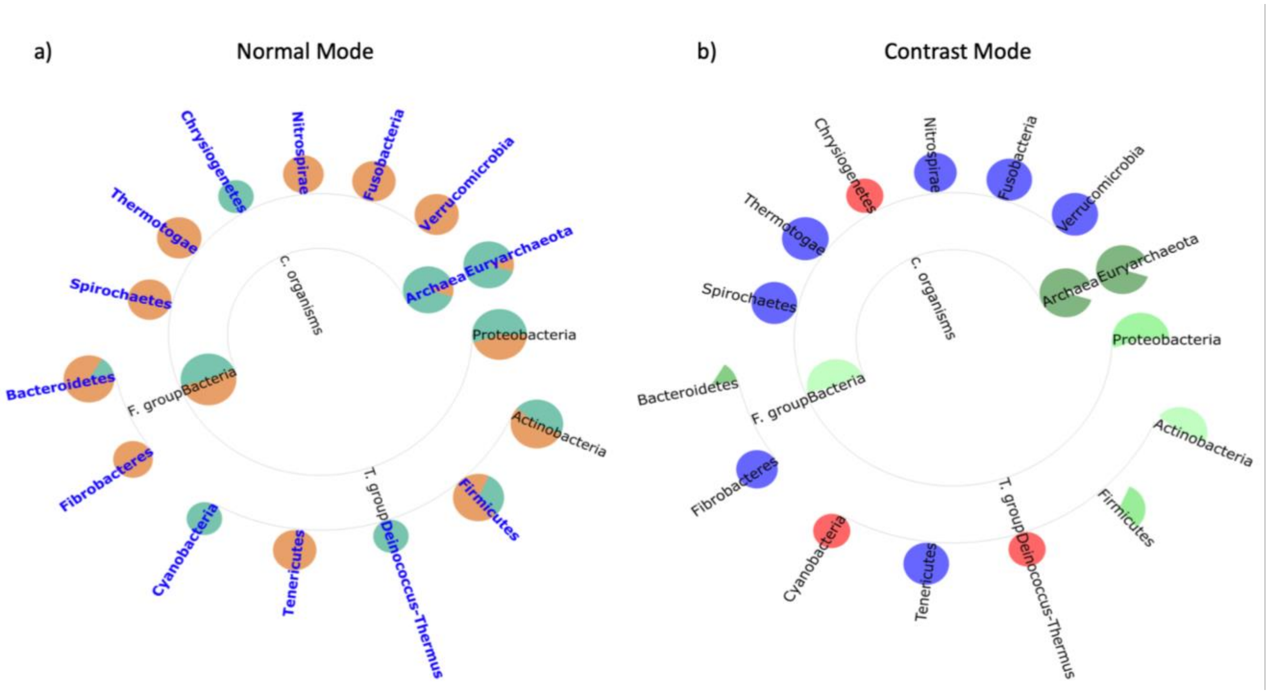

Figure 3: a) Visualization of the taxonomic profile of the **lowest performing tool in terms** of L1 norm, mOTU (green) vs the ground truth (orange) using TAMPA on the CAMI dataset at the phylum level. The size of the discs represents the total amount of relative abundance at the corresponding clade in the ground truth, or the tool prediction if that clade is not in the ground truth. If the tool predictions agree, a disc is colored half orange and half teal. The proportion of teal to orange changes with respect to the disagreement in the prediction of that clade's relative abundance between the two tools being compared. Highlighted blue text represents clades where the difference between the relative abundances of the prediction and ground truth exceeds 30%.

b) Visualization of the taxonomic profile of the **lowest performing tool in terms** of L1 norm, mOTU vs the ground truth using TAMPA on the CAMI dataset at the phylum level using the contrast mode. False positives are denoted in red, false negatives in blue, and a gradient of white to green when the taxa expected from the ground truth is measured: in white if the relative abundance is the same and in green

if the relative abundance is different between expected and measured, with the color intensity proportional to the error.

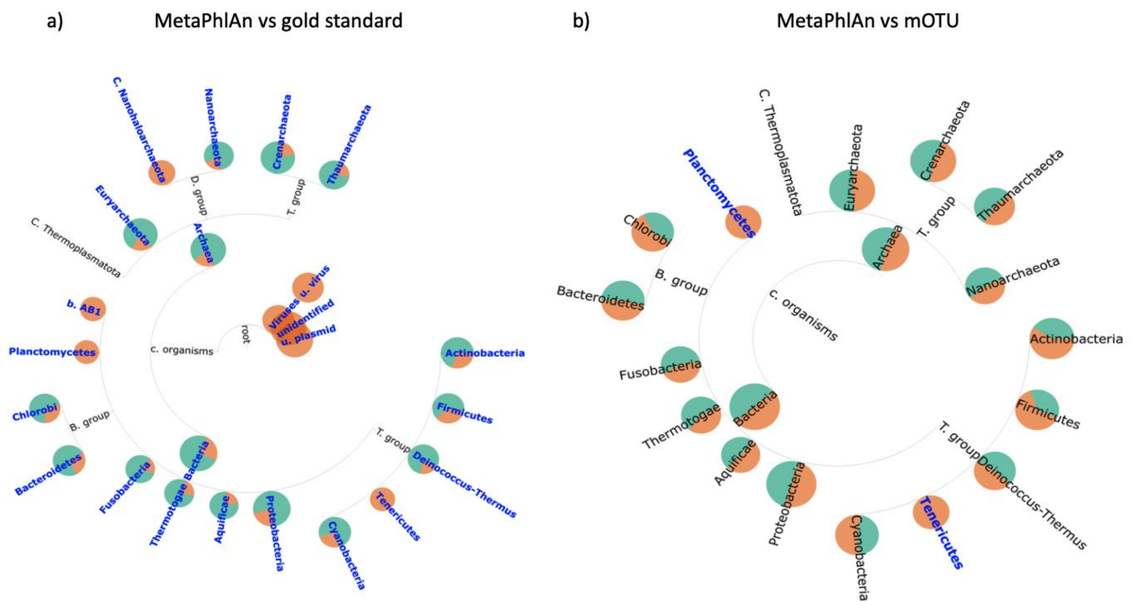

Figure 4: a) Visualization of the taxonomic profile of the **top performing tool in terms** of F1 score, MetaPhlAn (green) vs the ground truth (orange) using TAMPA on the CAMI marine dataset at the phylum level. The size of discs represents the total amount of relative abundance at the corresponding clade in the profiler outputs. If the tool predictions agree, a disc is colored half orange and half teal. The proportion of teal to orange changes with respect to the disagreement in prediction of that clade's relative abundance between the two tools being compared. Highlighted text represents clades where the difference between the relative abundances of the predictions exceeds 30%.

b) Visualization of the taxonomic profile of the **two top performing tools in terms** of F1 score, MetaPhlAn (green) and mOTUs (orange) on the marine dataset at the phylum level. The size of discs represents the total amount of relative abundance at the corresponding clade in the ground truth, or the tool prediction if that clade is not in the ground truth. If the tool predictions agree, a disc is colored half orange and half teal. The proportion of teal to orange changes with respect to the disagreement in prediction of that clade's relative abundance between the two tools being compared. Highlighted blue text represents clades where the difference between the relative abundances of the prediction and ground truth exceeds 30%.

Methods

TAMPA was run on the profiling datasets generated in the CAMI challenge. The profile files were extracted from the github repo of the CAMI challenge: https://github.com/CAMI-challenge/firstchallenge_evaluation/tree/master/profiling/data/profile_submissions. The description.property file, found in the corresponding subdirectory of each tool at https://github.com/CAMI-challenge/firstchallenge_evaluation/tree/master/profiling/data/profile_submissions was used to map the anonymous name to the tool name. We limited our analysis to Sample 1 of the high complexity dataset, denoted by CAMI_HIGH_S001. We studied tools with the highest and lowest precision, recall and UniFrac score. The following command was used to run TAMPA:

```
python src/tampa.py -i tool.profile -g ground_truth rank -s CAMI_HIGH_S001 -b basename -k linear -r 1600 -o .
```

Implementation and Features

The workflow of TAMPA is as follows: TAMPA takes in a series of input profile files for comparison from the user, along with several customizable visualization options. For each sample, the profile files and the NCBI taxdump database are used to create the percentage abundance predictions for each taxon. TAMPA then compares the percentage abundances of each tool to compute the relative abundances. These relative abundances are then used as input to the tree-building pipeline, which uses the ete3 toolkit to make the desired plot. The output file is rendered and saved according to the user-defined parameters.

Users can run TAMPA in two modes, a normal mode, which compares the output of several profilers and displays the relative abundance, and a contrast mode, which highlights the false positives and false negatives. For an input to TAMPA, the users can define the number of inputs (0-3), the sample of interest, and the threshold, at which the differences will be highlighted. TAMPA allows users to choose among multiple graph layout formats, including pie, bar, circle, and rectangular. Users can further customize the graph by choosing the scaling options for the graph (log, sqrt, power), and other parameters such as the vertical branch margin, leaf separation, label font size, figure width and height, and image resolution. In cases where the number of samples is very large and the graph becomes crowded, users can choose to display only the nodes with abundance higher than a particular threshold,

and/or add labels to specific parts of the graph such as only the leaf nodes. Users can also choose if they want to plot the L1 error or normalize the relative abundances of the samples. TAMPA allows users to analyze one or more samples of interest and allows for the analysis of both single input taxonomic profiles, as well as input profiles with the ground truth. Users can also choose to decide alternate taxonomies and restrict visualization to a particular taxonomic rank. It can be used to study the impact of filtering low abundance taxa. While the default database used for reading the input is the ncbi taxdump database, the users can specify a different database dump file. A comprehensive list of user-defined parameters and their descriptions can be produced by running:

```
python tampa.py --help
```

Data availability

TAMPA was run on the .profile files produced by the top and bottom performing taxonomic profilers. The taxonomic profiles represent the taxonomic identities and relative abundances of microbial community members from metagenome samples. The profiling files used to run TAMPA are freely available on the github repo of the CAMI challenge: https://github.com/CAMI-challenge/firstchallenge_evaluation

Code availability

TAMPA is provided in a platform independent fashion via Bioconda [33] :

Bioconda link: https://anaconda.org/vsarwal/tampa

as well as integrated into the Galaxy Toolshed [34] for easy "point and click" analysis for less computationally inclined users:

Galaxy Toolshed link: https://toolshed.g2.bx.psu.edu/repository?repository_id=7b5054a8c1e84051

The source code is available at:

https://github.com/dkoslicki/TAMPA

All code required to produce the figures and analysis performed in this paper are freely available at:

https://github.com/Addicted-to-coding/TAMPA_publication

References

1. Sczyrba, A. *et al.* Critical Assessment of Metagenome Interpretation-a benchmark of metagenomics software. *Nat. Methods* **14**, 1063–1071 (2017).

2. Meyer, F. *et al.* Tutorial: Assessing metagenomics software with the CAMI benchmarking toolkit. 2020.08.11.245712 (2020) doi:10.1101/2020.08.11.245712.

3. Mangul, S. *et al.* Systematic benchmarking of omics computational tools. *Nat. Commun.* **10**, 1393 (2019).

4. Meyer, F. *et al.* Tutorial: Assessing metagenomics software with the CAMI benchmarking toolkit. *Nat. Protoc.* (under revision).

5. Meyer, F. *et al.* Assessing taxonomic metagenome profilers with OPAL. *Genome Biol.* **20**, 51 (2019).

6. Asnicar, F., Weingart, G., Tickle, T. L., Huttenhower, C. & Segata, N. Compact graphical representation of phylogenetic data and metadata with GraPhlAn. PeerJ 3: e1029. (2015).

7. Ondov, B. D., Bergman, N. H. & Phillippy, A. M. Interactive metagenomic visualization in a Web browser. *BMC Bioinformatics* **12**, 385 (2011).

8. Foster, Z. S. L., Sharpton, T. J. & Grünwald, N. J. Metacoder: An R package for visualization and manipulation of community taxonomic diversity data. *PLoS Comput. Biol.* **13**, e1005404 (2017).

9. Cantrell, K. *et al.* EMPress enables tree-guided, interactive, and exploratory analyses of multi-omic datasets. 2020.10.06.327080 (2020) doi:10.1101/2020.10.06.327080.

10. Koslicki, D. & Falush, D. MetaPalette: a k-mer Painting Approach for Metagenomic Taxonomic Profiling and Quantification of Novel Strain Variation. *mSystems* **1**, (2016).

11. Piro, V. C., Lindner, M. S. & Renard, B. Y. DUDes: a top-down taxonomic profiler for metagenomics. *Bioinformatics* **32**, 2272–2280 (2016).

12. Silva, G. G. Z., Cuevas, D. A., Dutilh, B. E. & Edwards, R. A. FOCUS: an alignment-free model to identify organisms in metagenomes using non-negative least squares. *PeerJ* **2**, e425 (2014).

13. Segata, N. *et al.* Metagenomic microbial community profiling using unique clade-specific marker genes. *Nat. Methods* **9**, 811–814 (2012).

14.Liu, B., Gibbons, T., Ghodsi, M., Treangen, T. & Pop, M. Accurate and fast estimation of taxonomic profiles from metagenomic shotgun sequences. *BMC Genomics* **12 Suppl 2**, S4 (2011).

15.Sunagawa, S. *et al.* Metagenomic species profiling using universal phylogenetic marker genes. *Nat. Methods* **10**, 1196–1199 (2013).

16.Nguyen, N.-P., Mirarab, S., Liu, B., Pop, M. & Warnow, T. TIPP: taxonomic identification and phylogenetic profiling. *Bioinformatics* **30**, 3548–3555 (2014).

17.Lu, J., Breitwieser, F. P., Thielen, P. & Salzberg, S. L. Bracken: estimating species abundance in metagenomics data. *PeerJ Comput. Sci.* **3**, e104 (2017).

18.Koslicki, D., Foucart, S. & Rosen, G. WGSQuikr: fast whole-genome shotgun metagenomic classification. *PLoS One* **9**, e91784 (2014).

19.Milanese, A. *et al.* Microbial abundance, activity and population genomic profiling with mOTUs2. *Nat. Commun.* **10**, 1014 (2019).

20.Shi, L. & Chen, B. A Vector Representation of DNA Sequences Using Locality Sensitive Hashing. 726729 (2019) doi:10.1101/726729.

21.Marcelino, V. R. *et al.* CCMetagen: comprehensive and accurate identification of eukaryotes and prokaryotes in metagenomic data. *Genome Biol.* **21**, 103 (2020).

22.LaPierre, N., Alser, M., Eskin, E., Koslicki, D., Mangul, S. *Metalign: efficient alignment-based metagenomic profiling via containment min hash*. (Github).

23.Kim, D., Song, L., Breitwieser, F. P. & Salzberg, S. L. Centrifuge: rapid and sensitive classification of metagenomic sequences. *Genome Res.* **26**, 1721–1729 (2016).

24.McIntyre, A. B. R. *et al.* Comprehensive benchmarking and ensemble approaches for metagenomic classifiers. *Genome Biol.* **18**, 182 (2017).

25.Lindgreen, S., Adair, K. L. & Gardner, P. P. An evaluation of the accuracy and speed of metagenome analysis tools. *Sci. Rep.* **6**, 19233 (2016).

26.Afshinnekoo, E. *et al.* Erratum: Geospatial Resolution of Human and Bacterial Diversity with City-Scale Metagenomics. *Cell Syst* **1**, 72–87e (2015).

27.Ackelsberg, J. *et al.* Lack of Evidence for Plague or Anthrax on the New York City Subway. *Cell Syst* **1**, 4–5 (2015).

28.McClelland, J. & Koslicki, D. EMDUniFrac: exact linear time computation of the UniFrac metric and identification of differentially abundant organisms. *J. Math. Biol.* **77**, 935–949 (2018).

29.McDonald, D. *et al.* The Biological Observation Matrix (BIOM) format or: how I learned to stop worrying and love the ome-ome. *Gigascience* **1**, 7 (2012).

30.Klingenberg, H., Aßhauer, K. P., Lingner, T. & Meinicke, P. Protein signature-based estimation of metagenomic abundances including all domains of life and viruses. *Bioinformatics* **29**, 973–980 (2013).

31.Leinonen, R., Sugawara, H., Shumway, M. & International Nucleotide Sequence Database Collaboration. The sequence read archive. *Nucleic Acids Res.* **39**, D19–21 (2011).

32.Meyer, F. *et al.* Critical Assessment of Metagenome Interpretation - the second round of challenges. *bioRxiv* 2021.07.12.451567 (2021) doi:10.1101/2021.07.12.451567.

33.Grüning, B. *et al.* Bioconda: sustainable and comprehensive software distribution for the life sciences. *Nat. Methods* **15**, 475–476 (2018).

34.Blankenberg, D. *et al.* Dissemination of scientific software with Galaxy ToolShed. *Genome Biol.* **15**, 403 (2014).

32. Meyer, Fernando, et al. "Critical Assessment of Metagenome Interpretation: the second round of challenges." Nature Methods (2022): 1-12.

Supplementary Materials

Supplementary Figures

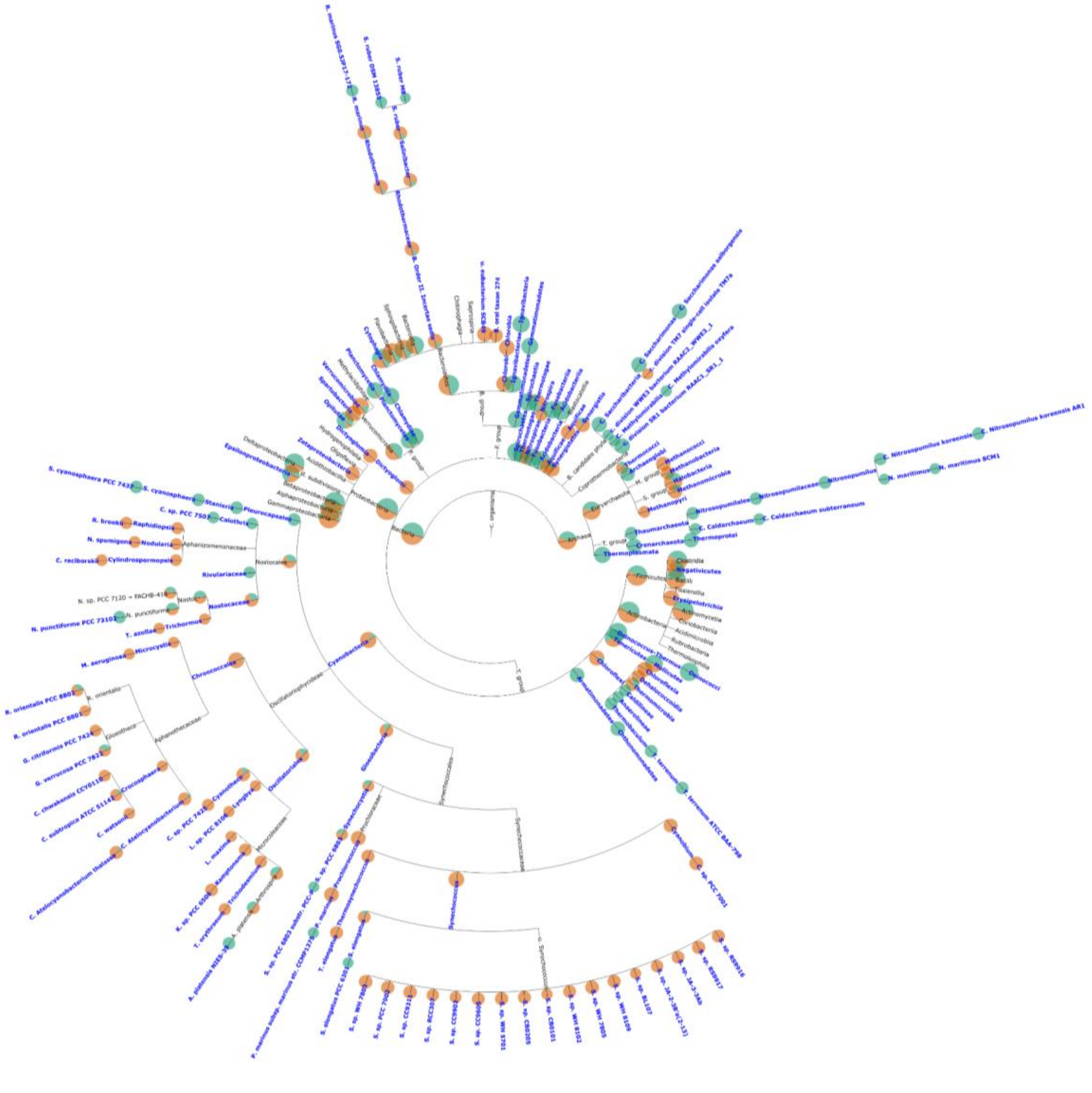

Figure S1: Visualization of the taxonomic profiles of tools with identical UniFrac scores of 4, Taxy_pro vs Metaphyler using TAMPA on the CAMI dataset at the class rank. Note the differences in taxa predictions even though the tools have identical UniFrac scores.

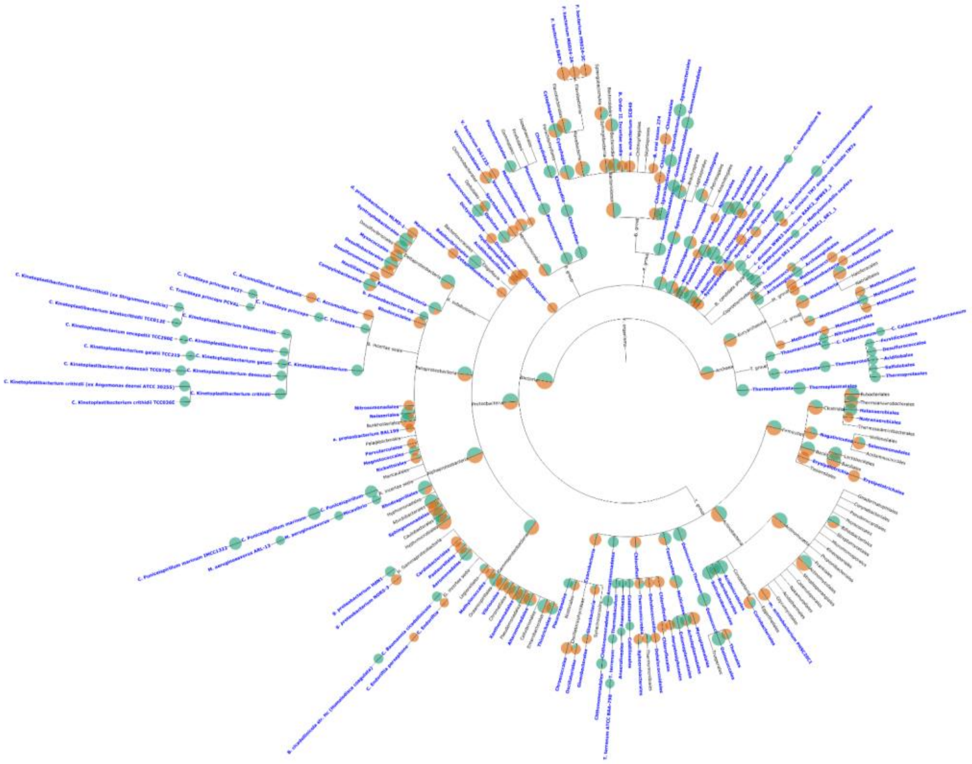

Figure S2: Visualization of the taxonomic profiles of tools with identical UniFrac scores of 4, Taxy_pro vs Metaphyler using TAMPA on the CAMI dataset at the order rank.

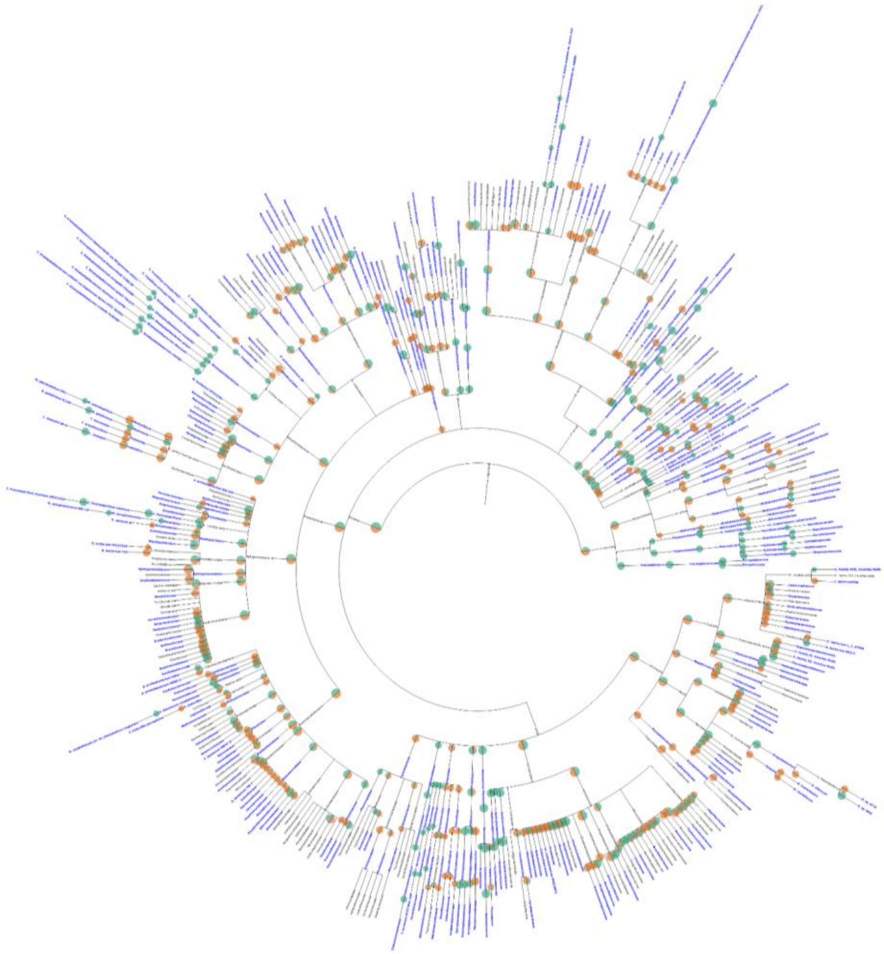

Figure S3: Visualization of the taxonomic profiles of tools with identical UniFrac scores of 4, Taxy_pro vs Metaphyler using TAMPA on the CAMI dataset at the family rank

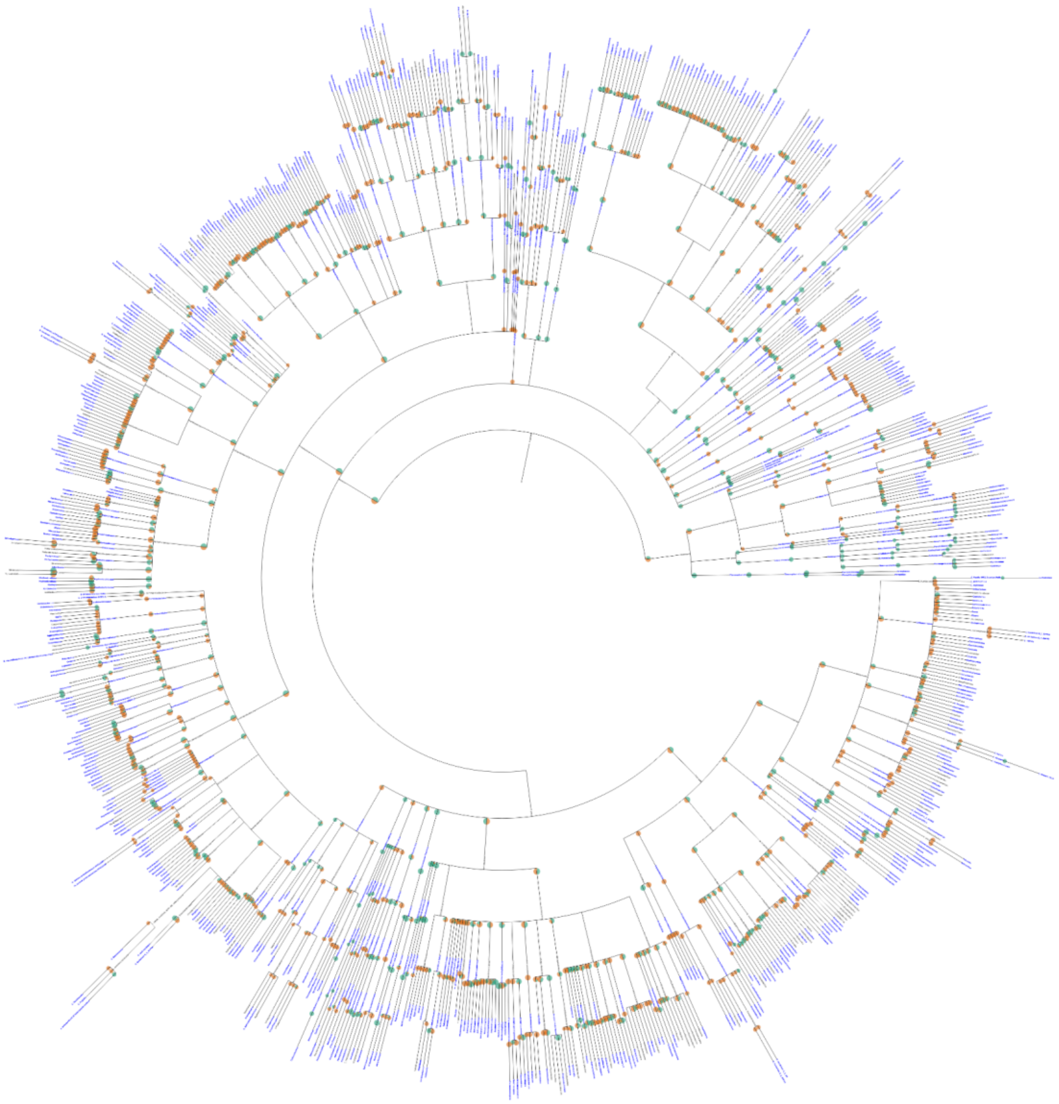

Figure S4: Visualization of the taxonomic profiles of tools with identical UniFrac scores of 4, Taxy_pro vs Metaphyler using TAMPA on the CAMI dataset at the genus rank

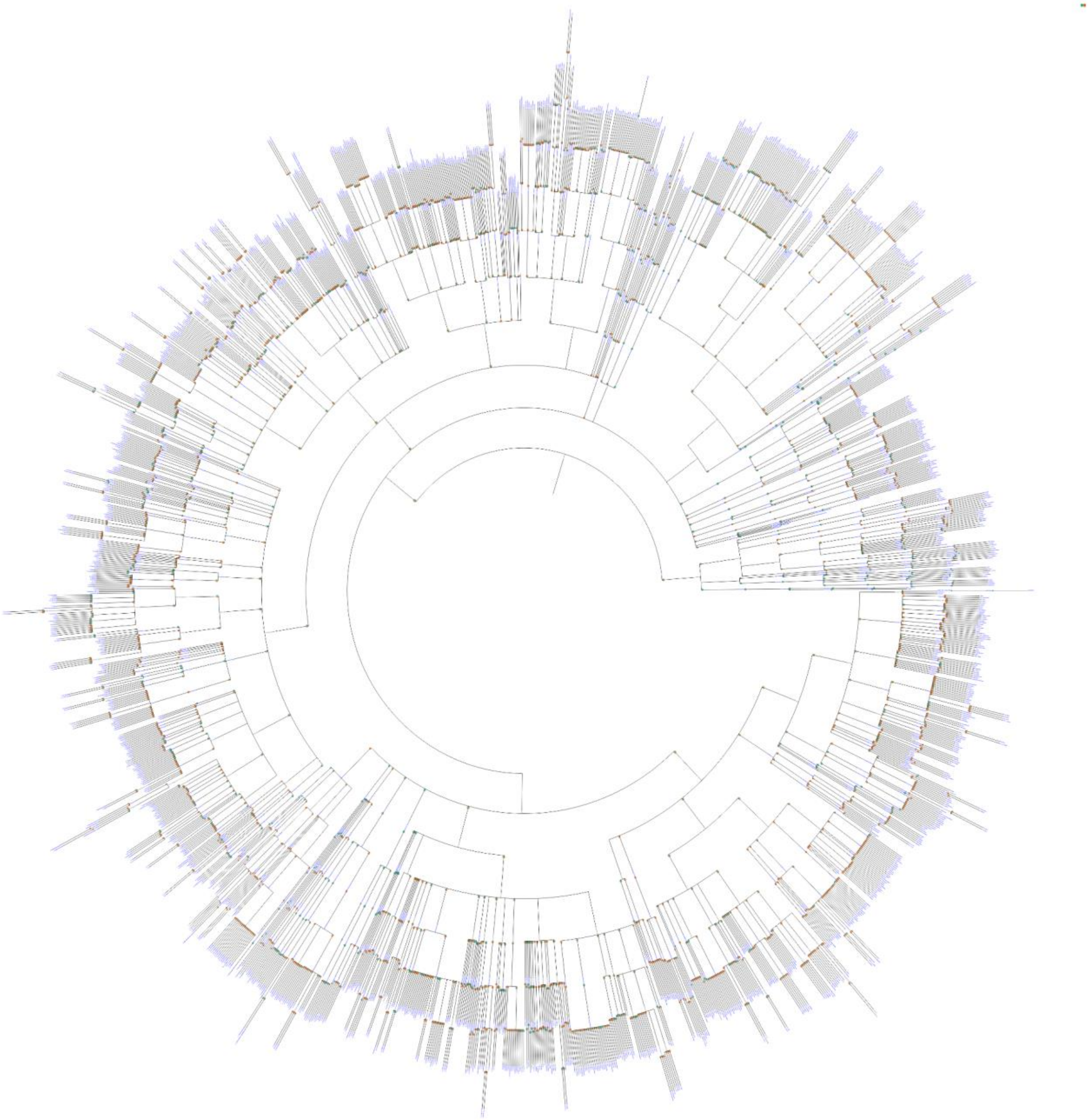

Figure S5: Visualization of the taxonomic profiles of tools with identical UniFrac scores of 4, Taxy_pro vs Metaphyler using TAMPA on the CAMI dataset at the species rank

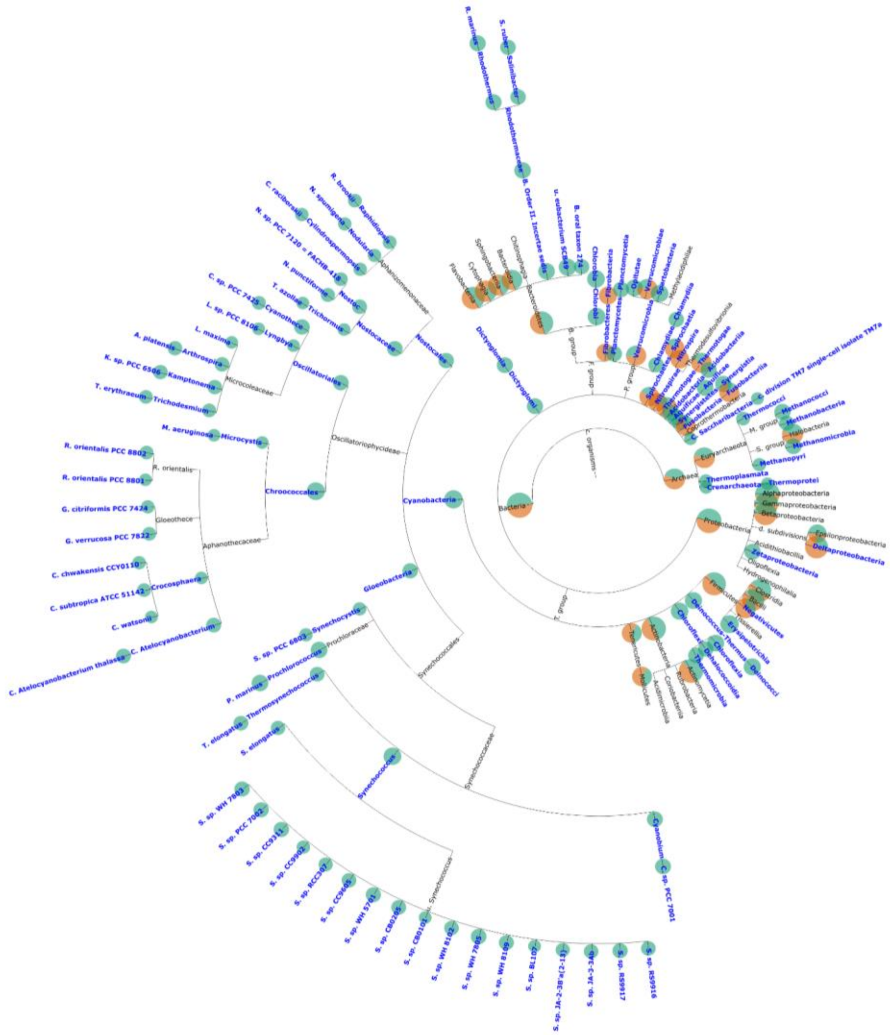

Figure S6: Visualization of the taxonomic profiles of a top performing CAMI tool, Metaphyler vs the ground truth using TAMPA on the CAMI dataset at the class level.

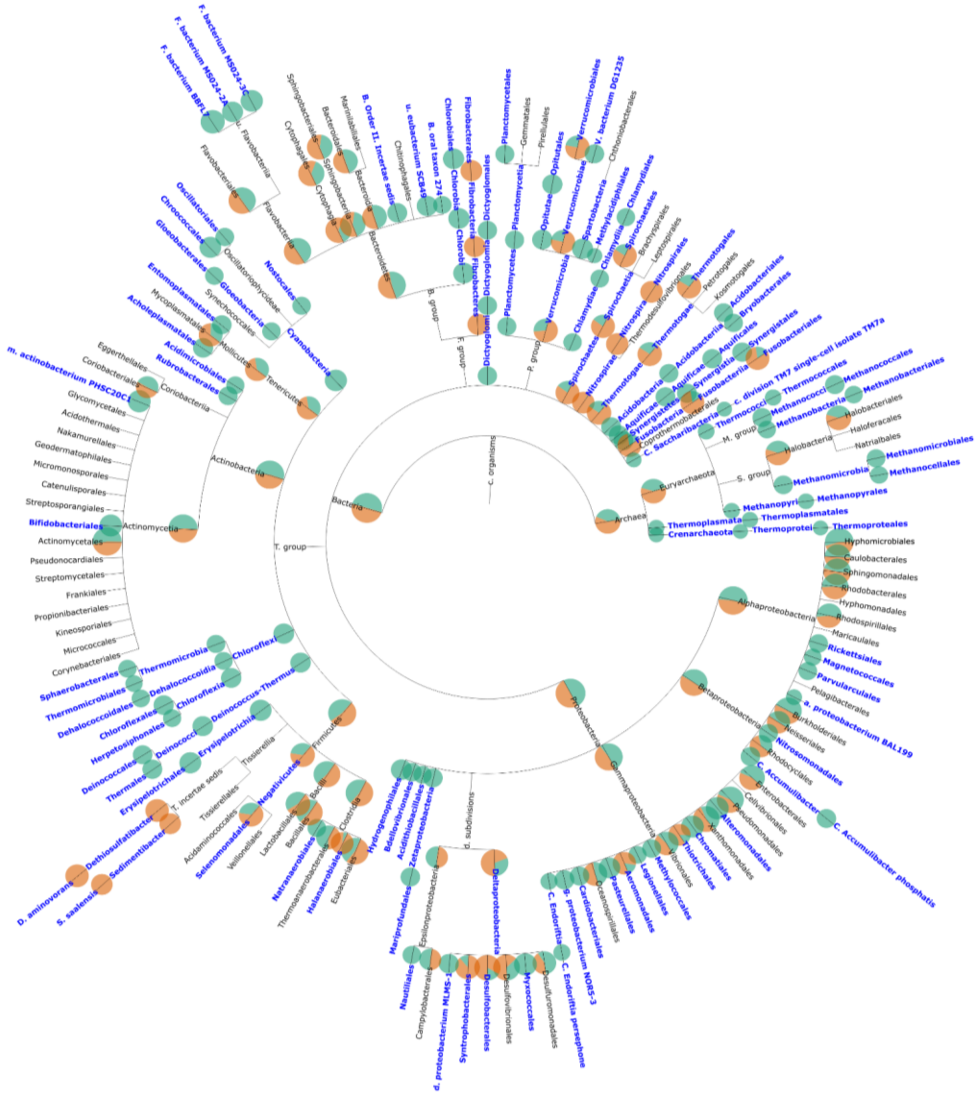

Figure S7: Visualization of the taxonomic profiles of a top performing CAMI tool, Metaphyler vs the ground truth using TAMPA on the CAMI dataset at the order level.

a)

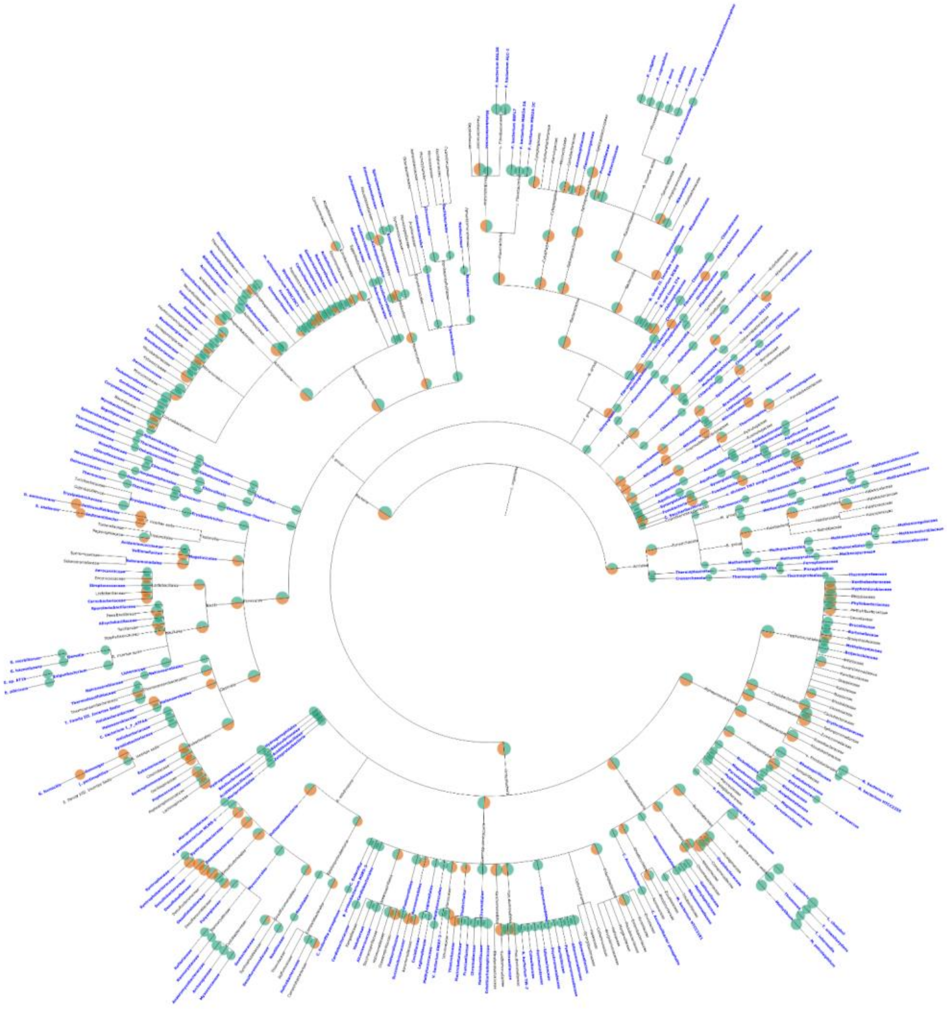

b)

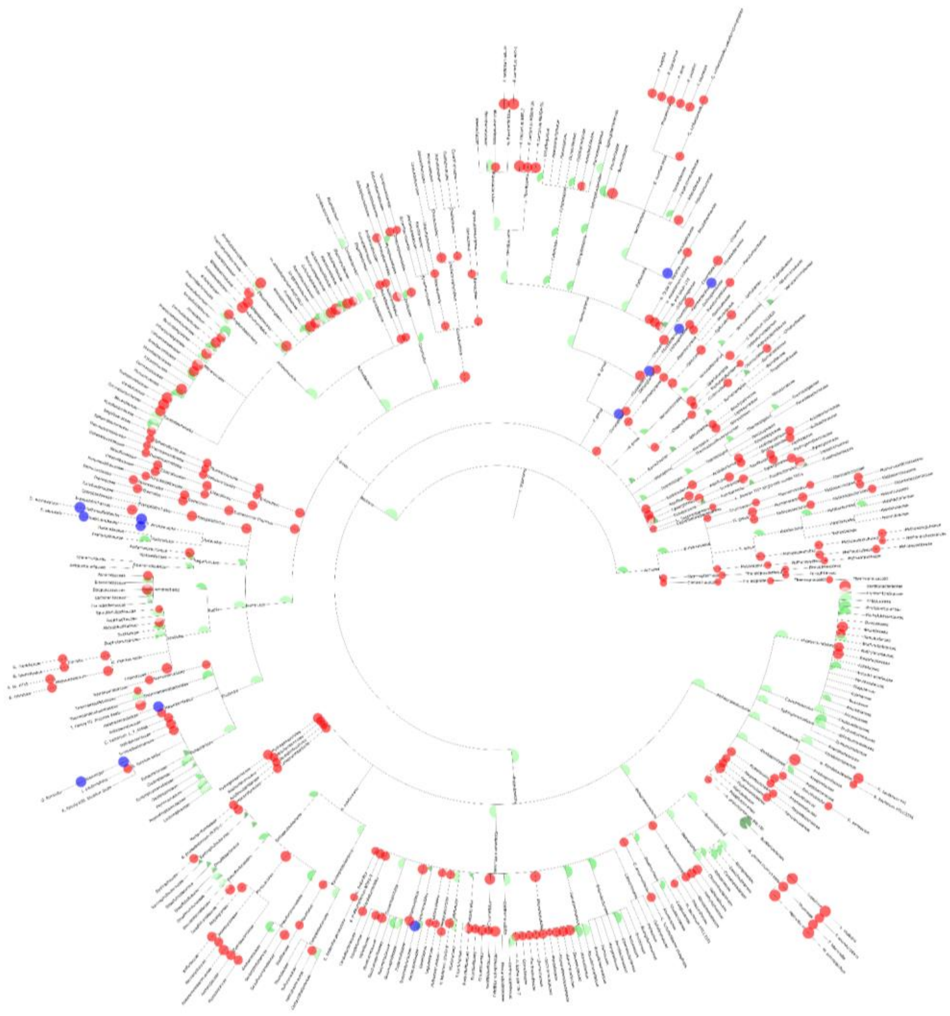

Figure S8: a) Visualization of the taxonomic profiles of a top performing CAMI tool, Metaphyler vs the ground truth using TAMPA on the CAMI dataset at the family level b) With contrast mode

a)

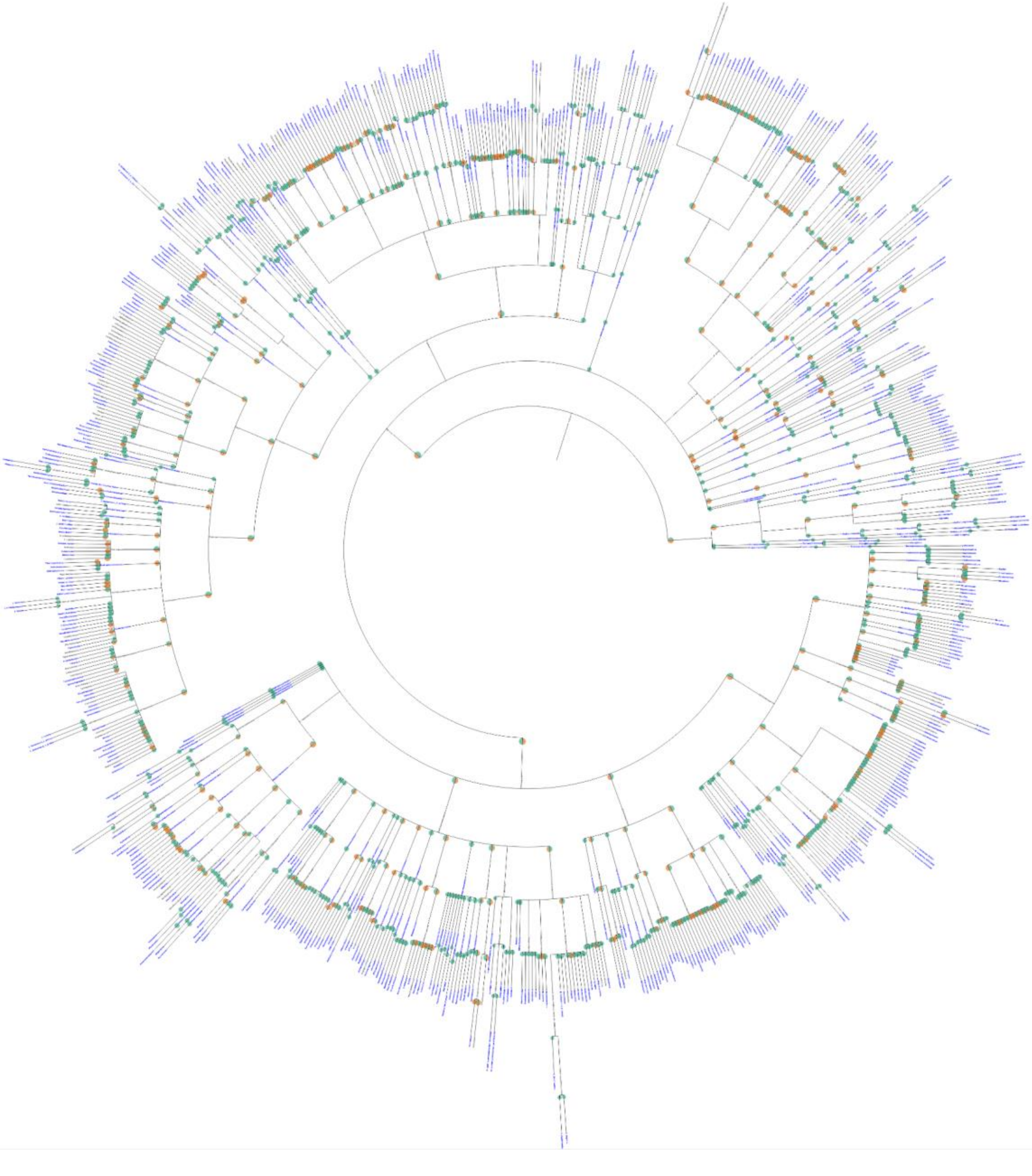

b)

a)

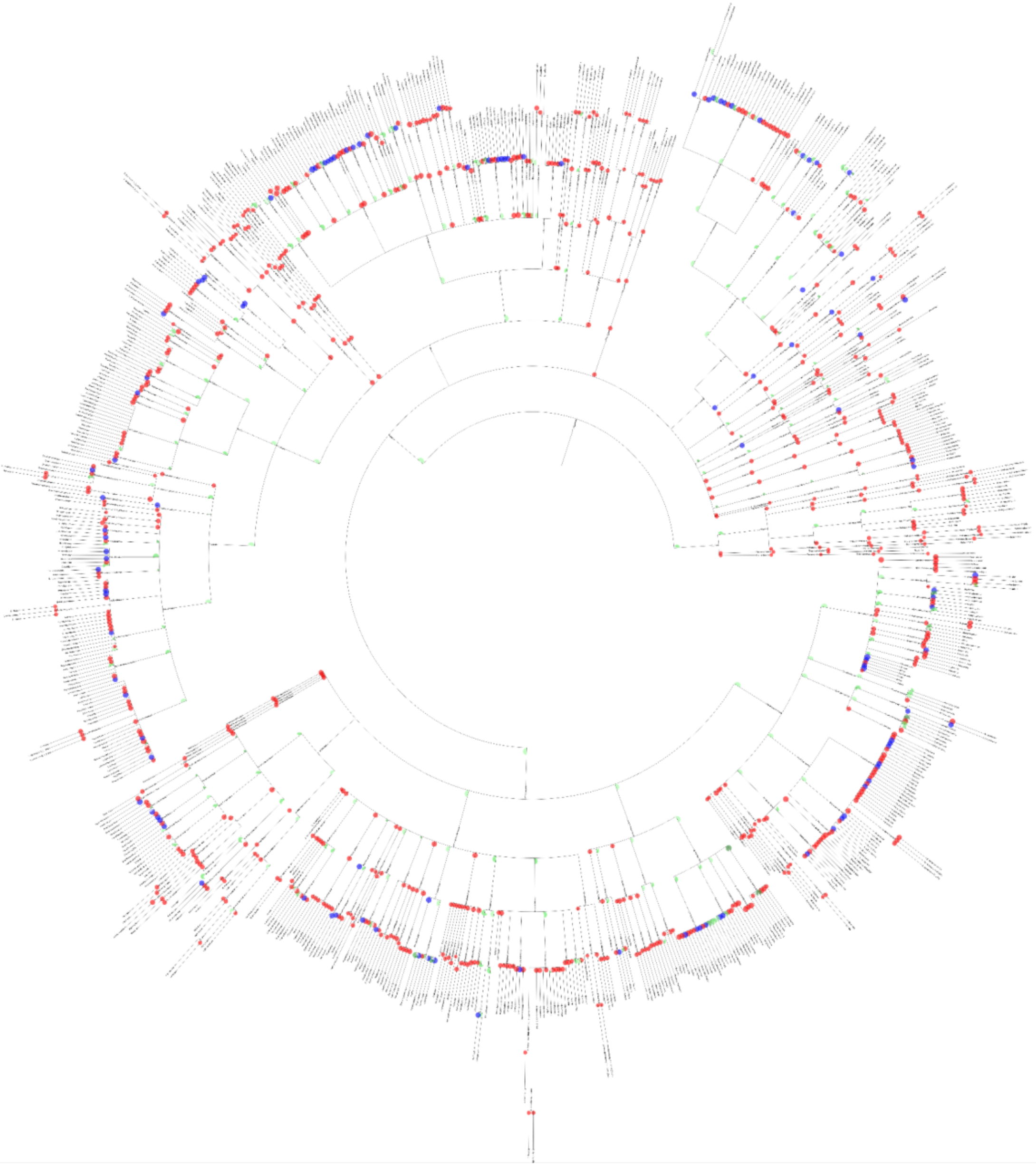

Figure S9: a) Visualization of the taxonomic profiles of a top performing CAMI tool, Metaphyler vs the ground truth using TAMPA on the CAMI dataset at the genus level b) With contrast mode.

a)

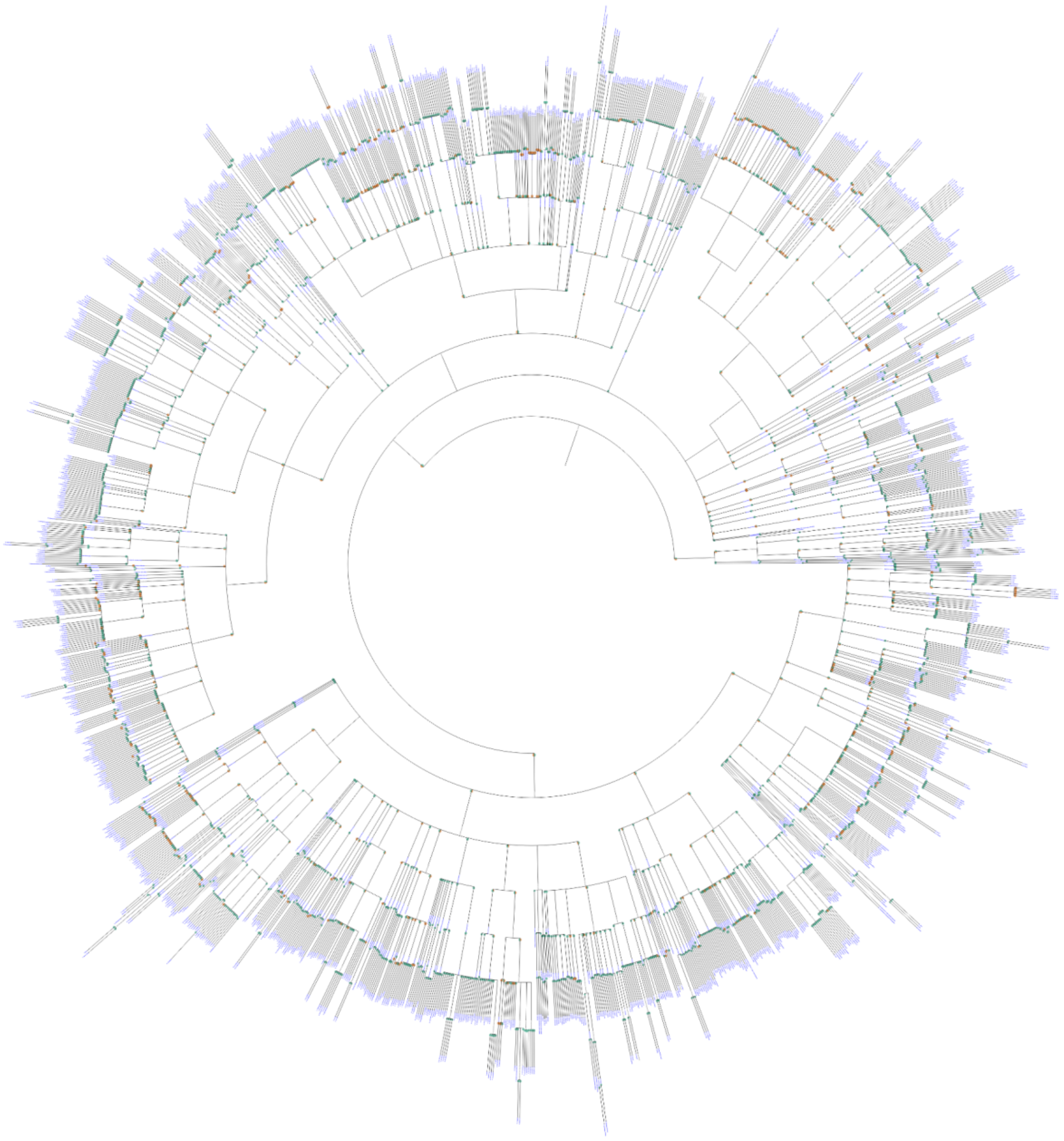

b)

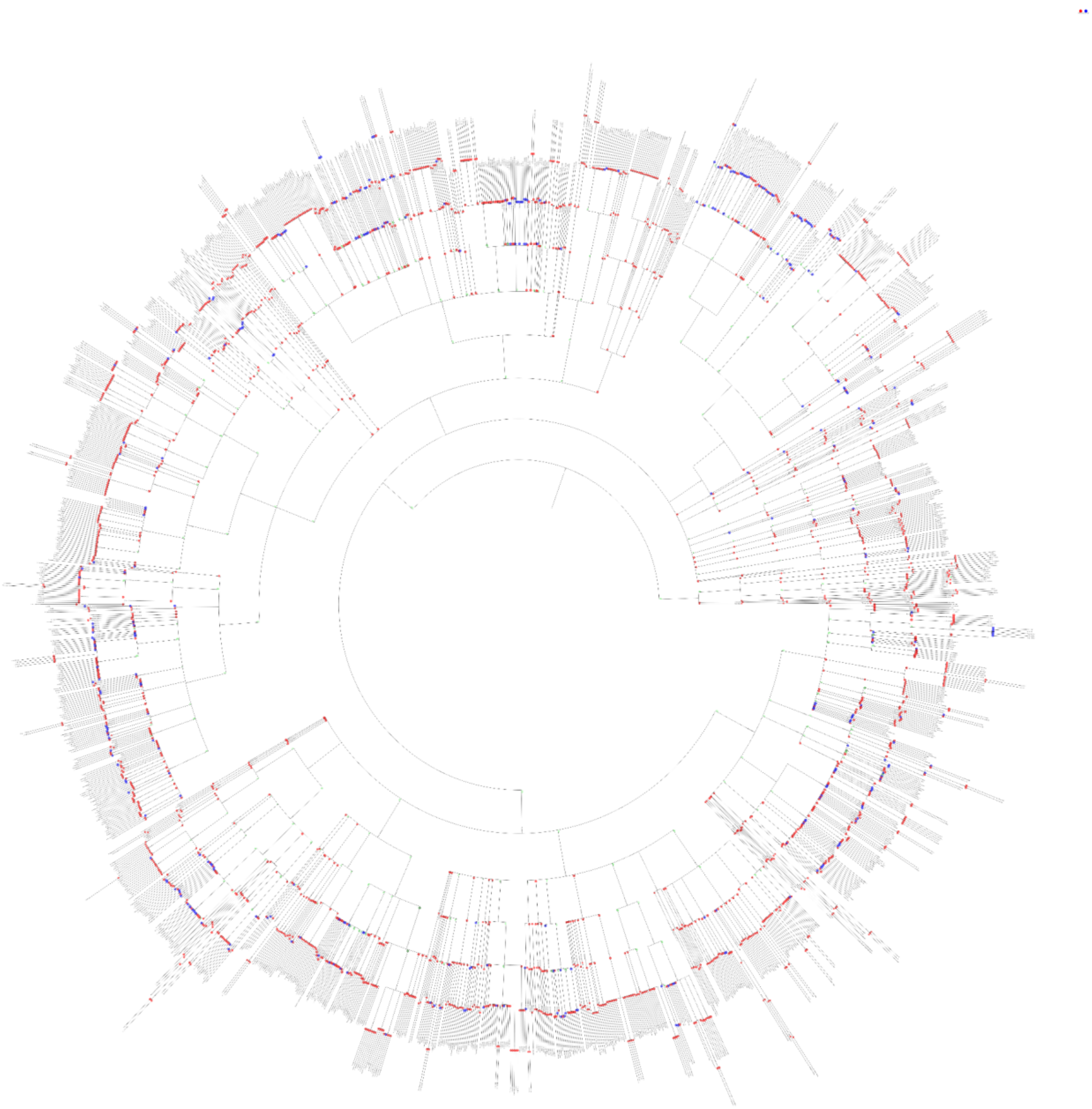

Figure S10: Visualization of the taxonomic profiles of a top performing CAMI tool, Metaphyler vs the ground truth using TAMPA on the CAMI dataset at the species level b) With contrast mode.

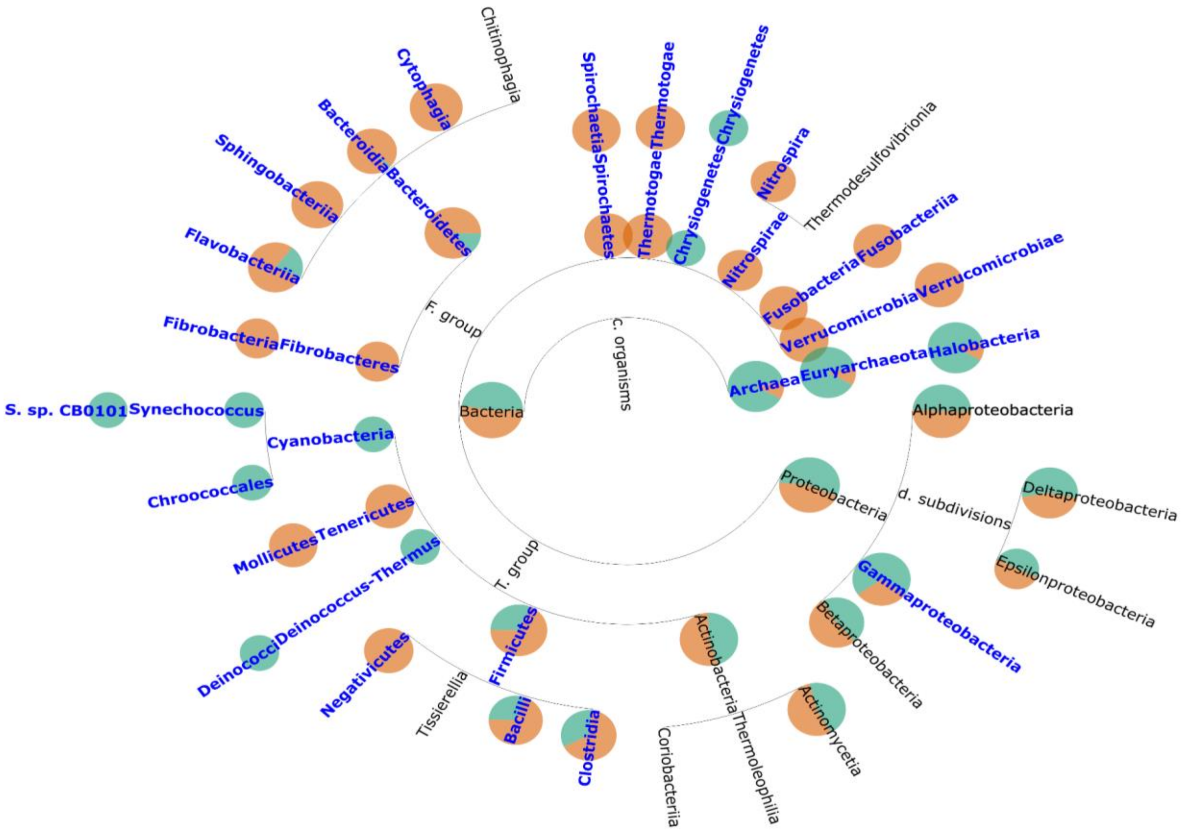

Figure S11: Visualization of the taxonomic profiles of the lowest performing tool, mOTU vs the ground truth using TAMPA on the CAMI dataset at the class level.

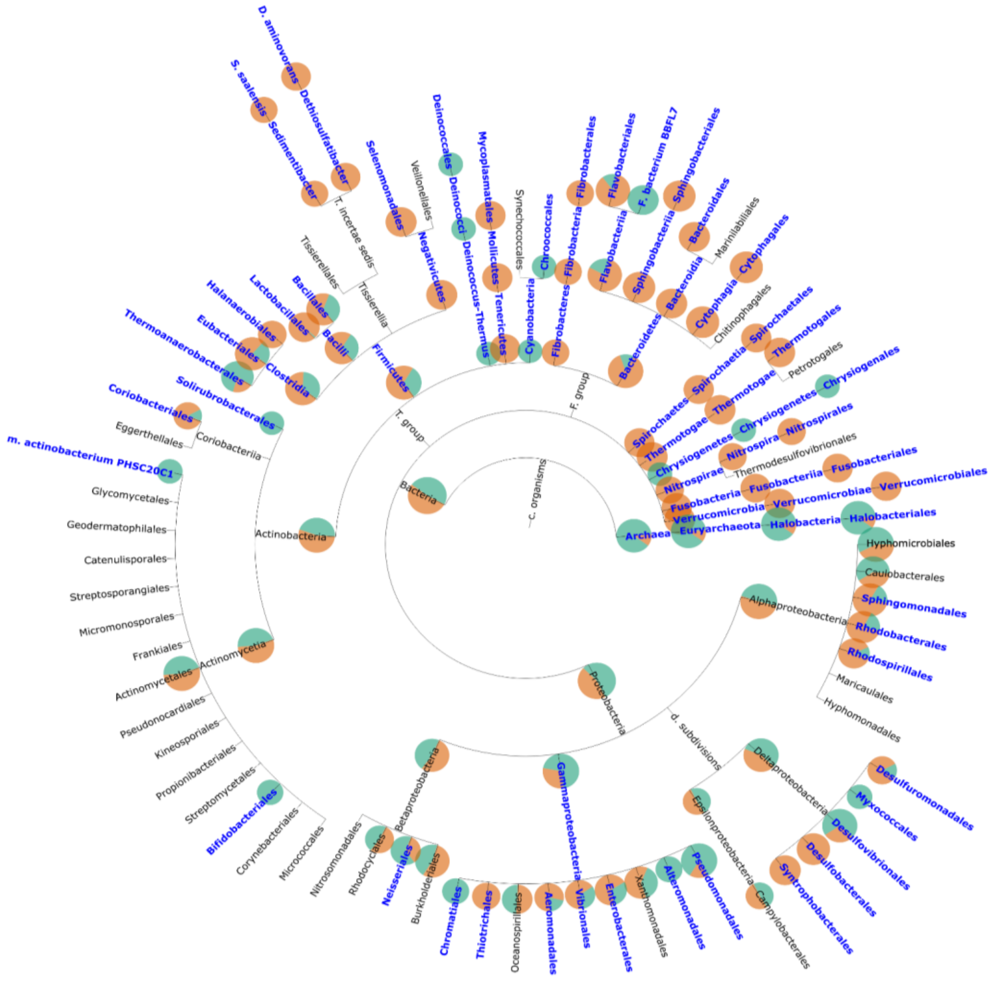

Figure S12: Visualization of the taxonomic profiles of the lowest performing tool, mOTU vs the ground truth using TAMPA on the CAMI dataset at the order level.

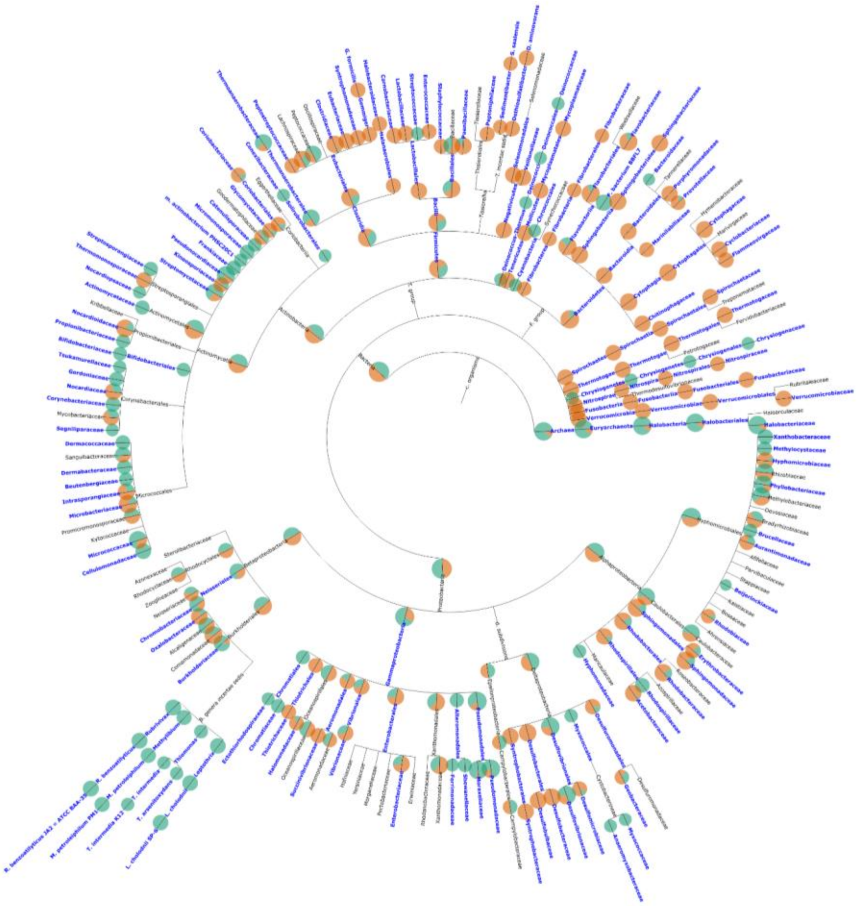

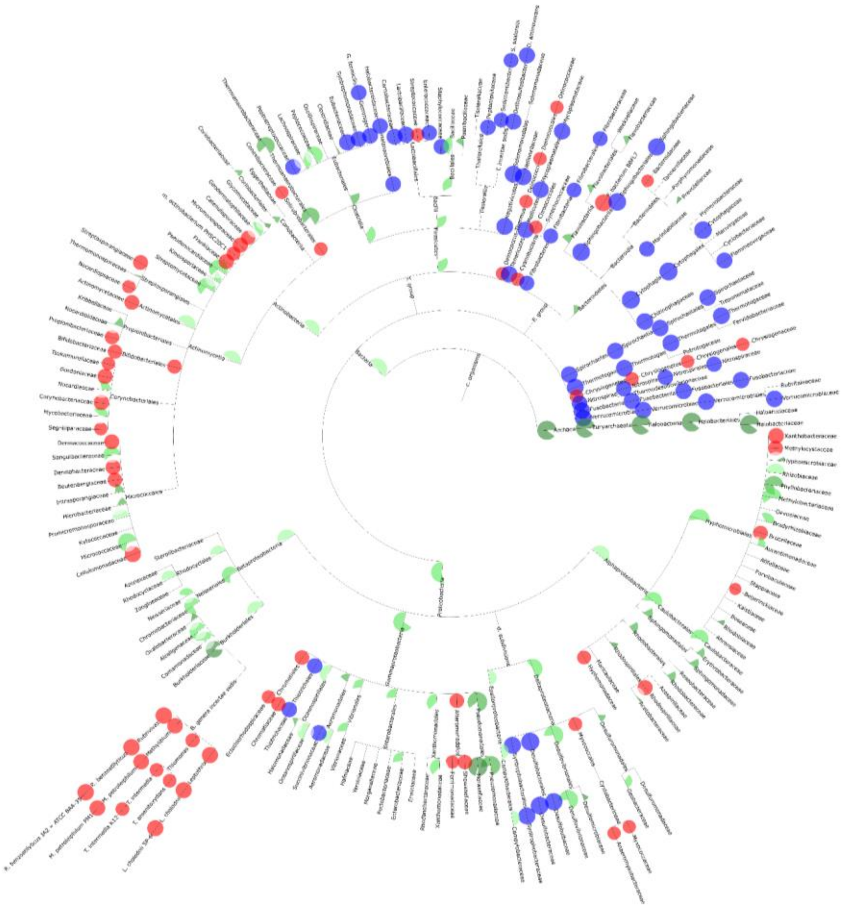

Figure S13: a) Visualization of the taxonomic profiles of the lowest performing tool, mOTU vs the ground truth using TAMPA on the CAMI dataset at the family level b) With contrast mode.

a)

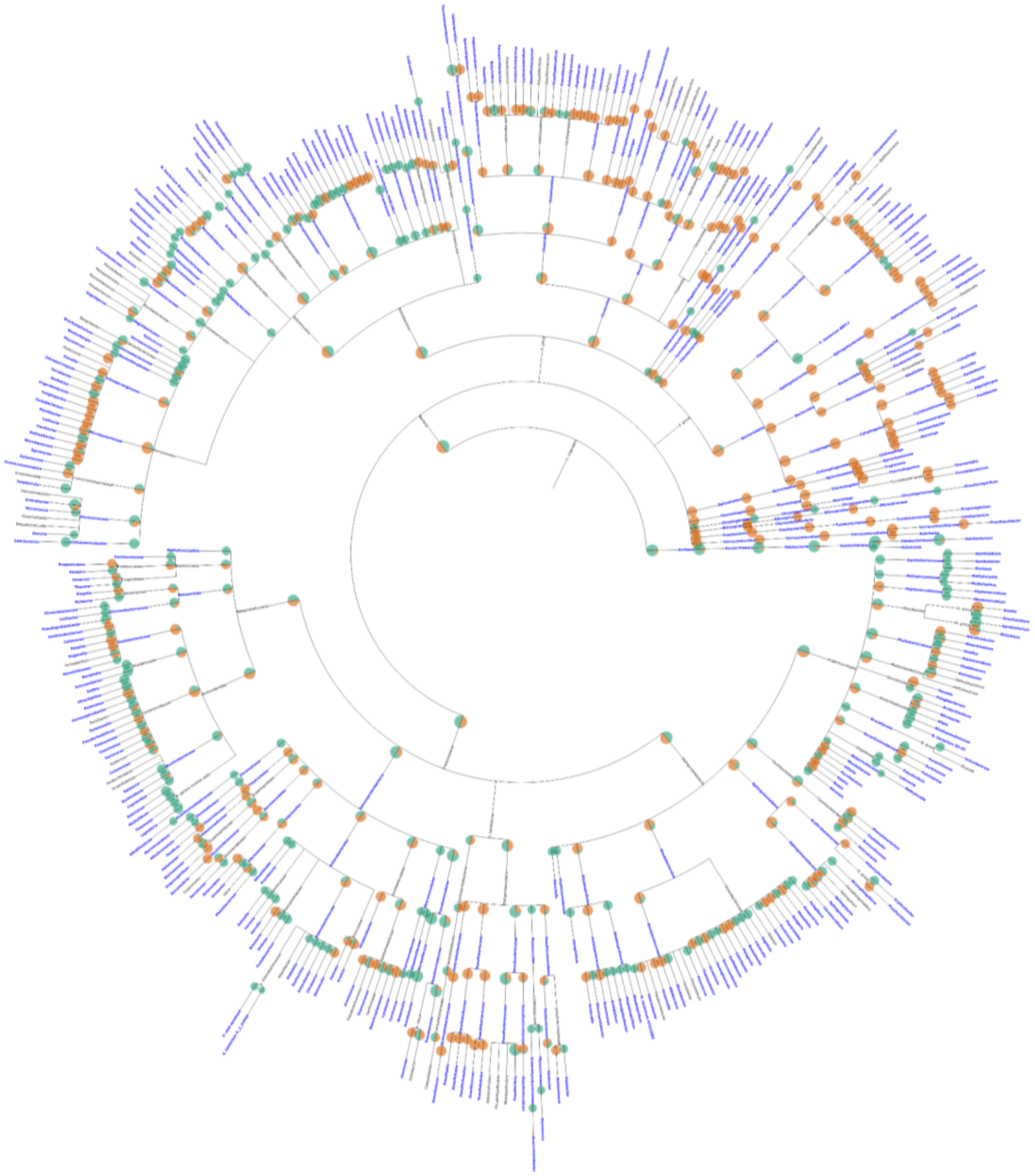

b)

a)

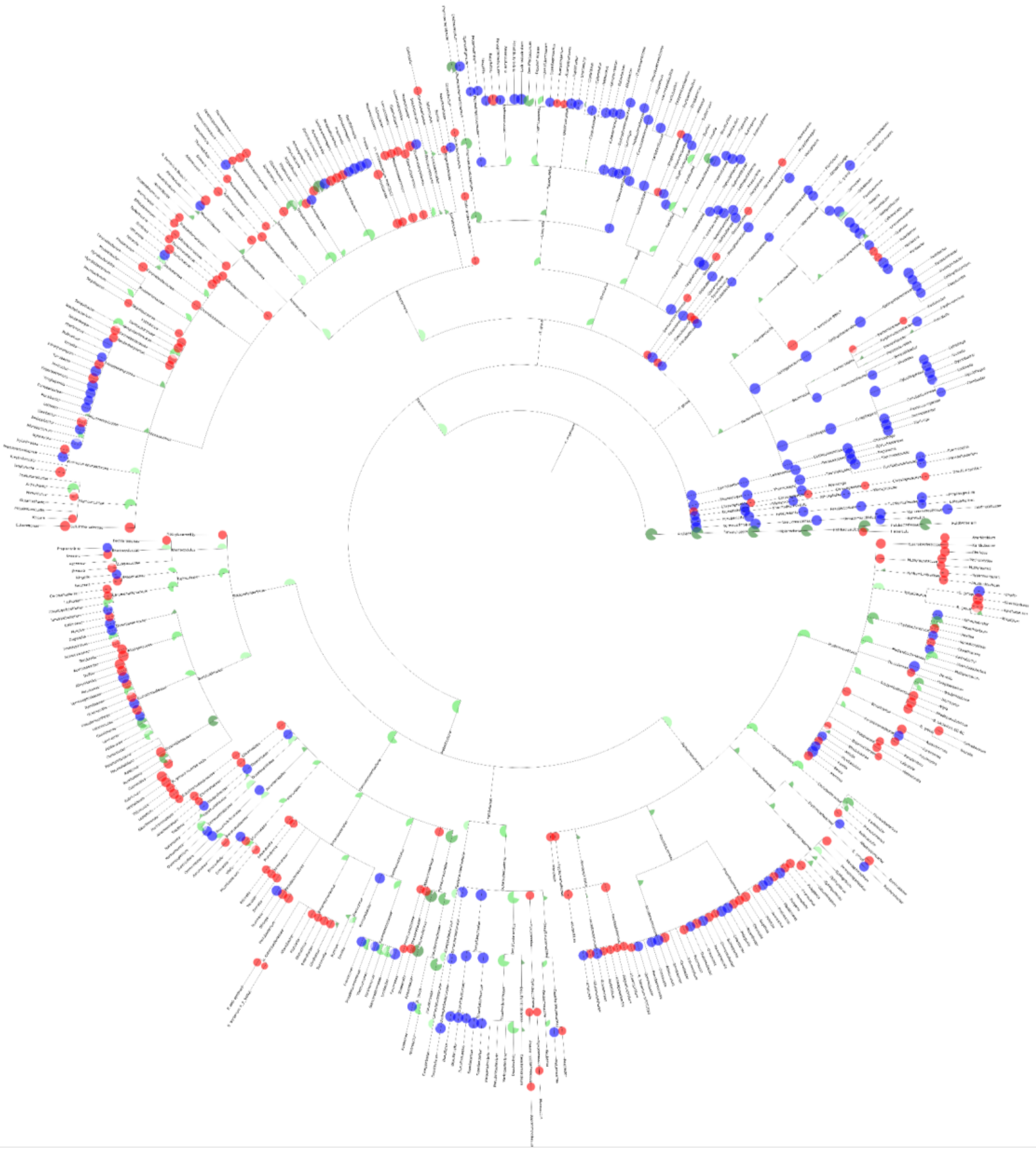

Figure S14: a) Visualization of the taxonomic profiles of the lowest performing tool, mOTU vs the ground truth using TAMPA on the CAMI dataset at the genus level b) With contrast mode.

a)

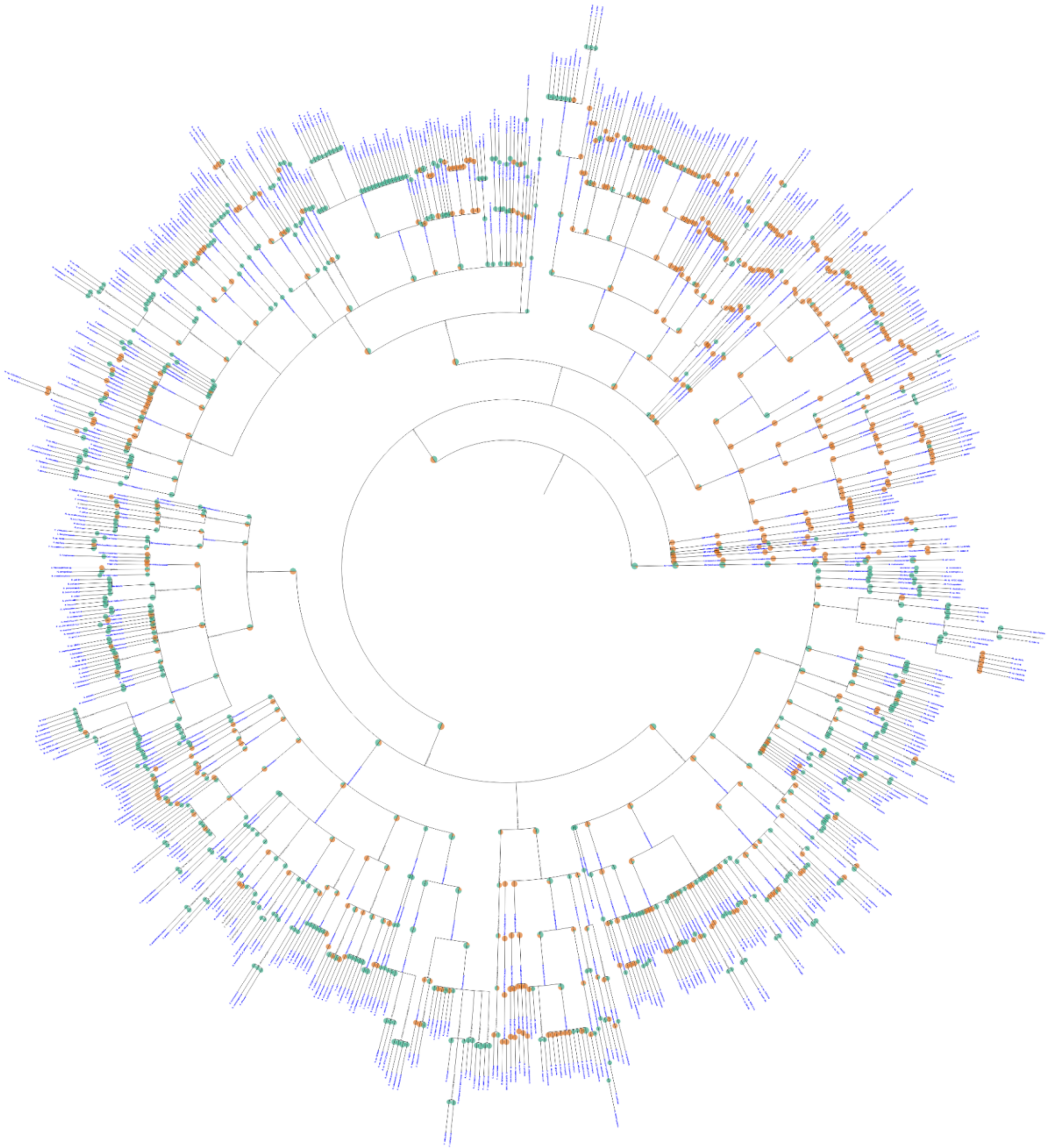

b)

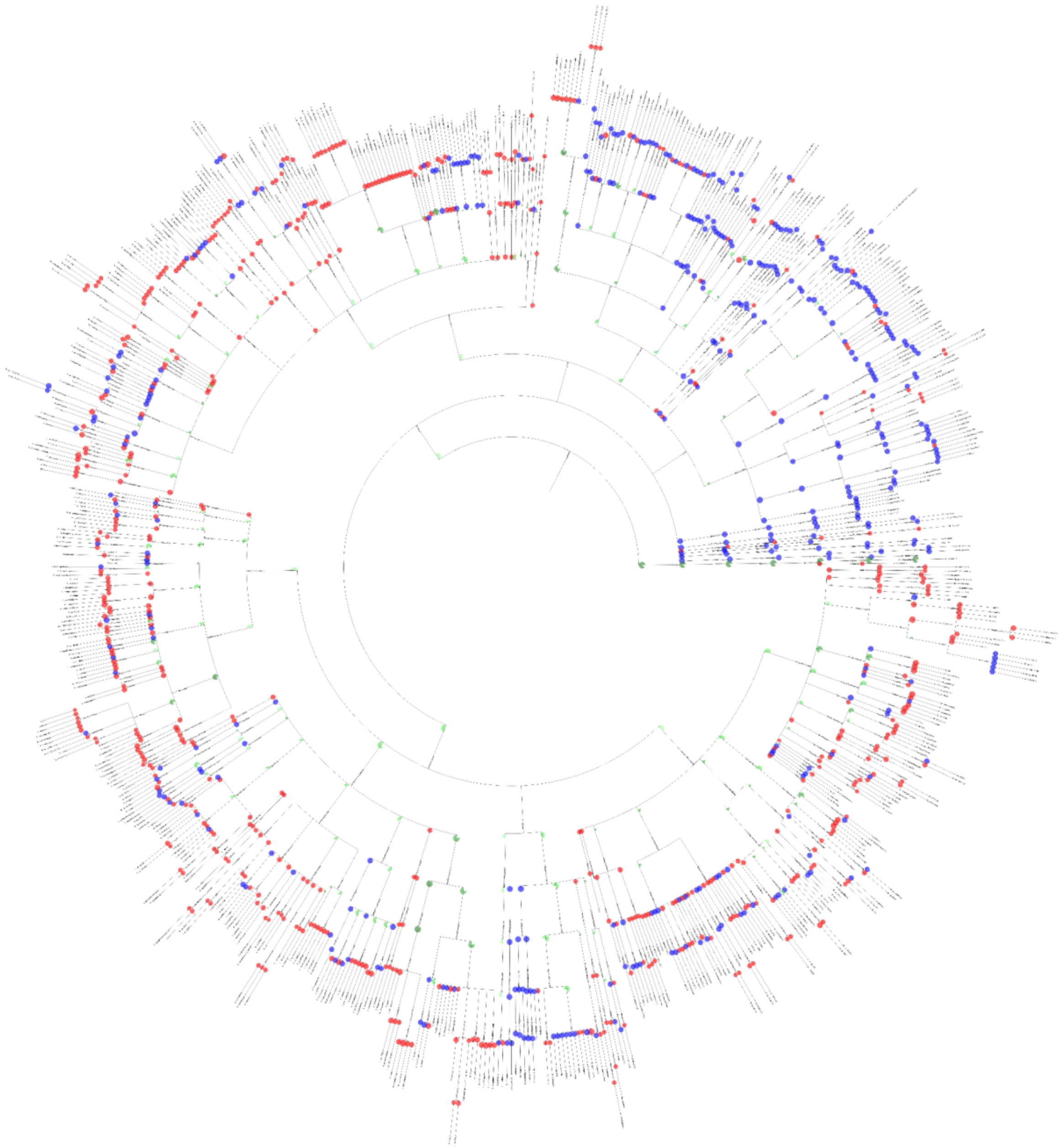

Figure S15: Visualization of the taxonomic profiles of the lowest performing tool, mOTU vs the ground truth using TAMPA on the CAMI dataset at the species level b) With contrast mode.

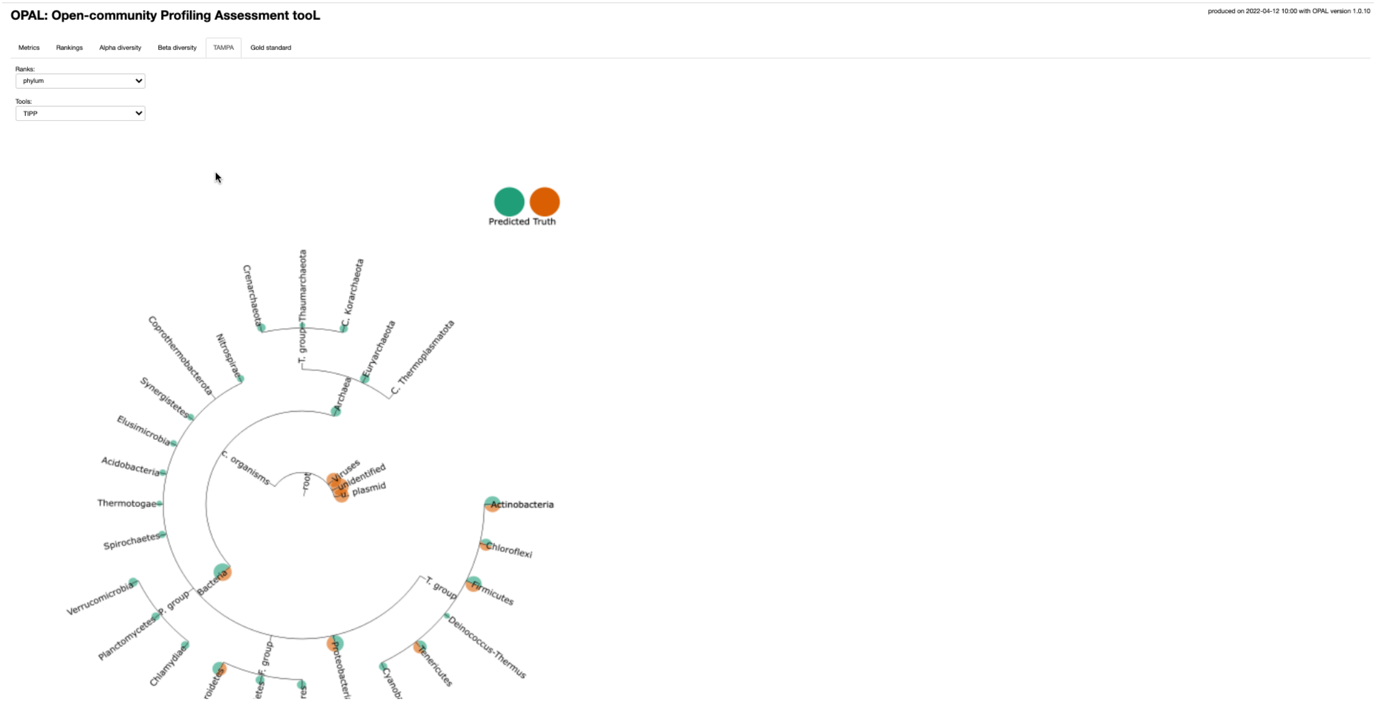

Figure S16: Incorporation of TAMPA into OPAL

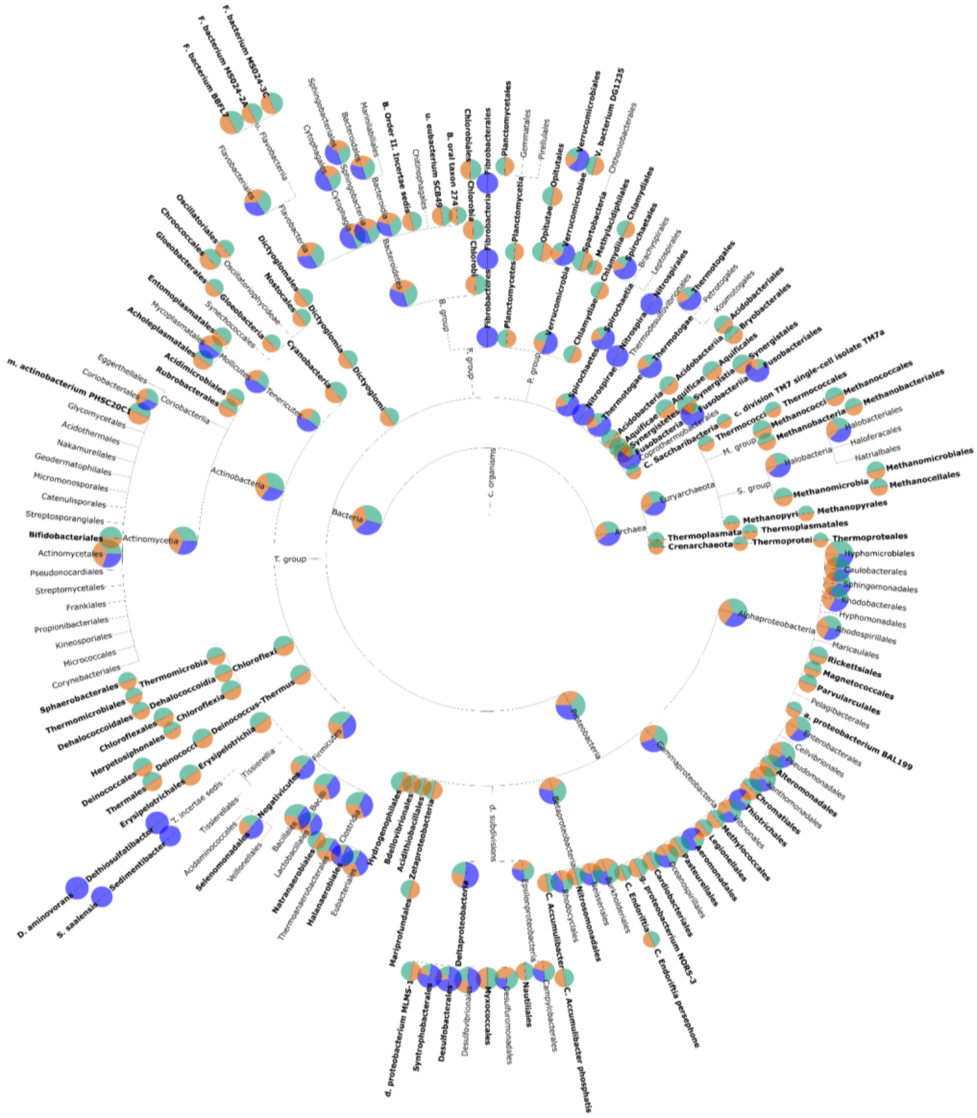

Figure S17: Visualization of the taxonomic profiles of three tools: Taxy_pro (green) vs Metaphyler (orange) vs mOUT (blue). using TAMPA on the CAMI dataset at the class rank.

figure 1,2

Click here to access/download;Figure;figure1and2.pdf

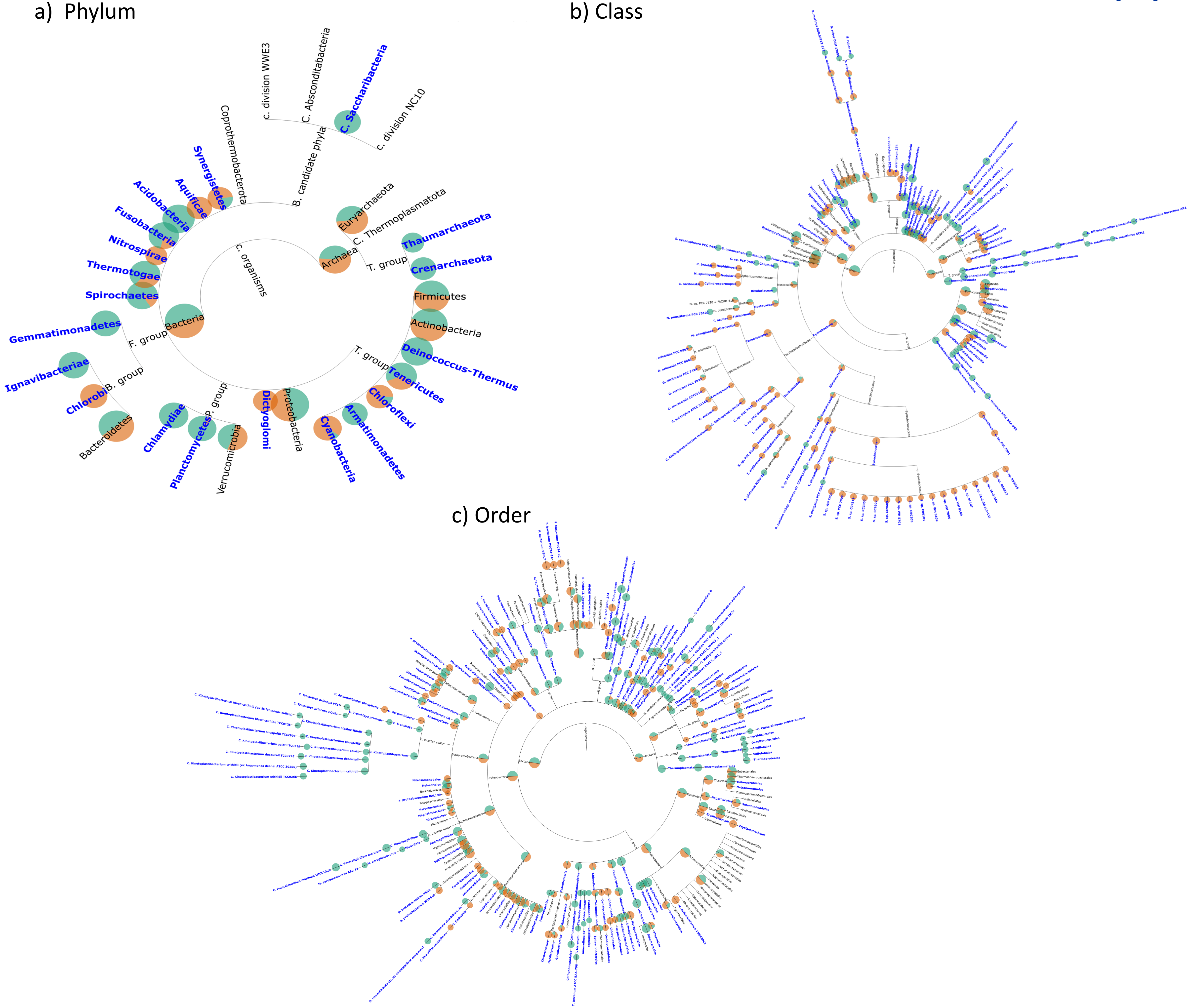

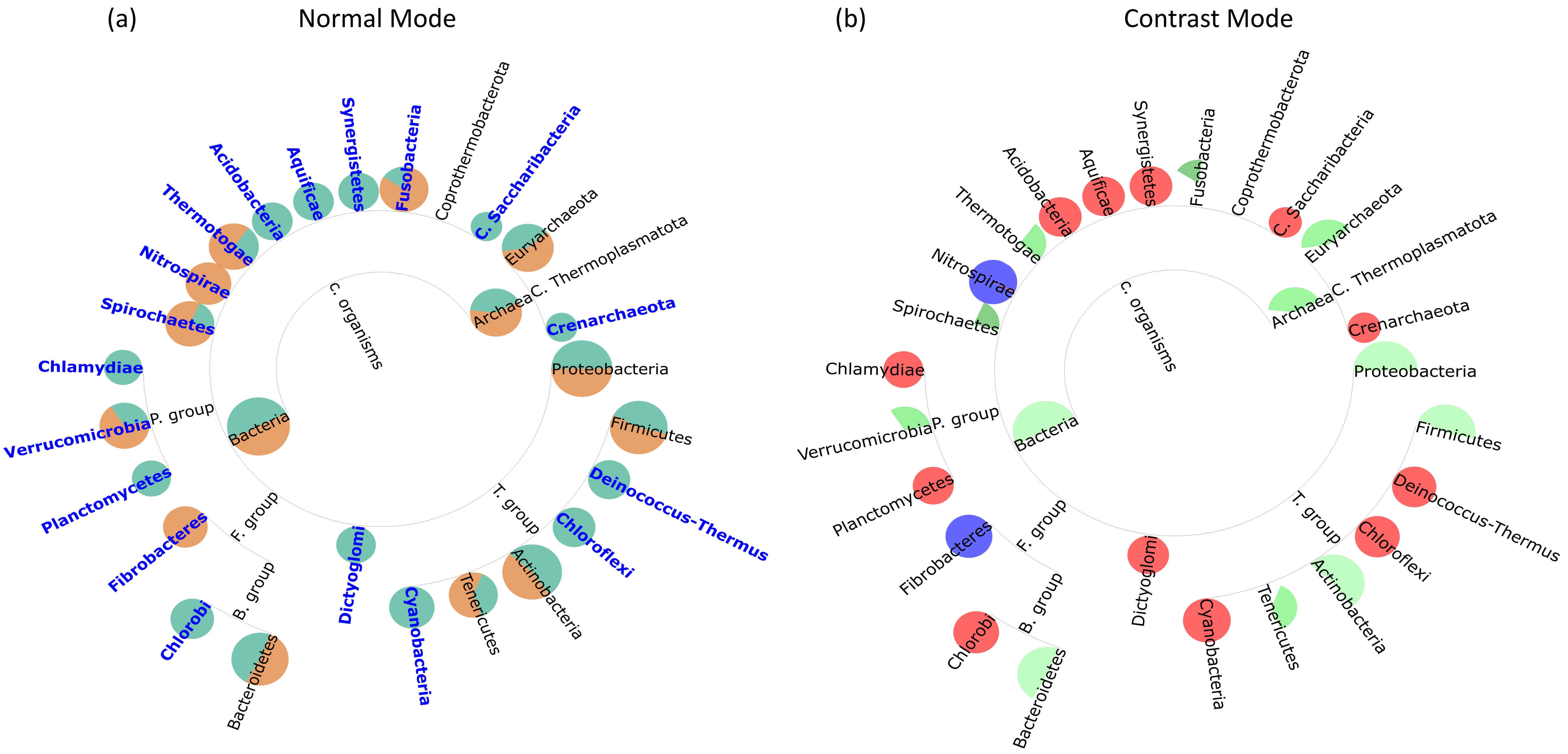
(a)
Normal Mode
(b)
Contrast Mode
c. organisms
Bacteria
Archaea
P. group
F. group
B. group
T. group
Acidobacteria
Aquificae
Synergistetes
Fusobacteria
Coprothermobacterota
C. Saccharibacteria
Euryarchaeota
C. Thermoplasmatota
Crenarchaeota
Proteobacteria
Firmicutes
Deinococcus-Thermus
Chloroflexi
Actinobacteria
Tenericutes
Cyanobacteria
Dictyoglomi
Bacteroidetes
Chlorobi
Fibrobacteres
Planctomycetes
Verrucomicrobia
Chlamydiae
Spirochaetes
Nitrospirae
Thermotogae

figure 3

Click here to access/download;Figure;Figure3_final.pdf

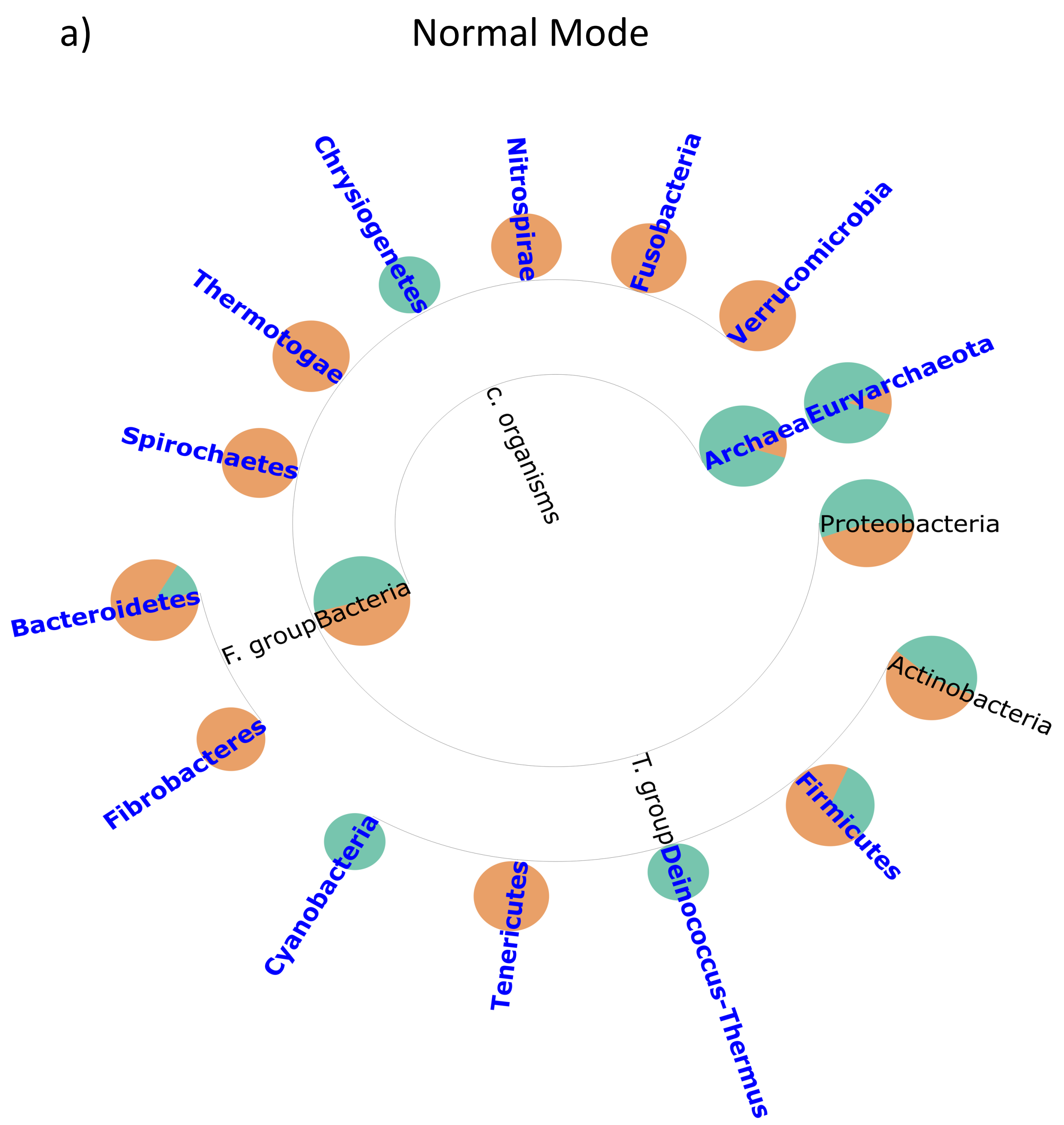

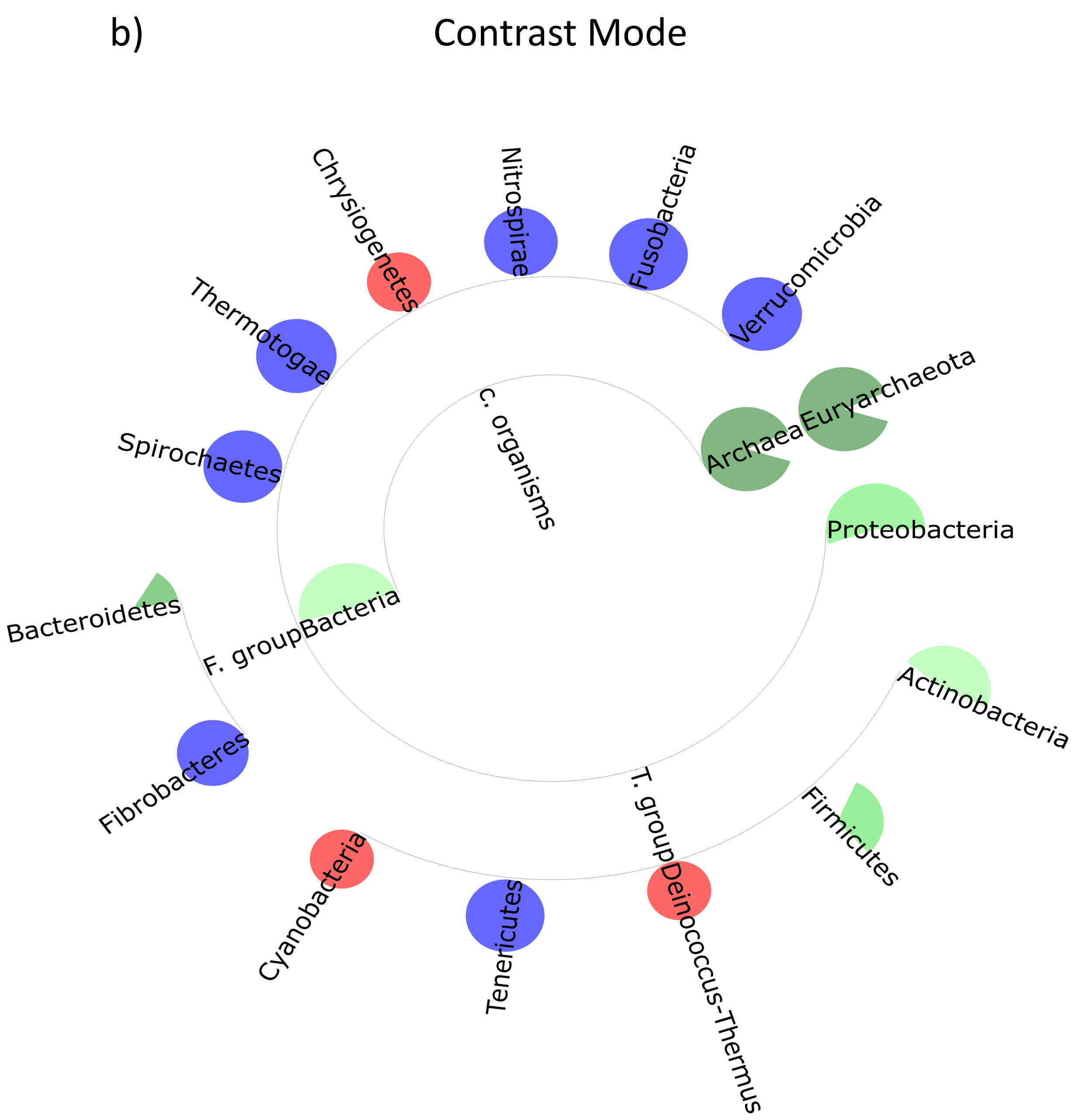

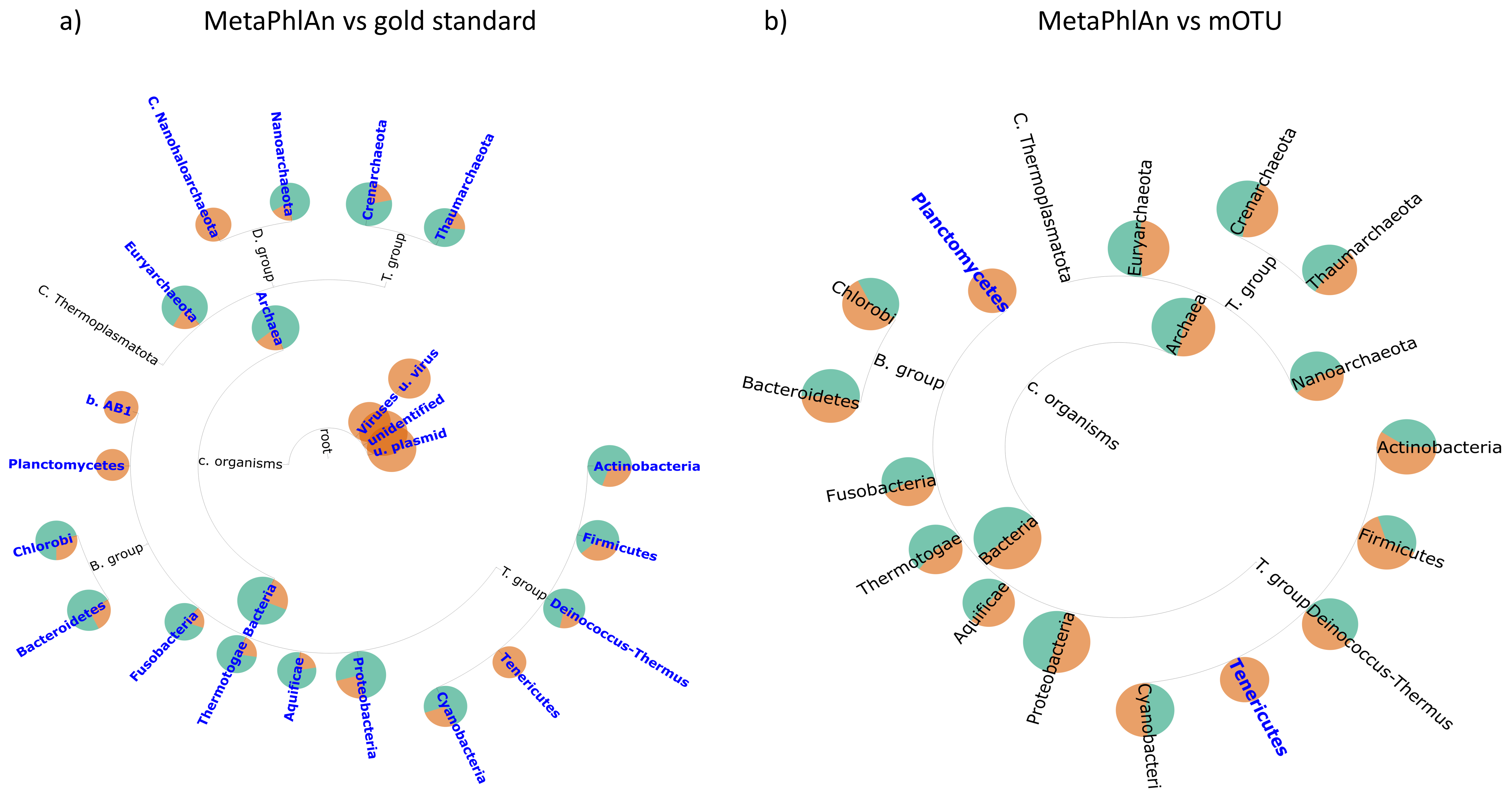
a)
MetaPhlAn vs gold standard
C. Nanohaloarchaeota
Nanoarchaeota
Crenarchaeota
Thaumarchaeota
D. group
T. group
Euryarchaeota
C. Thermoplasmatota
Archaea
u. virus
Viruses
unidentified
u. plasmid
root
b. AB1
Planctomycetes
c. organisms
Actinobacteria
Chlorobi
B. group
Firmicutes
T. group
Deinococcus-Thermus
Bacteroidetes
Fusobacteria
Thermotogae
Bacteria
Aquificae
Proteobacteria
Cyanobacteria
Tenericutes
b)
MetaPhlAn vs mOTU
C. Thermoplasmatota
Crenarchaeota
Planctomycetes
Euryarchaeota
Thaumarchaeota
Chlorobi
T. group
Archaea
B. group
Nanoarchaeota
Bacteroidetes
c. organisms
Actinobacteria
Fusobacteria
Firmicutes
Thermotogae
Bacteria
Aquificae
T. group
Deinococcus-Thermus
Proteobacteria
Cyanobacteria
Tenericutes

Personal Cover
Click here to access/download;Personal Cover;TAMPA_response to reviewers.pdf

Reviewer 1 Comment 1:
The tool should be easy to install, I would suggest to add it to bioconda, which allow the installation of all dependencies (see CODE EVALUATION below).
CODE EVALUATION --

I downloaded with conda (`conda install -c vsarwal tampa`) and have the following error:

```
$ profile_to_plot
Traceback (most recent call last):
  File "/miniconda3/bin/profile_to_plot", line 33, in <module>
    sys.exit(load_entry_point('CAMIViz==0.1.0', 'console_scripts', 'profile_to_plot')())
  File "/miniconda3/bin/profile_to_plot", line 25, in importlib_load_entry_point
    return next(matches).load()
  File "/miniconda3/lib/python3.7/site-packages/importlib_metadata/__init__.py", line 167, in load
    module = import_module(match.group('module'))
  File "/miniconda3/lib/python3.7/importlib/__init__.py", line 127, in import_module
    return _bootstrap._gcd_import(name[level:], package, level)
  File "<frozen importlib._bootstrap>", line 1006, in _gcd_import
  File "<frozen importlib._bootstrap>", line 983, in _find_and_load
  File "<frozen importlib._bootstrap>", line 967, in _find_and_load_unlocked
  File "<frozen importlib._bootstrap>", line 677, in _load_unlocked
  File "<frozen importlib._bootstrap_external>", line 728, in exec_module
  File "<frozen importlib._bootstrap>", line 219, in _call_with_frames_removed
  File "/miniconda3/lib/python3.7/site-packages/src/profile_to_plot.py", line 2, in <module>
    from ProfilesLayout import ProfilesLayout
ModuleNotFoundError: No module named 'ProfilesLayout'
```

I have the same error if I follow the installation in https://github.com/dkoslicki/TAMPA.

In order to make the tool easier to use I would suggest to:
- change the name of the executable from `profile_to_plot` to `tampa`
- you could add the package to bioconda to have all the dependencies installed
- change the version to 1.0.0 for the release

Response: We thank the reviewer for pointing out these errors. We have added the package on bioconda with dependencies installed, changed the name of the executable from profile_to_plot to tampa, and changed the version to be 1.0.0 for the release.

Reviewer 1 Comment 2:
TAMPA has limited applicability when comparing to Ground Truth. The tool allow to compare two different profiles, but there are conceptually two different evaluations: 1) compare two different profiles, and 2) compare a profile to the Ground Truth. The current implementation works good for the first evaluation, but I can see it can be improved for the second. For example, it would be interesting to visualise as a red circle false positive taxa (for example in Figure 2 Cyanobacteria), in blue false negatives (Nitrospirae in Figure 2) and in a gradient of white to green when the taxa expected from the Ground truth is measured: in white if the relative abundance is the same and in green if the relative abundance is different between expected and measured (with the colour intensity proportional to the error). In this way all

correct taxa will be white, and looking at the tree colors we could understand the issue: red (false positive), blue (false negative), green (measure the correct taxa, but with wrong relative abundance). In this way, it would be much easier to spot issues in the tree, which is one of the main applications of TAMPA within the CAMI benchmark. This would be even more beneficial when there are large trees (example Figure S3), to quickly identify which part of the tree is the most problematic.

Response: We thank the reviewer for the great ideas. We have created a new mode for TAMPA called "contrast", for better visualization of the comparison of the profile output with the ground truth, with a color spectrum. We have updated figure 2,3 with plots using the contrast mode, as well as added new text and images to the results and supplementary section (highlighted in red in the updated manuscript).

Reviewer 1 Comment 3:
In the main text there are references to Figure 1b and 1c, but the figures are labeled 2 and 3 instead.

Response: We thank the reviewer for pointing this out. We have changed the references to figure 2 and 3.

Reviewer 1 Comment 4:
The paper present a tool, as such I would expect a more detailed description of the tool implementation and options in the methods. This would be helpful for people to use the tool.

Response: We thank the reviewer for their comment. We have created a new subsection in the Methods section, called Implementation and Features (highlighted in red in the updated manuscript), outlining how the tool has been implemented, and the various options available to the user.

Reviewer 2 Comment 1:
It would be interesting to see (if possible) the comparison of three (or more) taxonomic profiles at the same time. The evaluations shown are always binary, but in a real-case scenario where a user would like to evaluate 3 or 4 different taxonomic profiling tools on his community, it would be great to be able to do it.

Response: We thank the reviewer for their comment. We have modified the tool so that 3 tools can be compared at once. Visualization of 4 and more tools is out of scope of this paper, we recommend that users perform multiple comparisons using TAMPA for improved visibility. We have added new figures and text to the results section (highlighted in red in the updated manuscript).

Reviewer 2 Comment 2:
Other than the evaluation on the agreement between two (or more) taxonomic profiling tools, it is not clear how TAMPA can drive improvement over biologically-relevant question. Although it is clear, as the authors stated in the introduction, that different taxonomic profilers (with different parameters settings) can produce very different taxonomic representations, to

support this statement it will be important to be able to show, at least one case, where TAMPA can suggest a different taxonomic interpretation of a microbial community that is also biologically relevant.

Response: We thank the reviewer for this insightful comment. We have included a new subsection in our Results section containing an example where results from TAMPA is able to provide additional biologically relevant insights compared to standard metrics of a microbial community that are biologically relevant. In particular, we applied the two best-performing taxonomic profilers MetaPhyler 2.2 and mOTUs2.5.1 to the marine dataset published in the "Critical Assessment of Metagenome Interpretation: the second round of challenges" (https://doi.org/10.1038/s41592-022-01431-4). On comparing the output of MetaPhlAn with the gold standard, we use TAMPA to demonstrate that while MetaPhlAn was one of the top-performing profilers, it was not able to detect several clades, including Planctomycetes (Figure 4a). Additionally, while MetaPhlAn and mOTUs were both the top-performing profilers in terms of single-valued metrics such as F1-score and L1 norm error, there were several critical differences between their outputs, highlighted by TAMPA at the phylum level (Figure 4 b). For example, mOTUs failed to detect the Tenericutes group, while MetaPhlAn failed to detect Planctomycetes. There was also a considerable difference in the percentage abundance of other groups, such as Firmicutes.Note that this data set is simulated, though the corresponding ground truth was not revealed to the taxonomic profilers. Using simulated data is required in this setting since we need to know for sure what the "correct" answer is. If we used real biological data, further analyses and followup studies would be required to validate the hypothesis generated by TAMPA, which is beyond the scope of this manuscript. We have added text to the discussion mentioning how TAMPA can be used for *hypothesis generation*, since confirming biological discoveries certainly requires more analyses than the visualizations returned by TAMPA.

Reviewer 2 Comment 3:
Figures in general appear to be of low-quality and stretched, please consider improving them as they are the main point of TAMPA.

Response: We thank the reviewer for this insight. We have replaced the figures in the manuscript with high-quality figures. We have also attached the high-quality figures separately in our submission for your reference.
