# Supplementary material for: TAMPA: interpretable analysis and visualization of metagenomics-based taxon abundance profiles
Source: Gigascience. 2023 Feb 28;12:giad008. doi: 10.1093/gigascience/giad008 (PMC9972184; doi:10.1093/gigascience/giad008)
Supplement: giad008_Supplemental_File [file giad008_supplemental_file.docx]

# **Supplementary Materials**

# **Supplementary Figures**


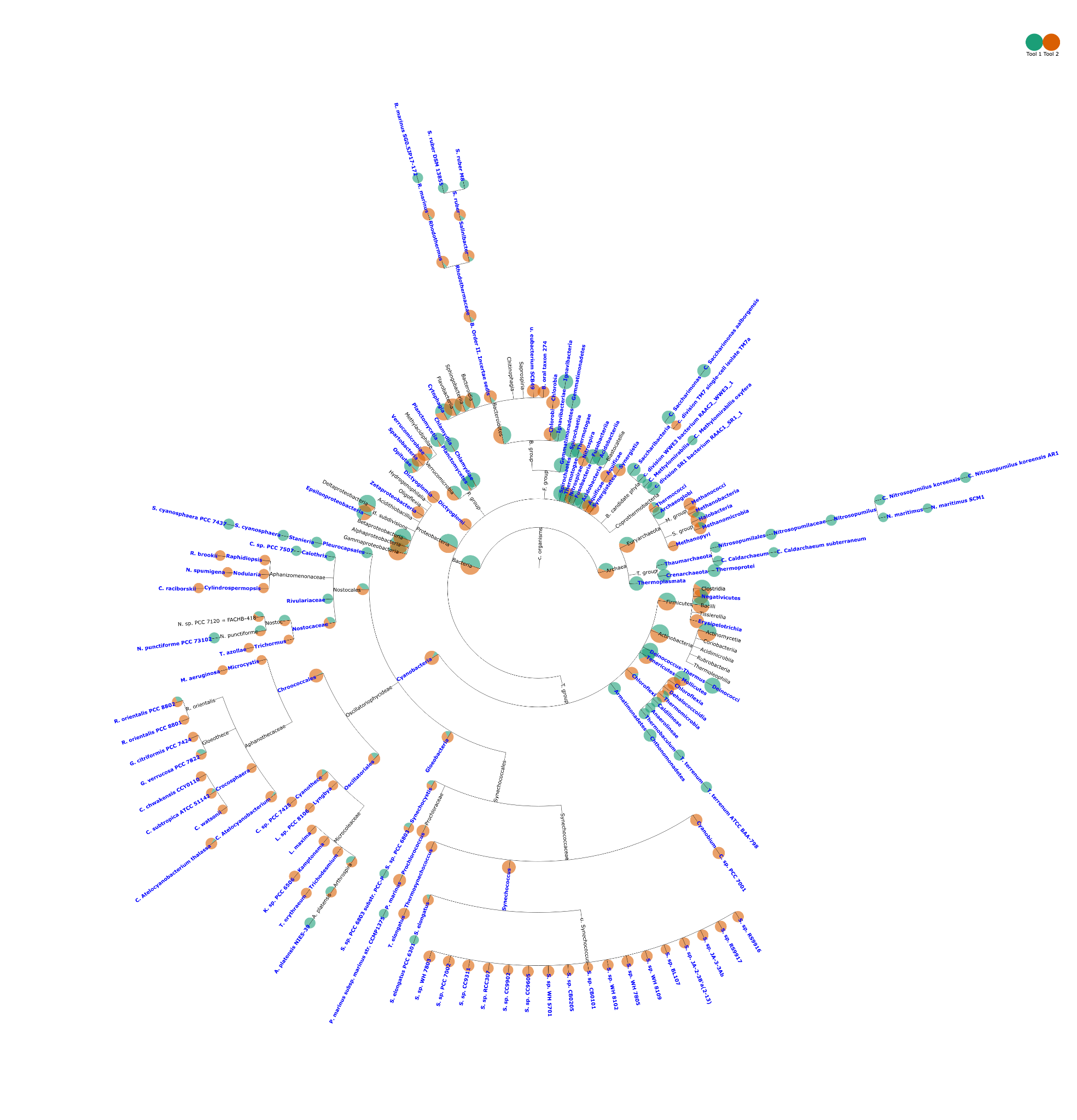


Figure S1: Visualization of the taxonomic profiles of tools with identical UniFrac scores of 4, Taxy_pro vs Metaphyler using TAMPA on the CAMI dataset at the class rank. Note the differences in taxa predictions even though the tools have identical UniFrac scores.


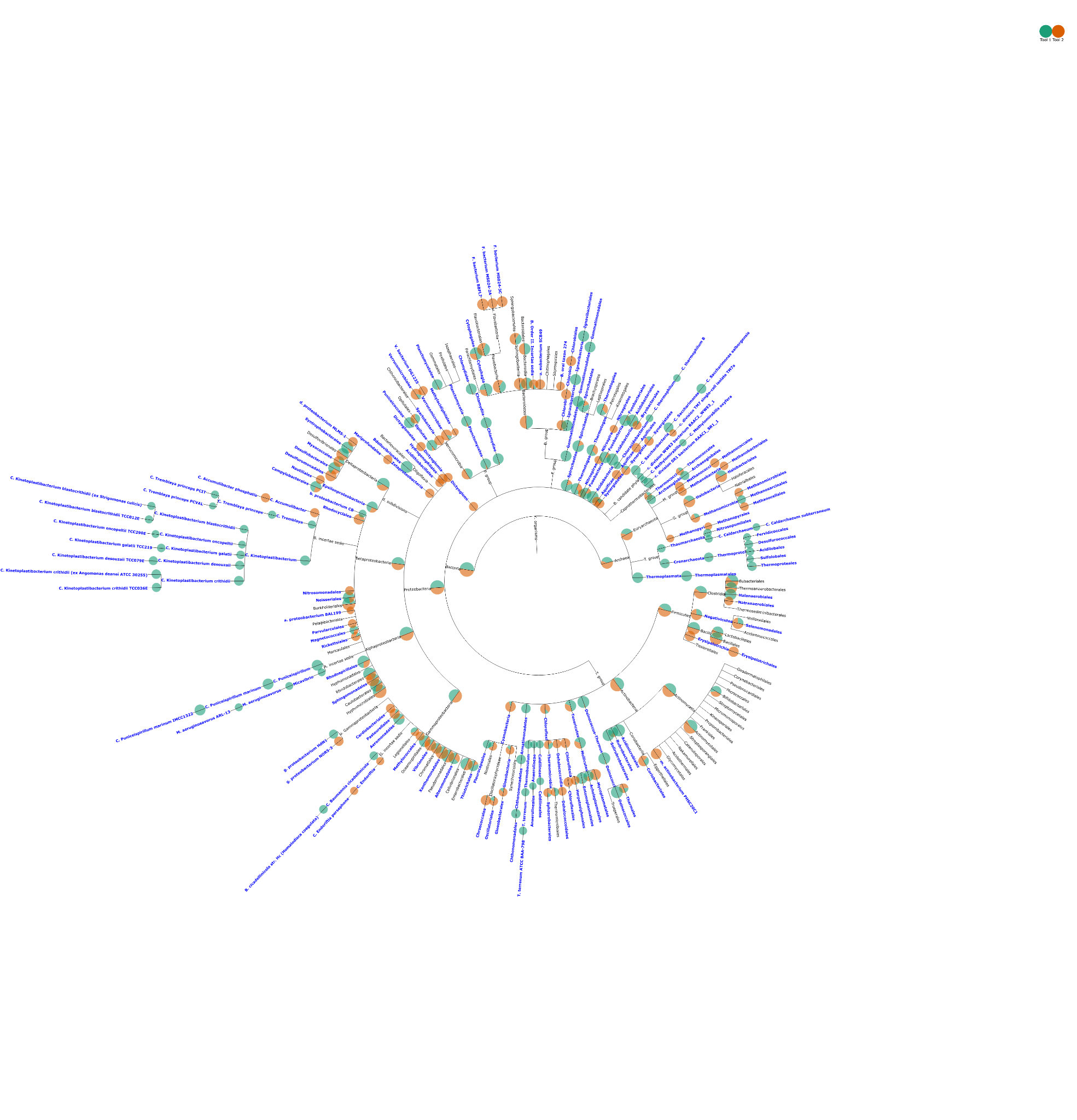


Figure S2: Visualization of the taxonomic profiles of tools with identical UniFrac scores of 4, Taxy_pro vs Metaphyler using TAMPA on the CAMI dataset at the order rank.


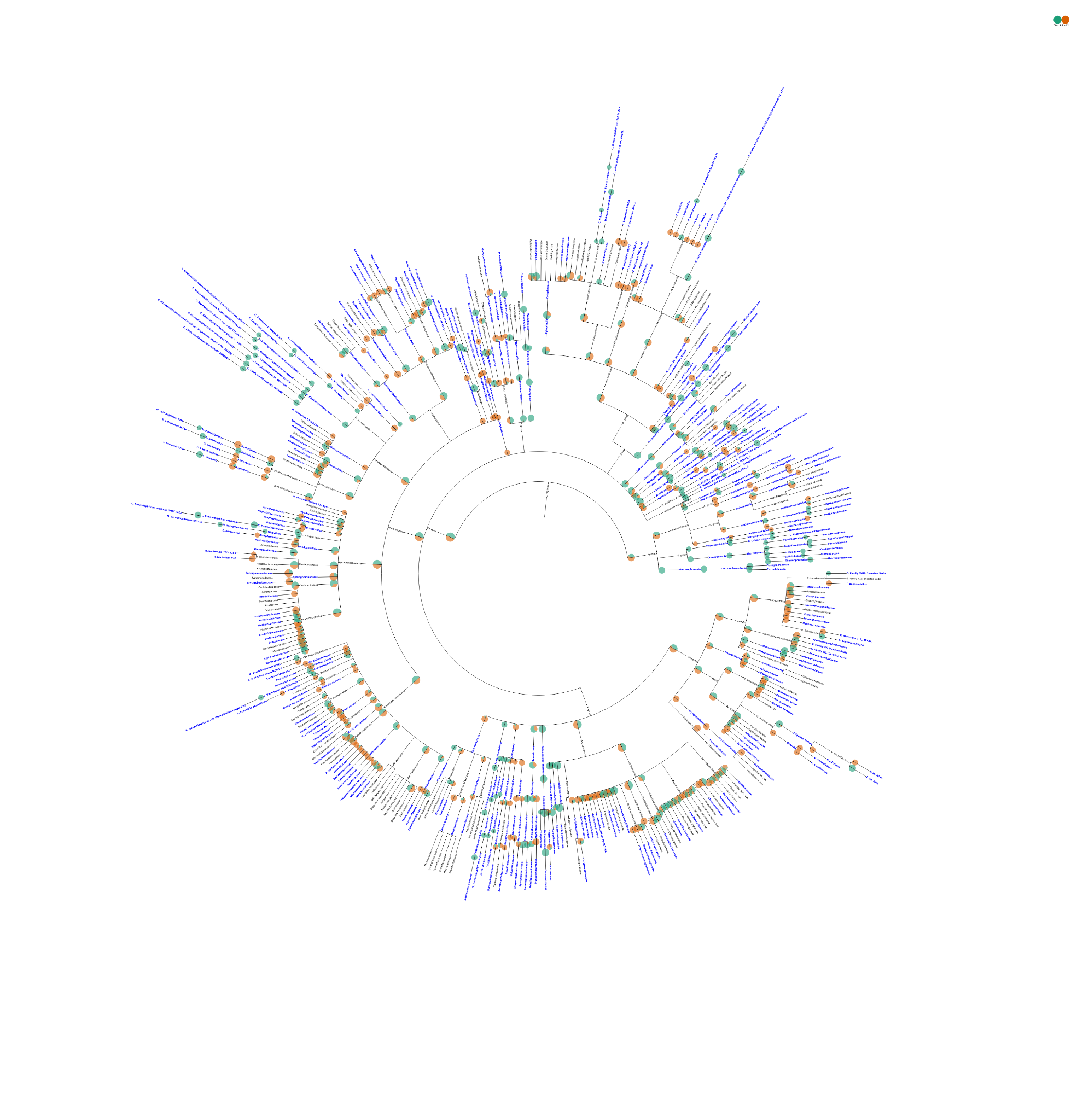


Figure S3: Visualization of the taxonomic profiles of tools with identical UniFrac scores of 4, Taxy_pro vs Metaphyler using TAMPA on the CAMI dataset at the family rank


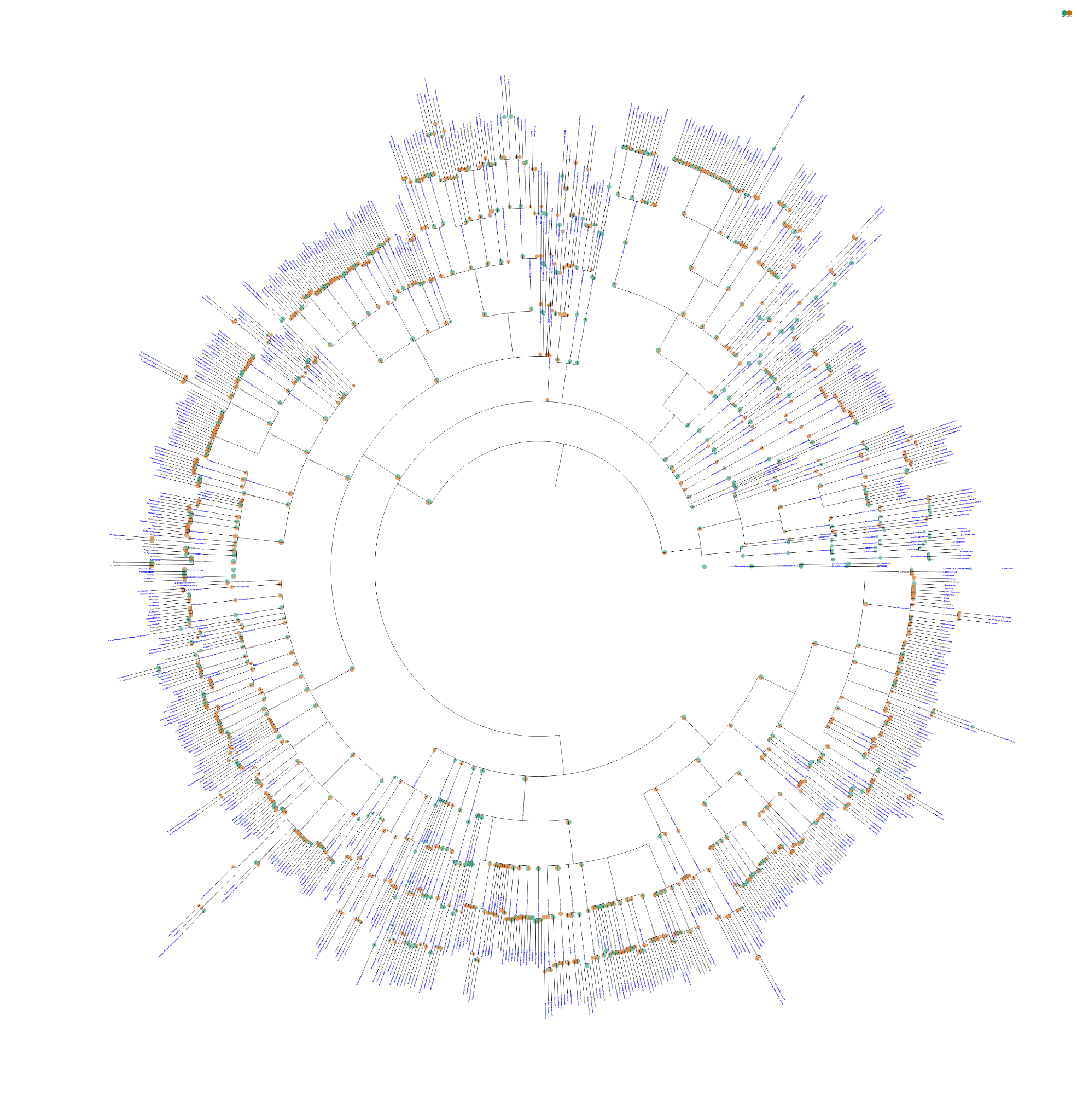


Figure S4: Visualization of the taxonomic profiles of tools with identical UniFrac scores of 4, Taxy_pro vs Metaphyler using TAMPA on the CAMI dataset at the genus rank


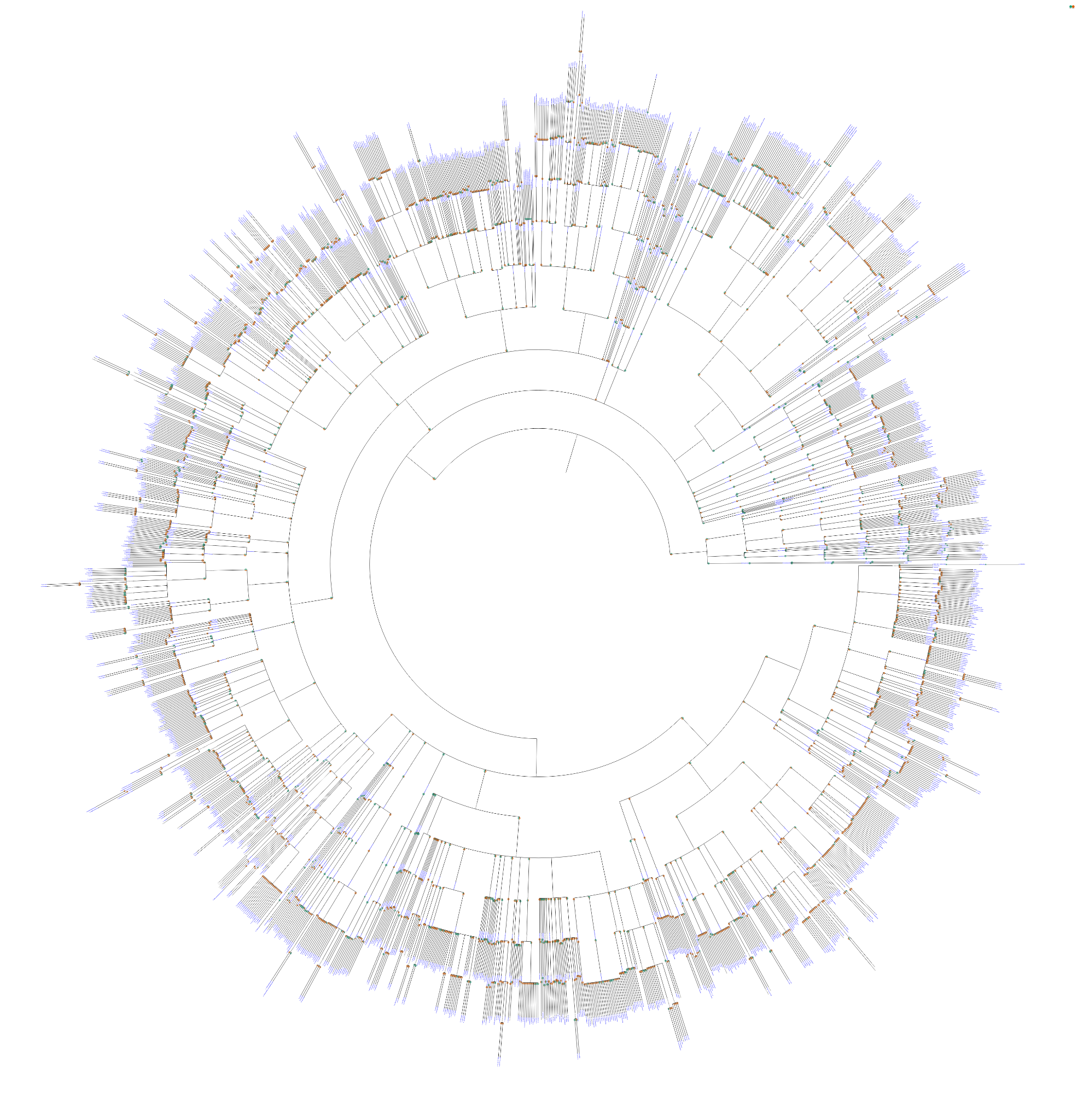


Figure S5: Visualization of the taxonomic profiles of tools with identical UniFrac scores of 4, Taxy_pro vs Metaphyler using TAMPA on the CAMI dataset at the species rank


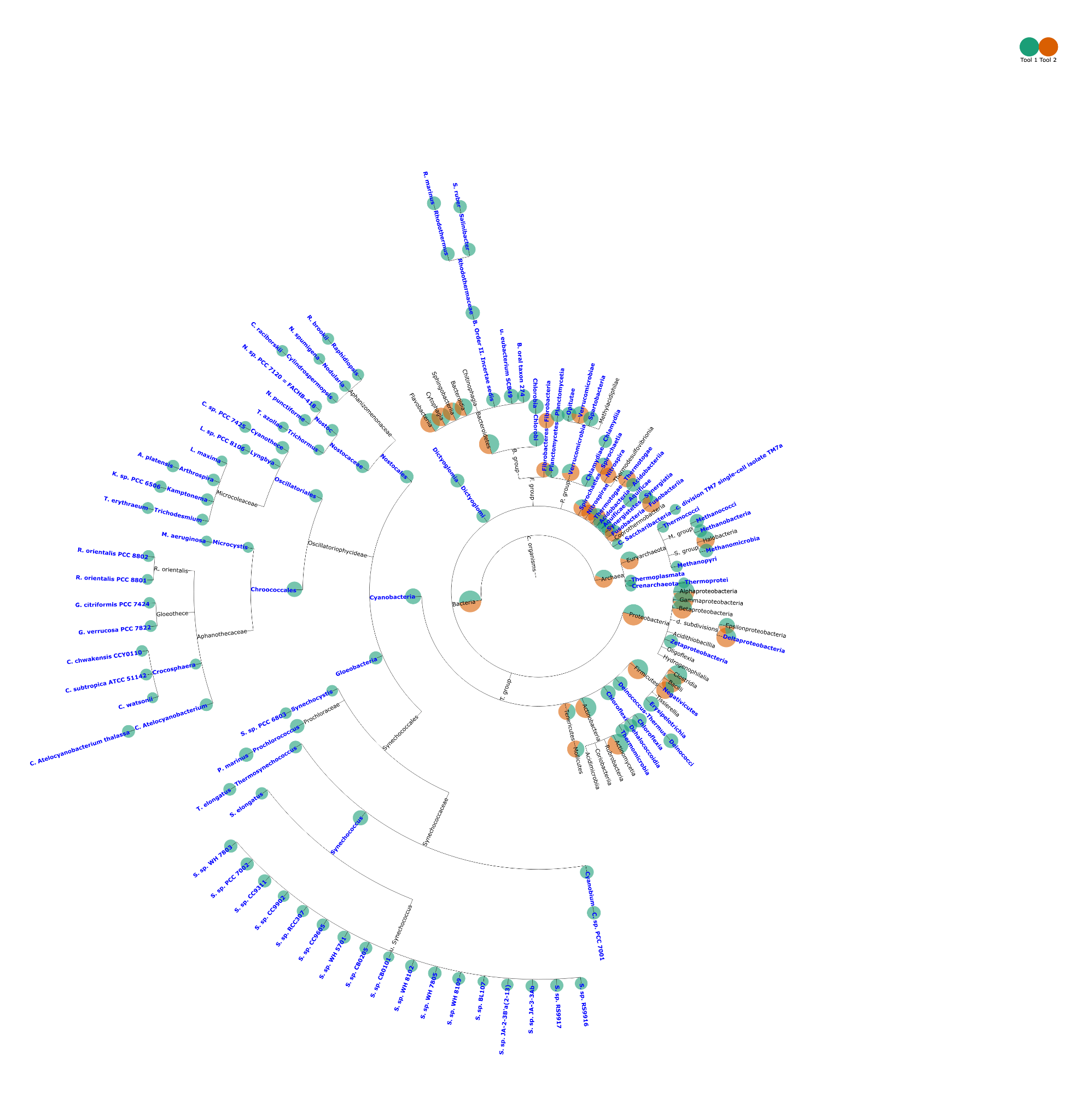


Figure S6: Visualization of the taxonomic profiles of a top performing CAMI tool, Metaphyler vs the ground truth using TAMPA on the CAMI dataset at the class level.


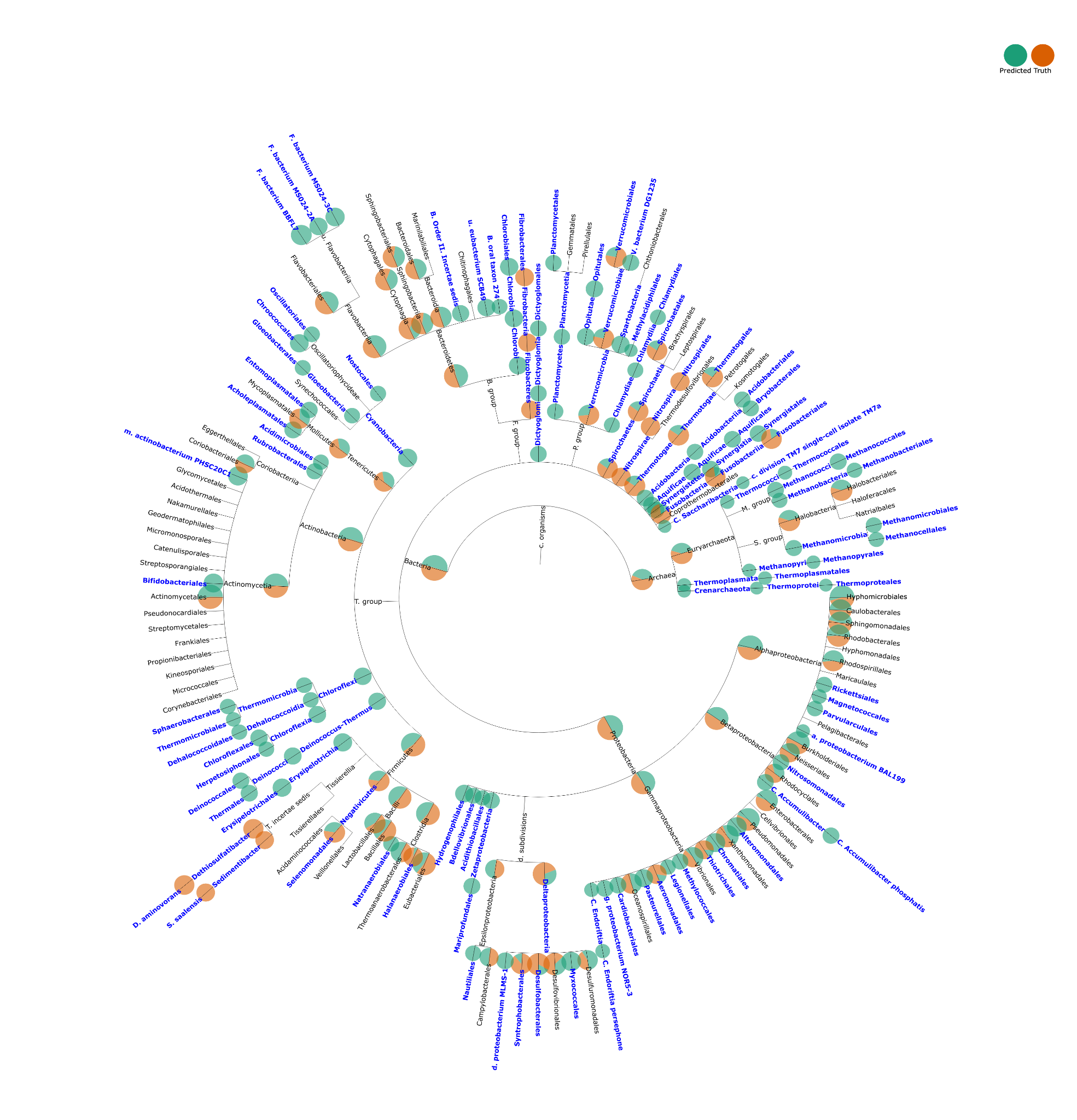


Figure S7: Visualization of the taxonomic profiles of a top performing CAMI tool, Metaphyler vs the ground truth using TAMPA on the CAMI dataset at the order level.

a)


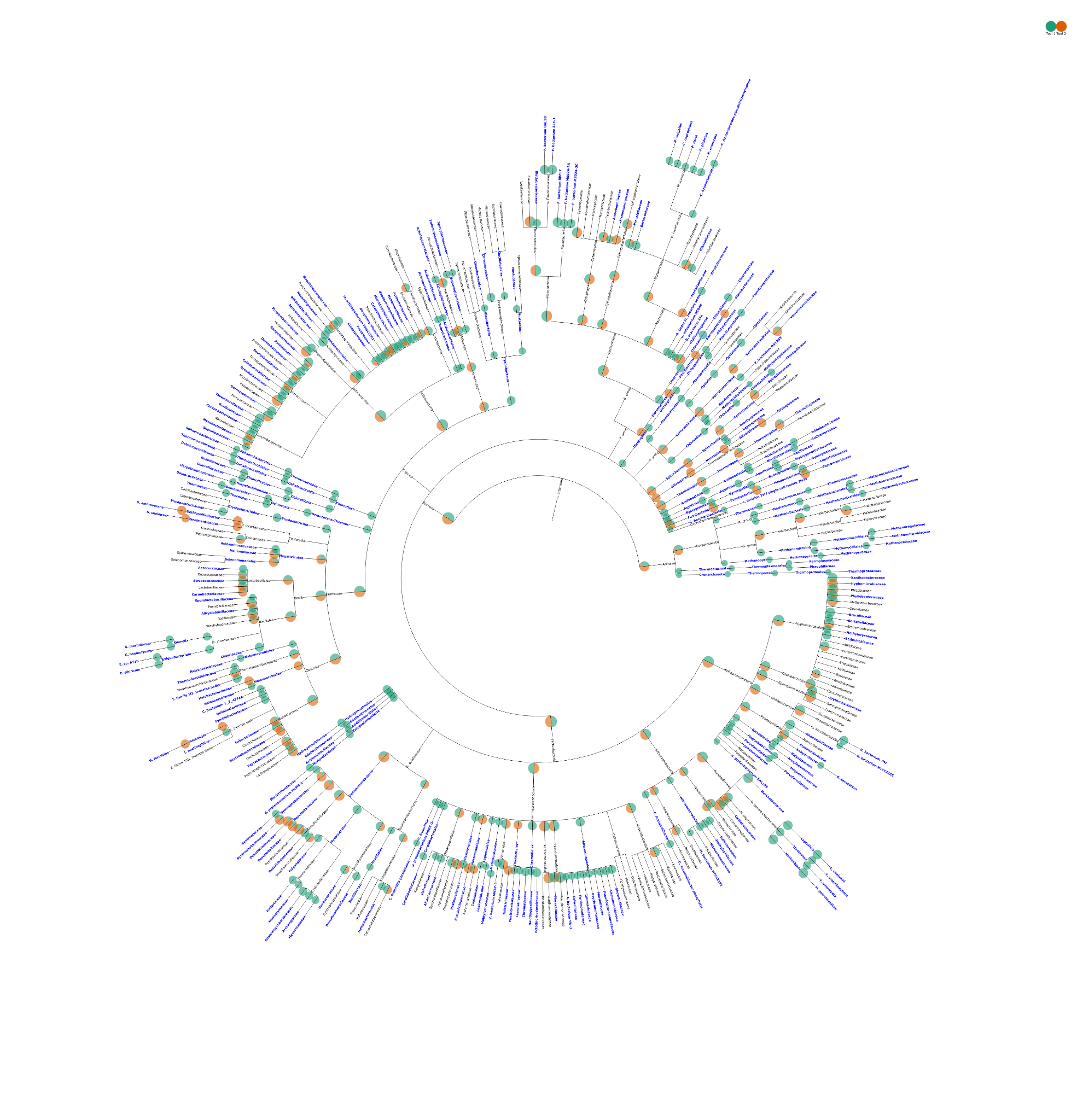


b)


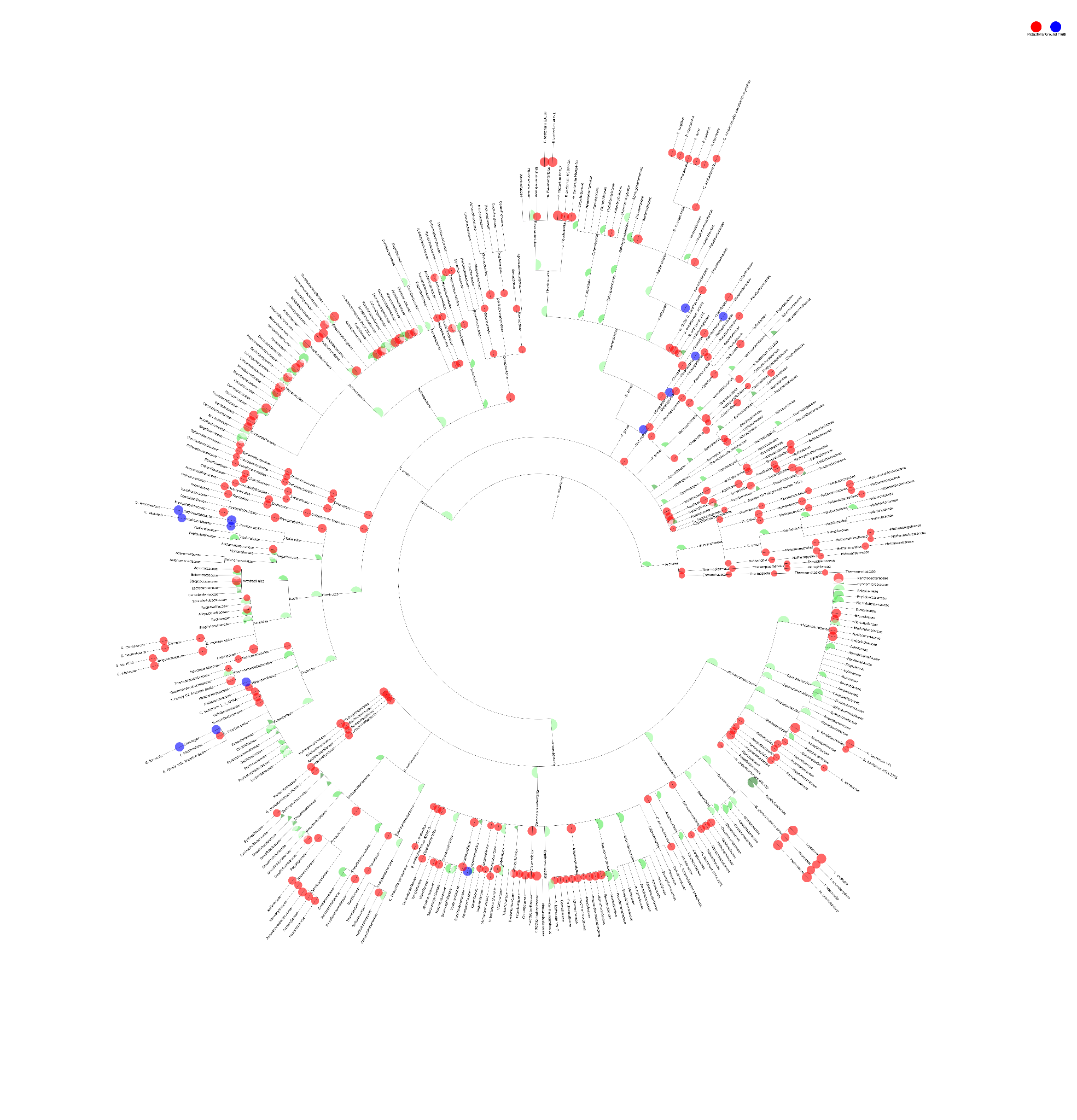


Figure S8: a) Visualization of the taxonomic profiles of a top performing CAMI tool, Metaphyler vs the ground truth using TAMPA on the CAMI dataset at the family level b) With contrast mode

a)


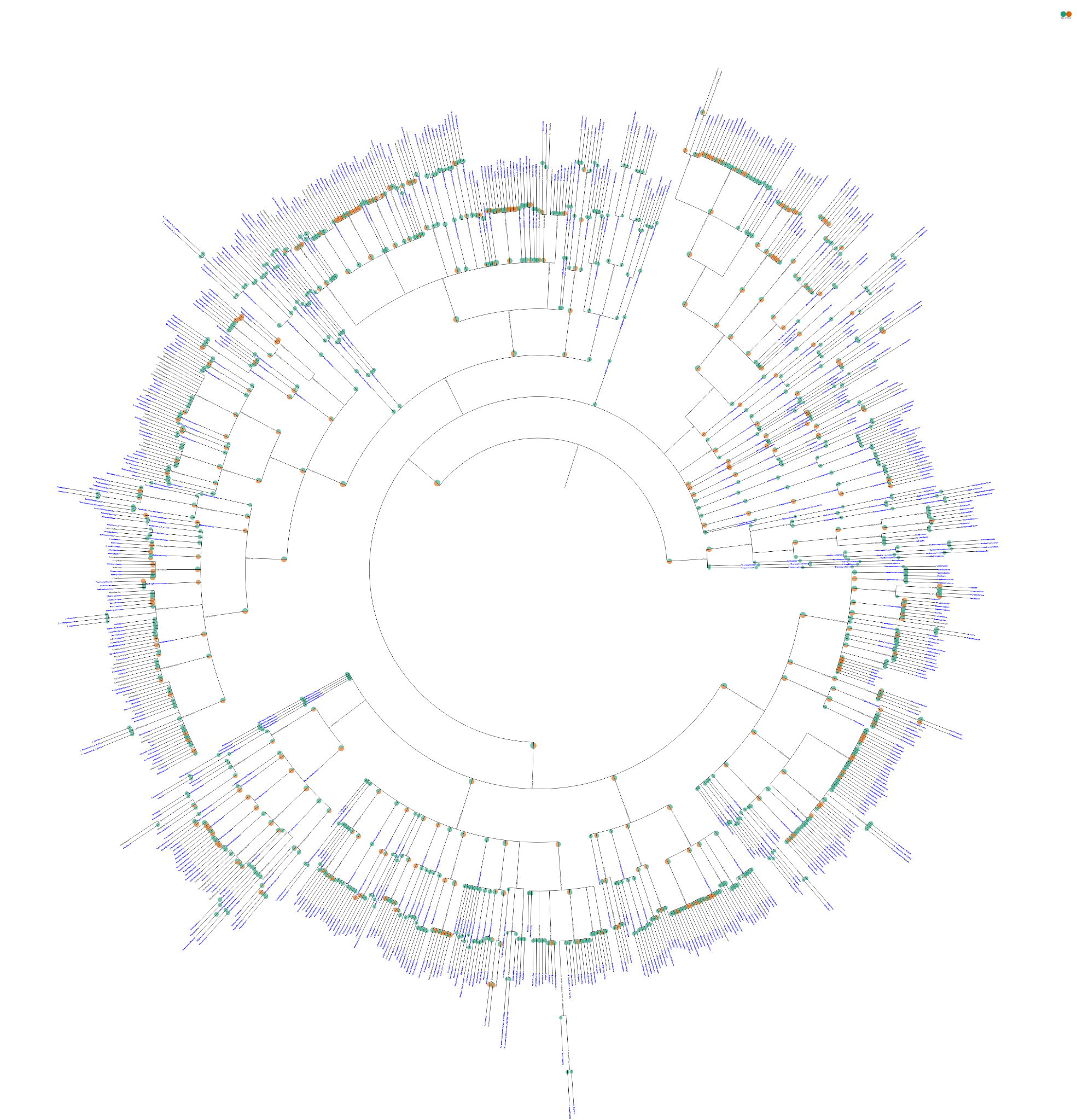


b)


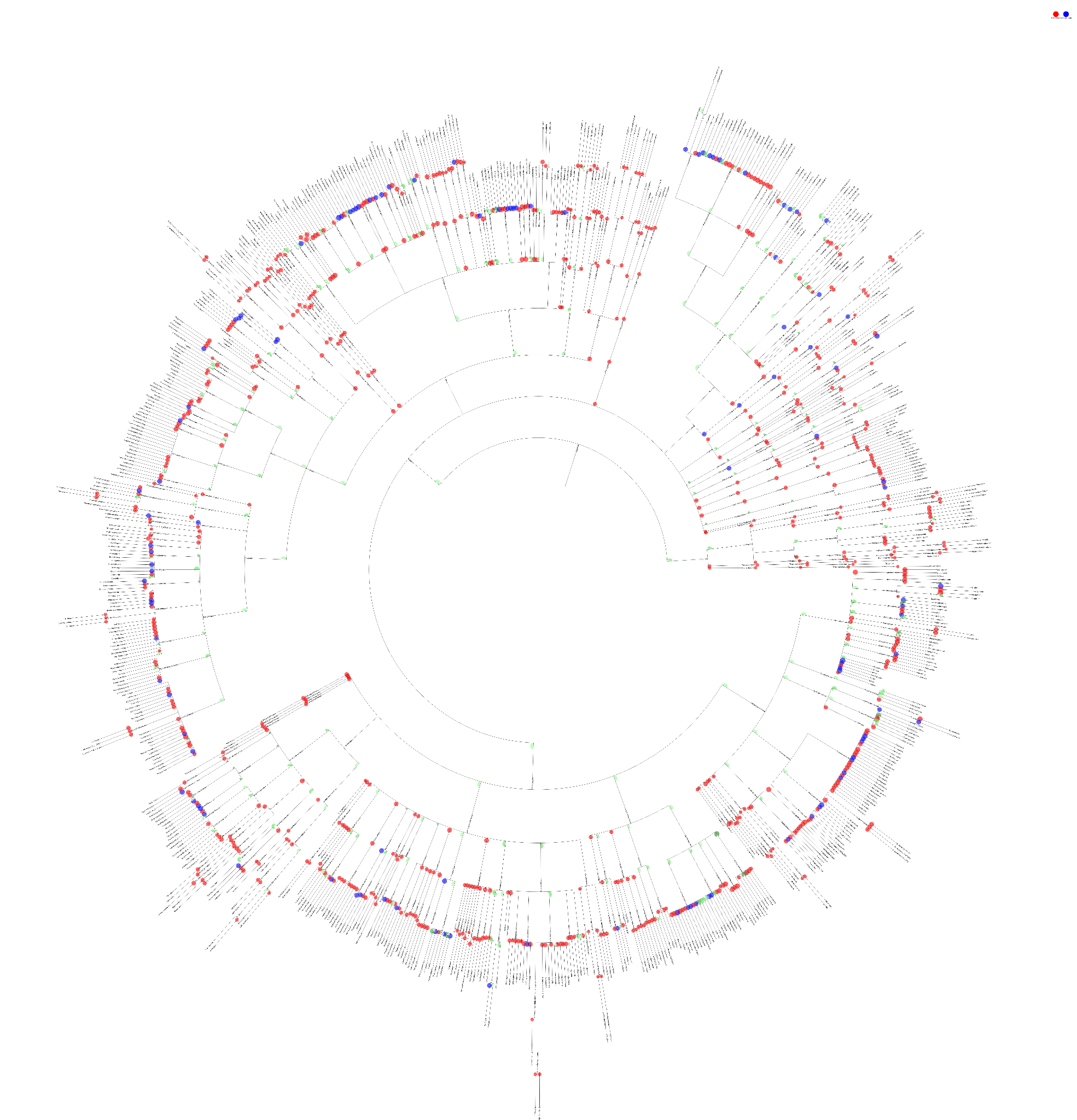


Figure S9: a) Visualization of the taxonomic profiles of a top performing CAMI tool, Metaphyler vs the ground truth using TAMPA on the CAMI dataset at the genus level b) With contrast mode.

a)


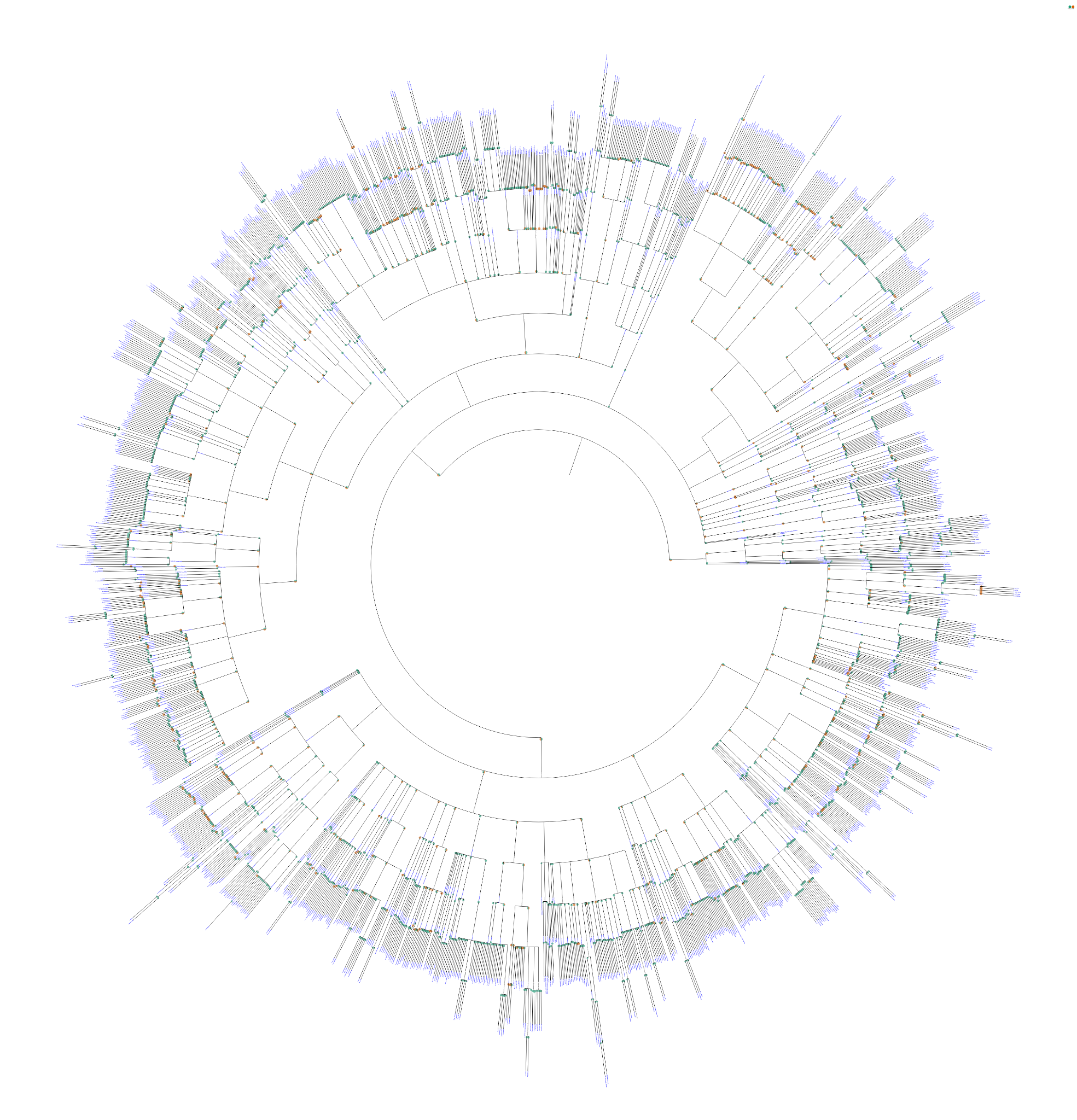


b)
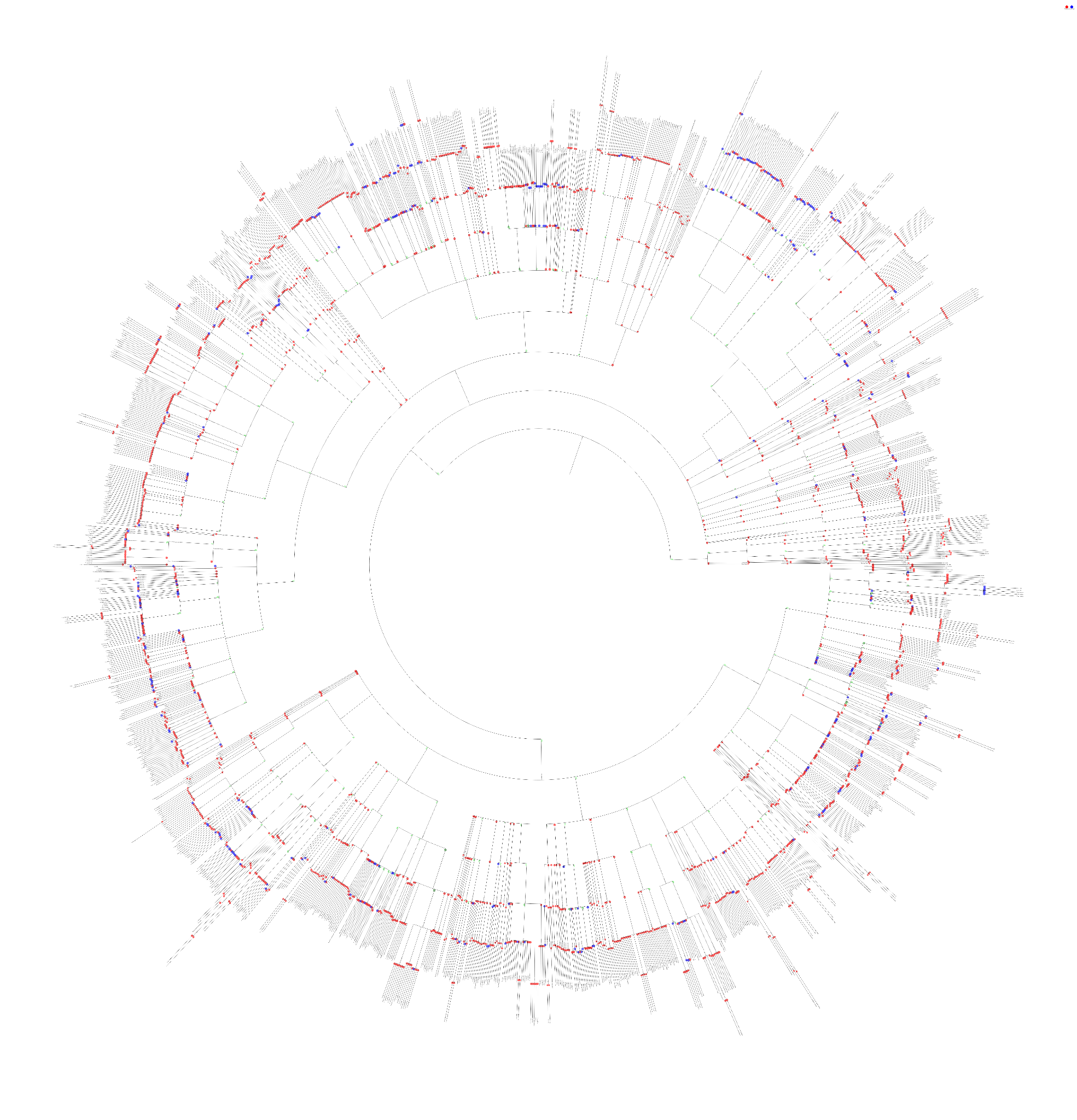


Figure S10: Visualization of the taxonomic profiles of a top performing CAMI tool, Metaphyler vs the ground truth using TAMPA on the CAMI dataset at the species level b) With contrast mode.


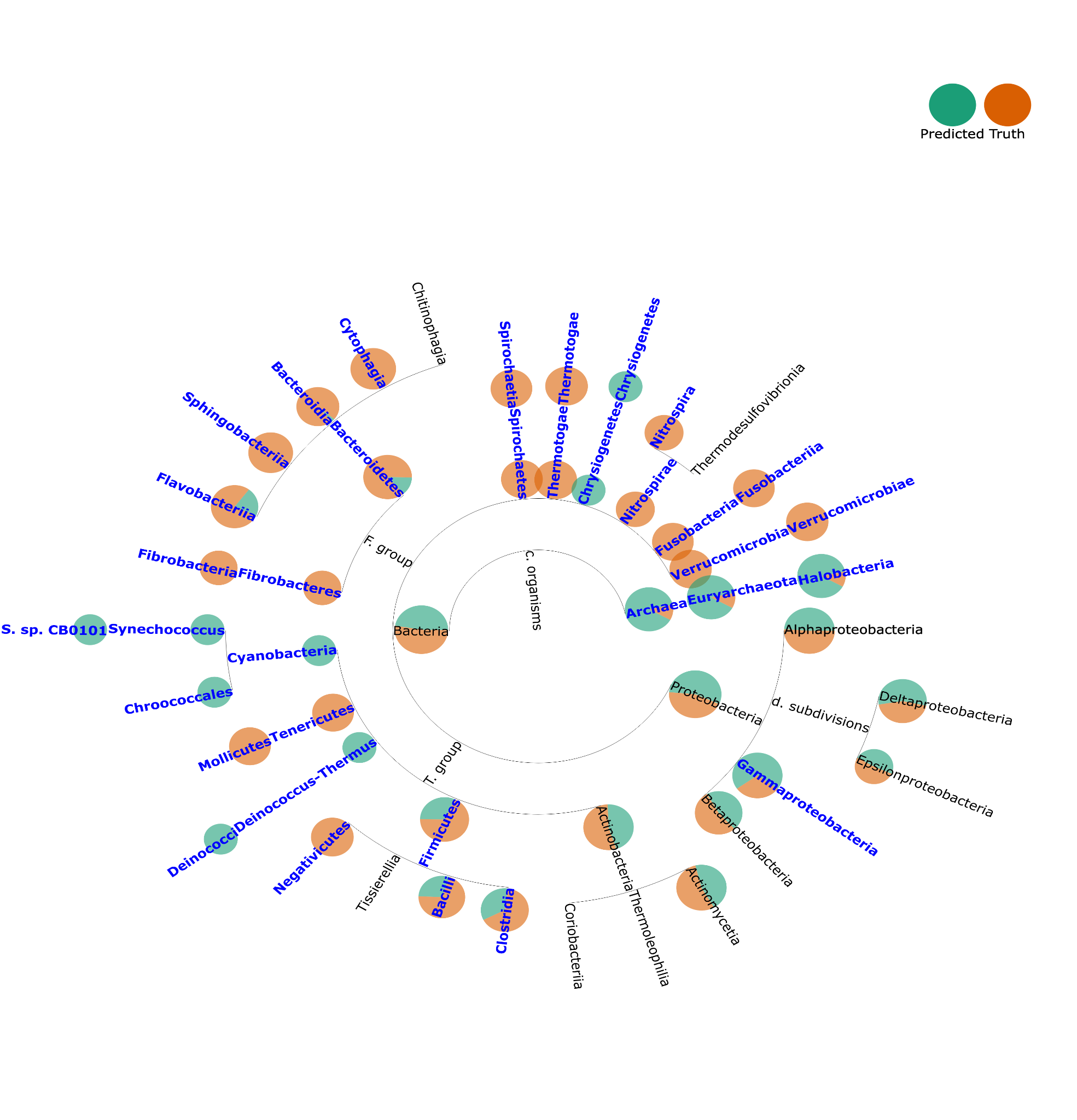


Figure S11: Visualization of the taxonomic profiles of the lowest performing tool, mOTU vs the ground truth using TAMPA on the CAMI dataset at the class level.


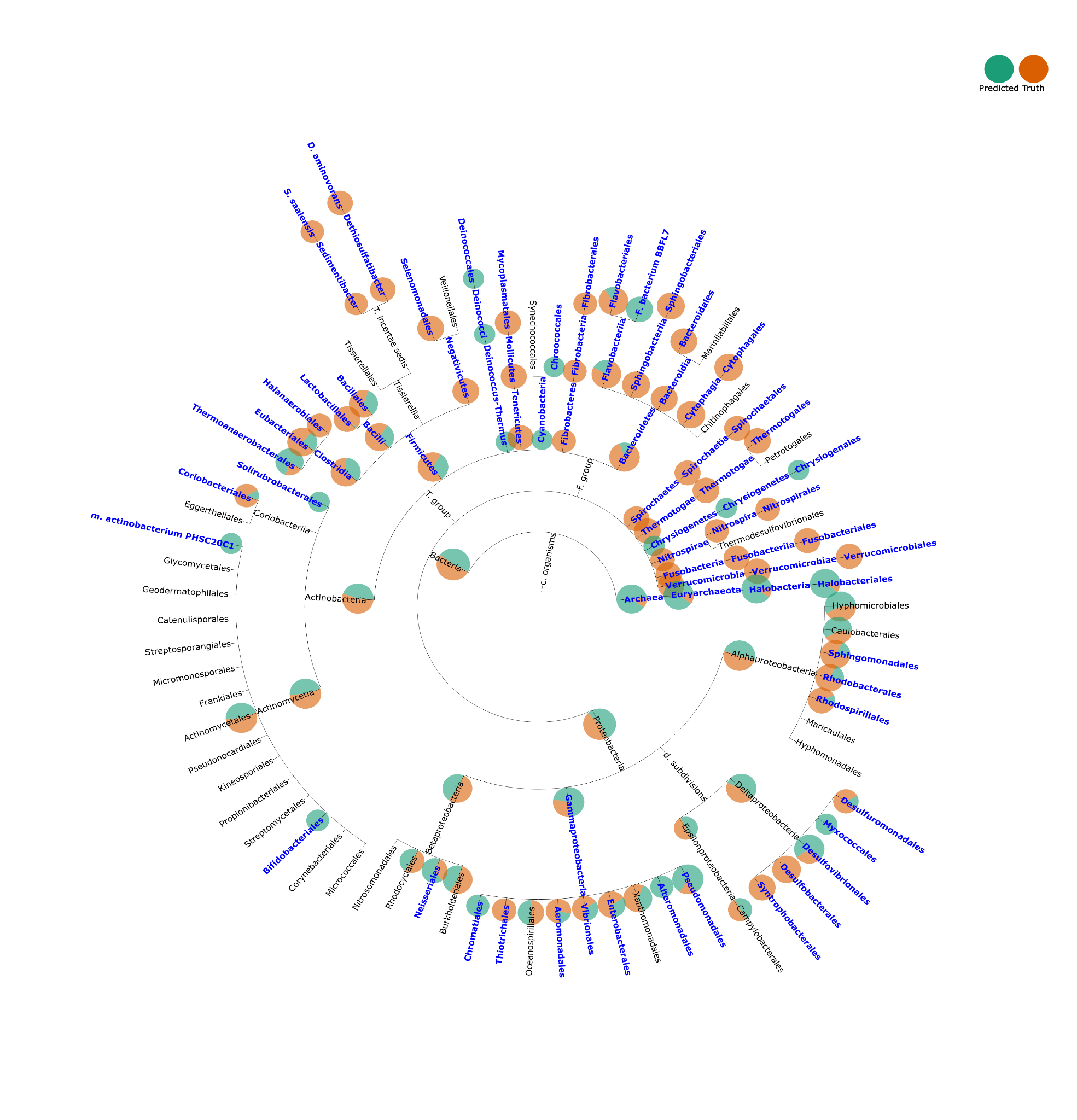


Figure S12: Visualization of the taxonomic profiles of the lowest performing tool, mOTU vs the ground truth using TAMPA on the CAMI dataset at the order level.


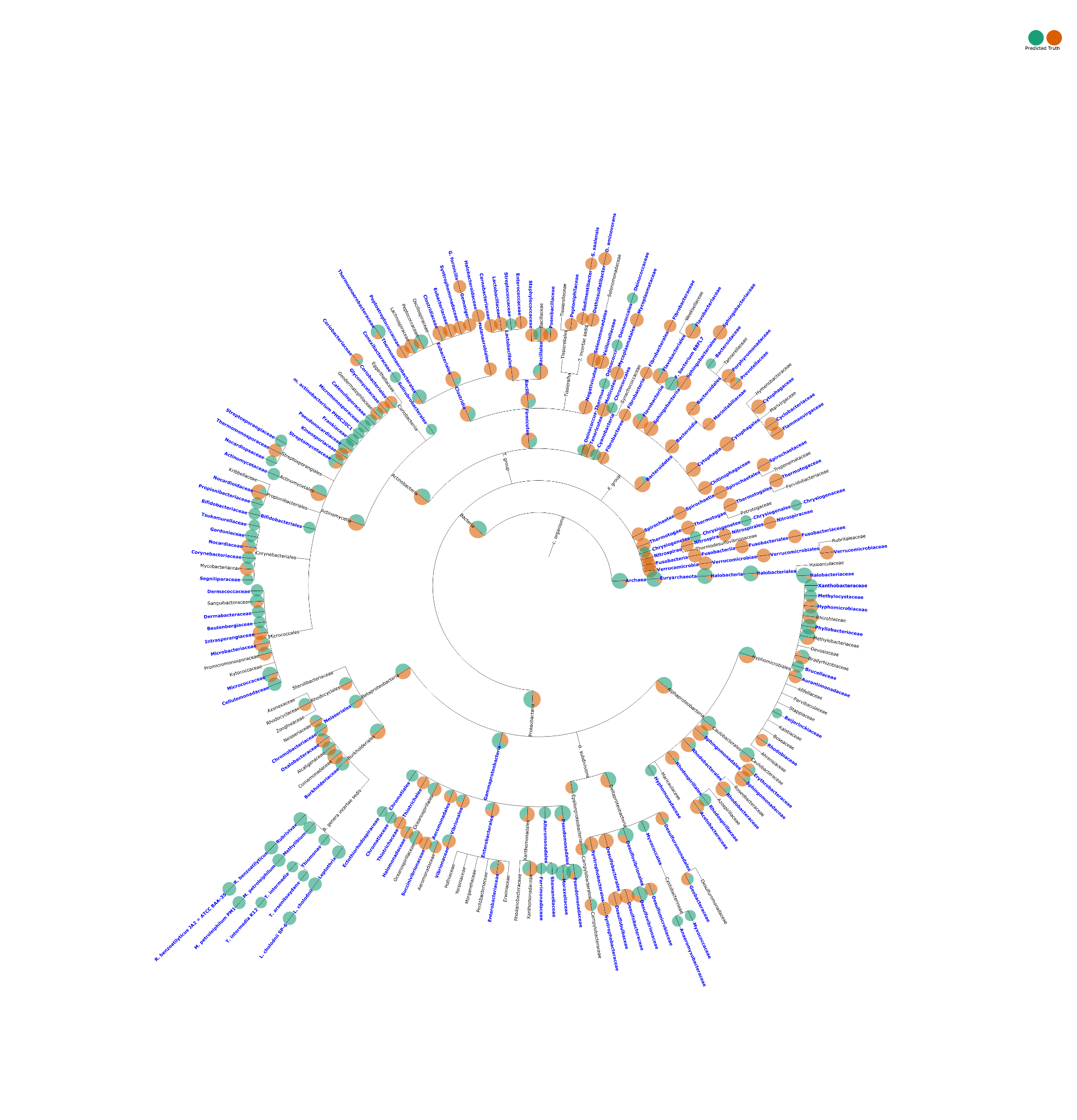


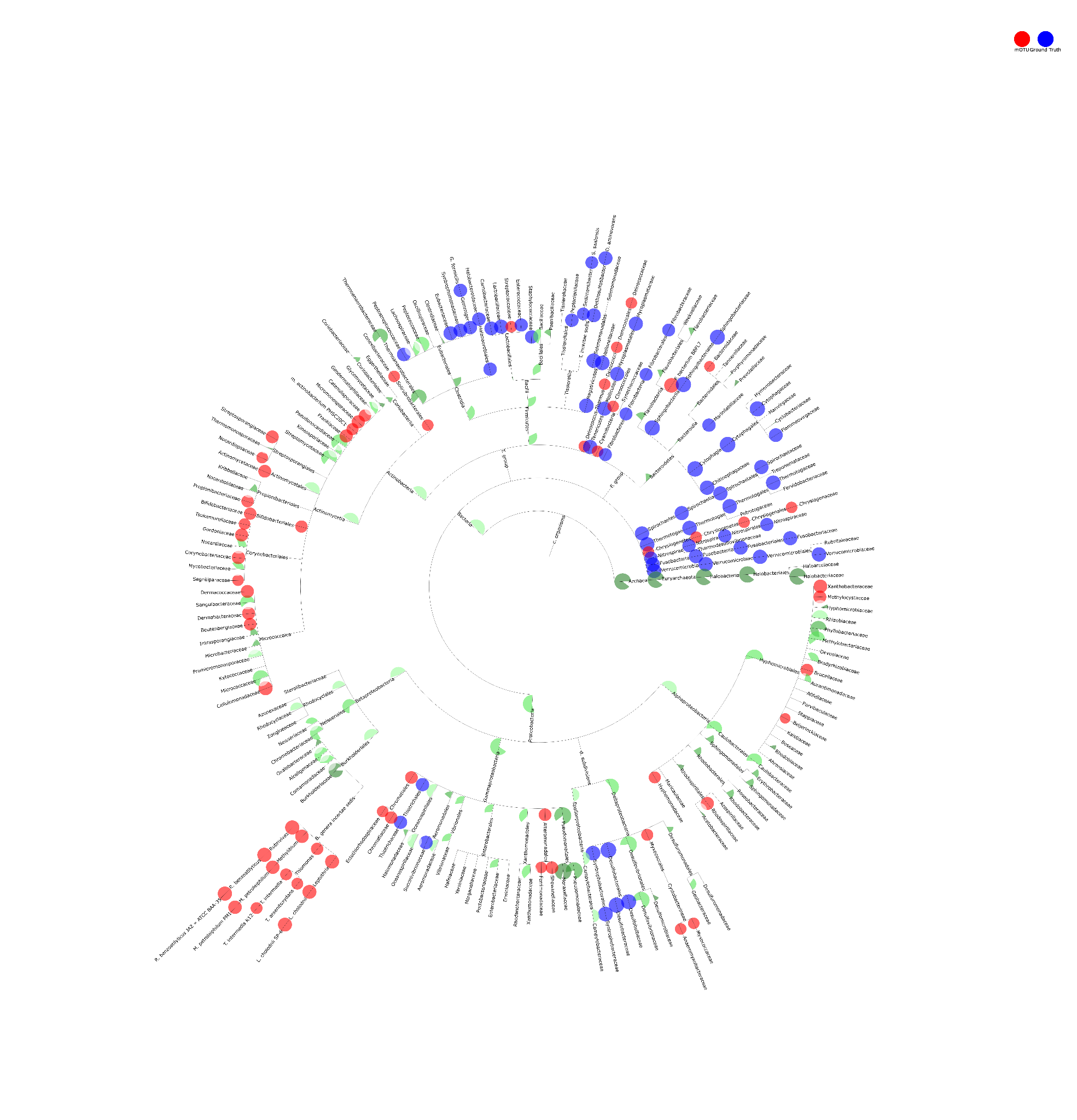


Figure S13: a) Visualization of the taxonomic profiles of the lowest performing tool, mOTU vs the ground truth using TAMPA on the CAMI dataset at the family level b) With contrast mode.

a)


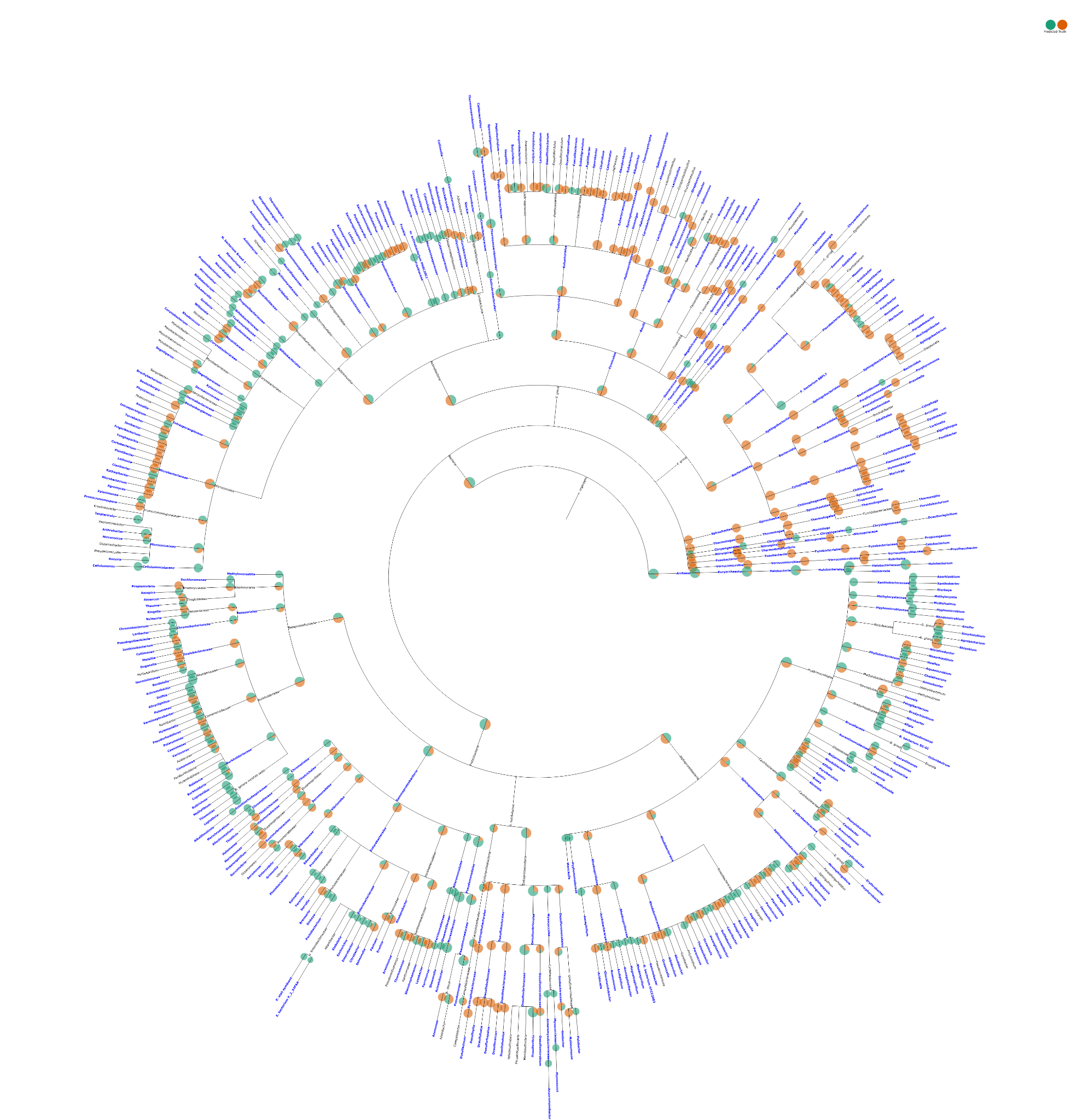


b)


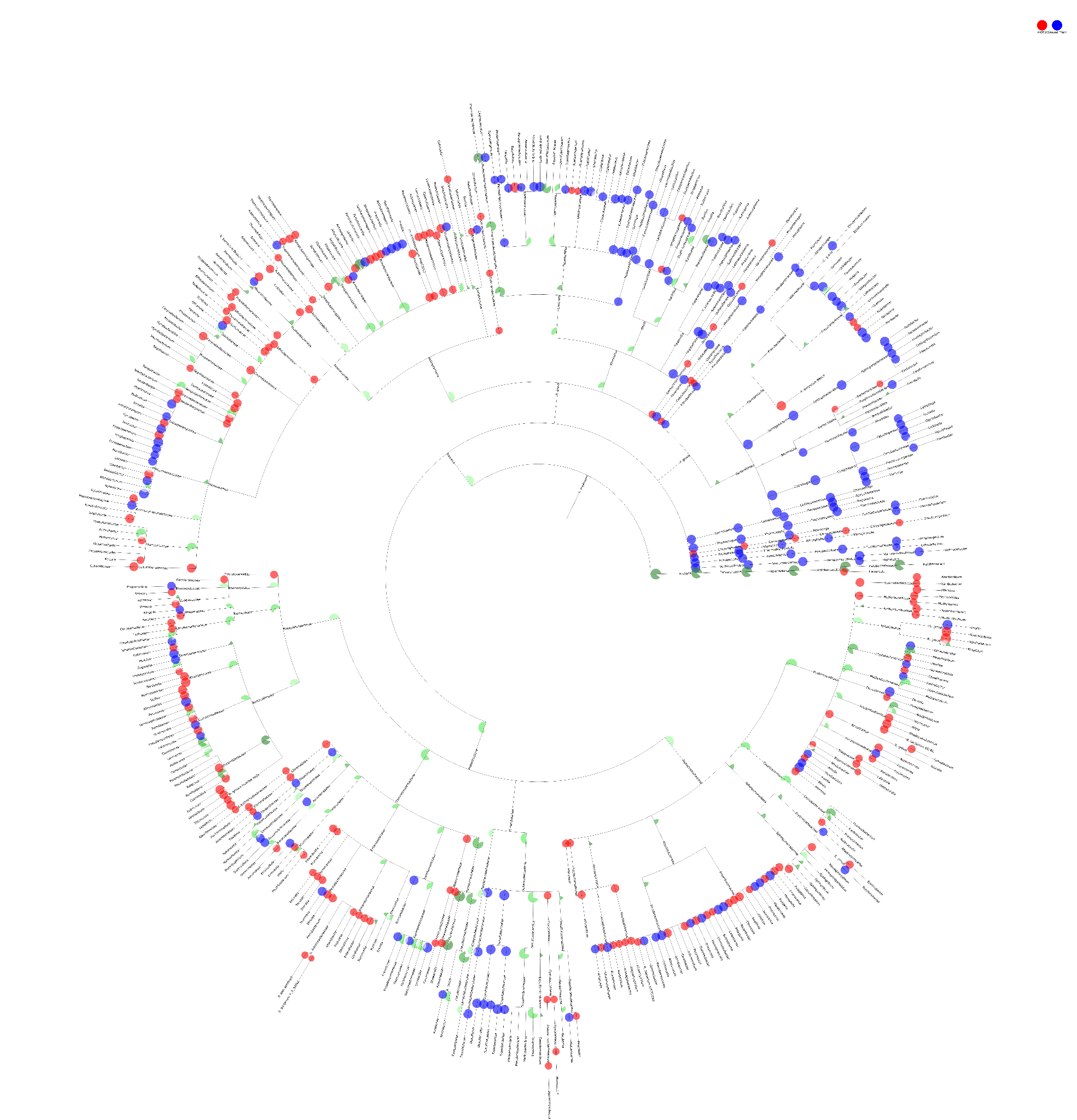


Figure S14: a) Visualization of the taxonomic profiles of the lowest performing tool, mOTU vs the ground truth using TAMPA on the CAMI dataset at the genus level b) With contrast mode.

a)


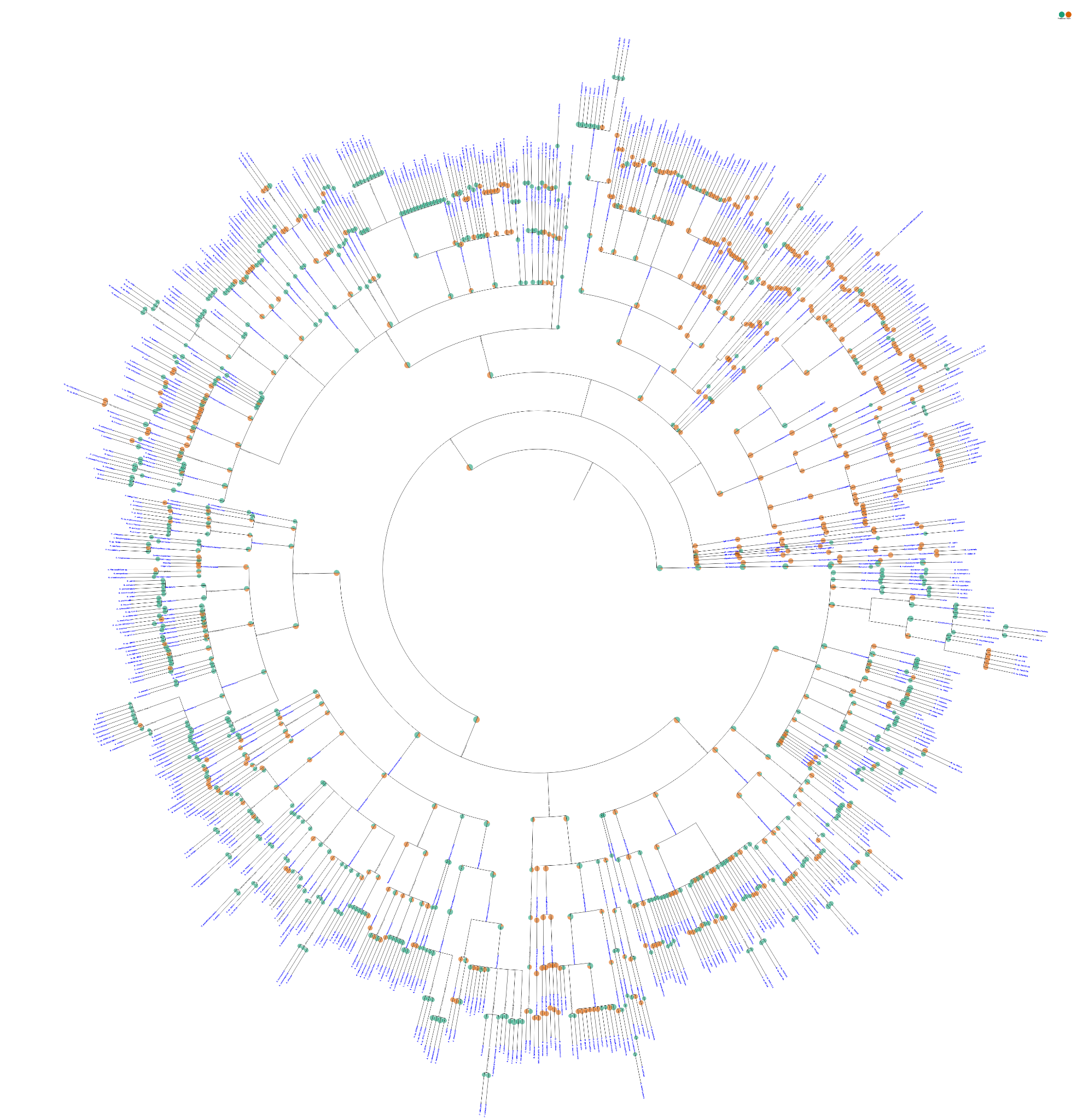


b)


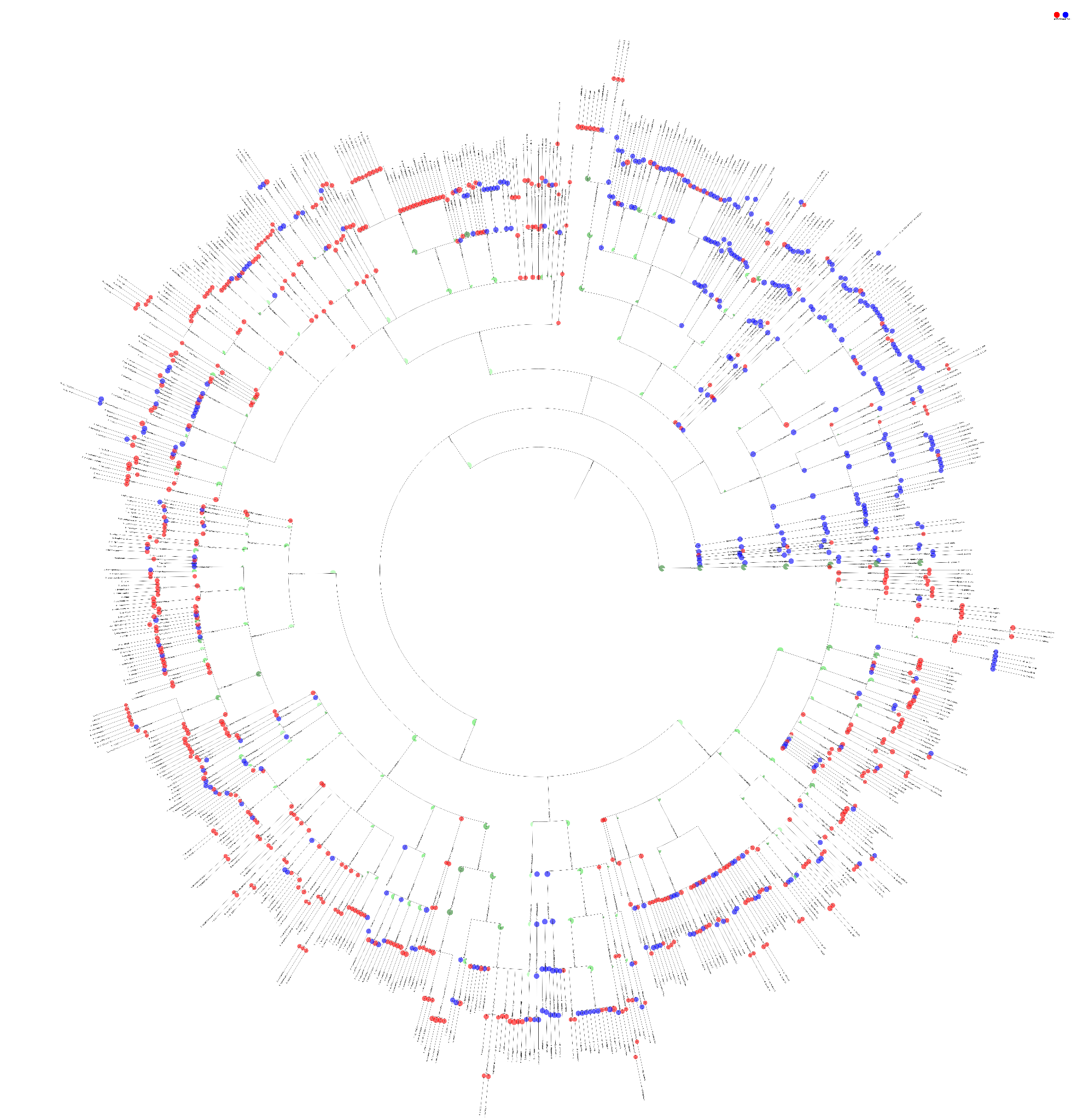


Figure S15: Visualization of the taxonomic profiles of the lowest performing tool, mOTU vs the ground truth using TAMPA on the CAMI dataset at the species level b) With contrast mode.


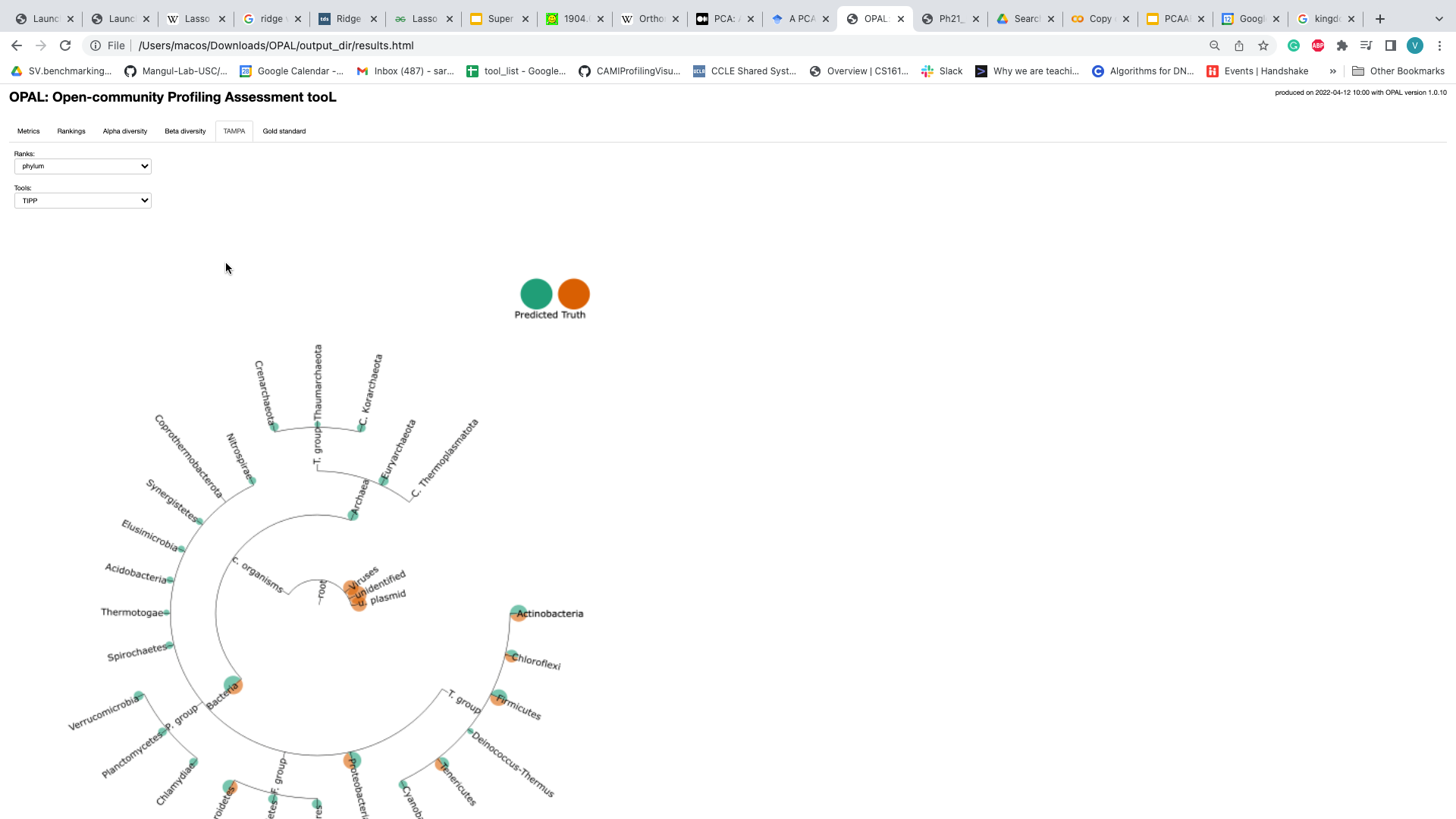


Figure S16: Incorporation of TAMPA into OPAL


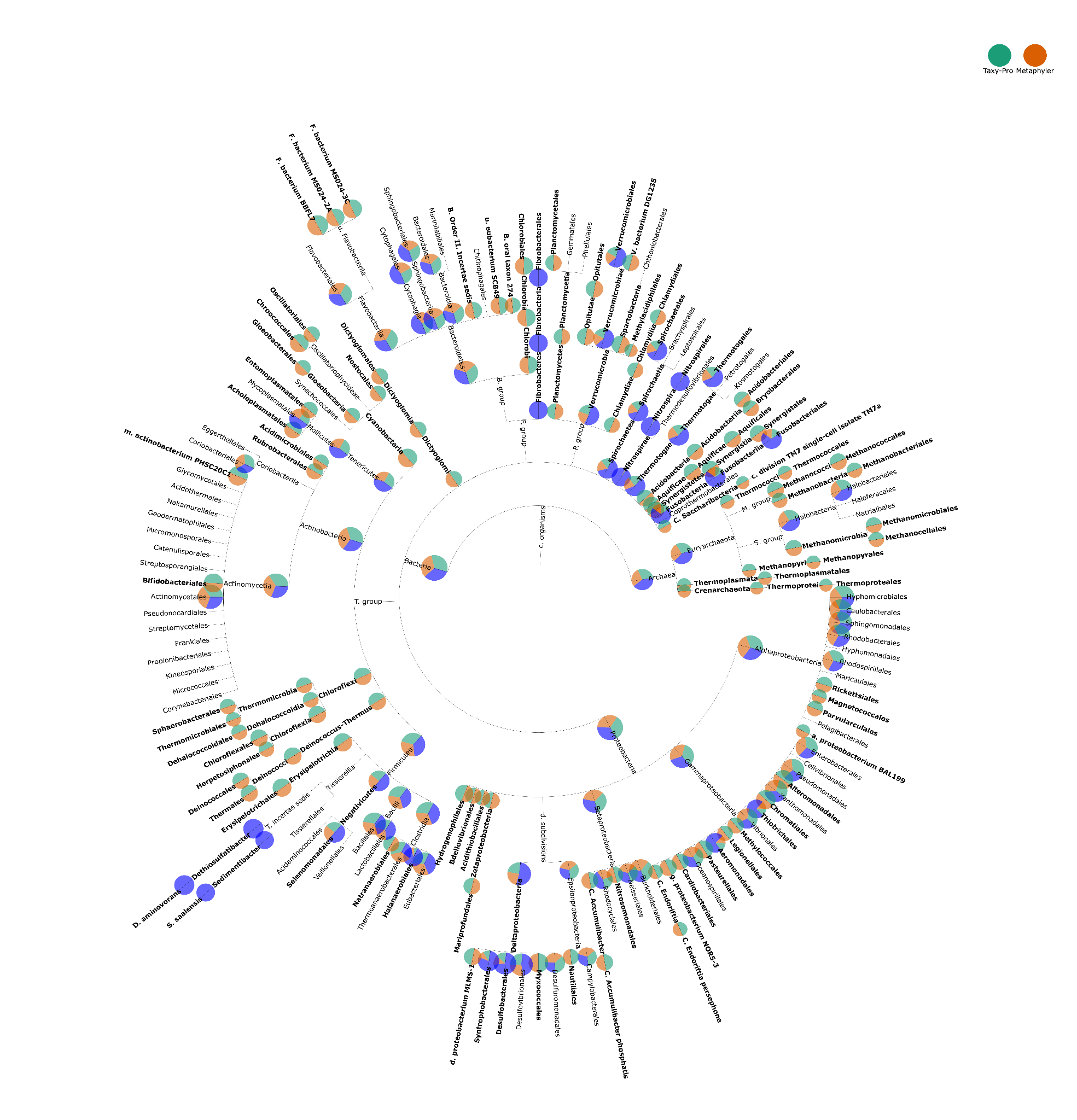


Figure S17: Visualization of the taxonomic profiles of three tools: Taxy_pro (green) vs Metaphyler (orange) vs mOUT (blue). using TAMPA on the CAMI dataset at the class rank.
