# Supplementary material for: TAMPA: interpretable analysis and visualization of metagenomics-based taxon abundance profiles
Source: Gigascience. 2023 Feb 28;12:giad008. doi: 10.1093/gigascience/giad008 (PMC9972184; doi:10.1093/gigascience/giad008)
Supplement: giad008_Response_to_Reviewer_Comments_Original_Submission [file giad008_response_to_reviewer_comments_original_submission.pdf]

Author's Response To Reviewer Comments

Close

Reviewer 1 Comment 1:
The tool should be easy to install, I would suggest to add it to bioconda, which allow the installation of all dependencies (see CODE EVALUATION below).
CODE EVALUATION --

I downloaded with conda (`conda install -c vsarwal tampa`) and have the following error:

```
$ profile_to_plot
Traceback (most recent call last):
File "/miniconda3/bin/profile_to_plot", line 33, in
sys.exit(load_entry_point('CAMIViz==0.1.0', 'console_scripts', 'profile_to_plot')())
File "/miniconda3/bin/profile_to_plot", line 25, in importlib_load_entry_point
return next(matches).load()
File "/miniconda3/lib/python3.7/site-packages/importlib_metadata/__init__.py", line 167, in load
module = import_module(match.group('module'))
File "/miniconda3/lib/python3.7/importlib/__init__.py", line 127, in import_module
return _bootstrap._gcd_import(name[level:], package, level)
File "", line 1006, in _gcd_import
File "", line 983, in _find_and_load
File "", line 967, in _find_and_load_unlocked
File "", line 677, in _load_unlocked
File "", line 728, in exec_module
File "", line 219, in _call_with_frames_removed
File "/miniconda3/lib/python3.7/site-packages/src/profile_to_plot.py", line 2, in
from ProfilesLayout import ProfilesLayout
ModuleNotFoundError: No module named 'ProfilesLayout'
```

I have the same error if I follow the installation in https://github.com/dkoslicki/TAMPA.

In order to make the tool easier to use I would suggest to:
- change the name of the executable from `profile_to_plot` to `tampa`
- you could add the package to bioconda to have all the dependencies installed
- change the version to 1.0.0 for the release

Response: We thank the reviewer for pointing out these errors. We have added the package on bioconda with dependencies installed, changed the name of the executable from profile_to_plot to tampa, and changed the version to be 1.0.0 for the release.

Reviewer 1 Comment 2:
TAMPA has limited applicability when comparing to Ground Truth. The tool allow to compare two different profiles, but there are conceptually two different evaluations: 1) compare two different profiles, and 2) compare a profile to the Ground Truth. The current implementation works good for the first evaluation, but I can see it can be improved for the second. For example, it would be interesting to visualise as a red circle false positive taxa (for example in Figure 2 Cyanobacteria), in blue false negatives (Nitrospirae in Figure 2) and in a gradient of white to green when the taxa expected from the Ground truth is measured: in white if the relative abundance is the same and in green if the relative abundance is different between expected and measured (with the colour intensity proportional to the error). In this way all correct taxa will be white, and looking at the tree colors we could understand the issue: red (false positive), blue (false negative), green (measure the correct taxa, but with wrong relative abundance). In this way, it would be much easier to spot issues in the tree, which is one of the main applications of TAMPA within the CAMI benchmark. This

would be even more beneficial when there are large trees (example Figure S3), to quickly identify which part of the tree is the most problematic.

Response: We thank the reviewer for the great ideas. We have created a new mode for TAMPA called “contrast”, for better visualization of the comparison of the profile output with the ground truth, with a color spectrum. We have updated figure 2,3 with plots using the contrast mode, as well as added new text and images to the results and supplementary section (highlighted in red in the updated manuscript).

Reviewer 1 Comment 3:
In the main text there are references to Figure 1b and 1c, but the figures are labeled 2 and 3 instead.

Response: We thank the reviewer for pointing this out. We have changed the references to figure 2 and 3.

Reviewer 1 Comment 4:
The paper present a tool, as such I would expect a more detailed description of the tool implementation and options in the methods. This would be helpful for people to use the tool.

Response: We thank the reviewer for their comment. We have created a new subsection in the Methods section, called Implementation and Features (highlighted in red in the updated manuscript), outlining how the tool has been implemented, and the various options available to the user.

Reviewer 2 Comment 1:
It would be interesting to see (if possible) the comparison of three (or more) taxonomic profiles at the same time. The evaluations shown are always binary, but in a real-case scenario where a user would like to evaluate 3 or 4 different taxonomic profiling tools on his community, it would be great to be able to do it.

Response: We thank the reviewer for their comment. We have modified the tool so that 3 tools can be compared at once. Visualization of 4 and more tools is out of scope of this paper, we recommend that users perform multiple comparisons using TAMPA for improved visibility. We have added new figures and text to the results section (highlighted in red in the updated manuscript).

Reviewer 2 Comment 2:
Other than the evaluation on the agreement between two (or more) taxonomic profiling tools, it is not clear how TAMPA can drive improvement over biologically-relevant question. Although it is clear, as the authors stated in the introduction, that different taxonomic profilers (with different parameters settings) can produce very different taxonomic representations, to support this statement it will be important to be able to show, at least one case, where TAMPA can suggest a different taxonomic interpretation of a microbial community that is also biologically relevant.

Response: We thank the reviewer for this insightful comment. We have included a new subsection in our Results section containing an example where results from TAMPA is able to provide additional biologically relevant insights compared to standard metrics of a microbial community that are biologically relevant. In particular, we applied the two best-performing taxonomic profilers MetaPhyler 2.2 and mOTUs2.5.1 to the marine dataset published in the “Critical Assessment of Metagenome Interpretation: the second round of challenges” (https://doi.org/10.1038/s41592-022-01431-4). On comparing the output of MetaPhlAn with the gold standard, we use TAMPA to demonstrate that while MetaPhlAn was one of the top-performing profilers, it was not able to detect several clades, including Planctomycetes (Figure 4a). Additionally, while MetaPhlAn and mOTUs were both the top-performing profilers in terms of single-valued metrics such as F1-score and L1 norm error, there were several critical differences between their outputs, highlighted by TAMPA at the phylum level (Figure 4 b). For example, mOTUs failed to detect the Tenericutes group, while MetaPhlAn failed to detect Planctomycetes. There was also a considerable difference in the percentage abundance of other groups, such as Firmicutes.Note that this data set is simulated, though the corresponding ground truth was not revealed to the taxonomic profilers. Using simulated data is required in this setting since we need to know for sure what the “correct” answer is. If we used real biological data, further analyses and followup studies would be required to validate the hypothesis generated by TAMPA,

which is beyond the scope of this manuscript. We have added text to the discussion mentioning how TAMPA can be used for hypothesis generation, since confirming biological discoveries certainly requires more analyses than the visualizations returned by TAMPA.

Reviewer 2 Comment 3:
Figures in general appear to be of low-quality and stretched, please consider improving them as they are the main point of TAMPA.

Response: We thank the reviewer for this insight. We have replaced the figures in the manuscript with high-quality figures. We have also attached the high-quality figures separately in our submission for your reference.

Close
