# Supplementary material for: TAMPA: interpretable analysis and visualization of metagenomics-based taxon abundance profiles
Source: Gigascience. 2023 Feb 28;12:giad008. doi: 10.1093/gigascience/giad008 (PMC9972184; doi:10.1093/gigascience/giad008)
Supplement: giad008_Response_to_Reviewer_Comments_Revision_1 [file giad008_response_to_reviewer_comments_revision_1.pdf]

Author's Response To Reviewer Comments

Close

Editor comment 1: One remaining concern of our editorial board was that the options to distill the dataset down should be better documented in the Github repo, "with additional information to the README file in Github to document all of the options for the tool, with a focus on how to reduce the dataset for proper visualization."

Response: We thank the reviewers for their suggestion. We have updated the readme file in the Github repo.

Editor comment 2: A few minor formalities:

- please structure your abstract ("Background", "Findings" "Conclusions").

Response: We thank the reviewers for their suggestion. We have restructured the abstract as mentioned above.

- please format the section "Availability of supporting source code and requirements" as a table, like this example:

Project name: e.g. My bioinformatics project
Project home page: e.g. https://github.com/ISA-tools
Operating system(s): e.g. Platform independent
Programming language: e.g. Java
Other requirements: e.g. Java 1.3.1 or higher, Tomcat 4.0 or higher
License: e.g. GNU GPL, FreeBSD etc.
RRID: RRID: SCR_014986
biotools: XYZ

Response: We thank the reviewers for their suggestion. We have restructured the code availability section as mentioned above. We are still waiting for the RRID from biotools.

- please register any new software application in the bio.tools and SciCrunch.org databases to receive RRID (Research Resource Identification Initiative ID) and biotoolsID identifiers, and include these in your manuscript. Computational workflows should be registered in workflowhub.eu and the DOIs cited in the relevant places in the manuscript. These will facilitate tracking, reproducibility and re-use of your tool.

COMMENT 1:
In Figure 4b I can see that "Tenericutes" and "Planctomycetes" are both in orange, meaning that they both have been measured only by mOTUs. But in the main text I read "mOTUs failed to detect the Tenericutes group, while MetaPhlAn failed to detect Planctomycetes", which is wrong.
Response: We thank the reviewr for pointing this out. We have updated the manuscript to replace the statement with: “For example, MetaPhlAn failed to detect both the Tenericutes group, and Planctomycetes.‟

COMMENT 2:
I would improve the figure legends. In particular, the description of 4b is the same as in 2a and 3a and 1: "The size of the discs represents the total amount of relative abundance at the corresponding clade in the ground truth, or the tool prediction if that clade is not in the ground truth. If the tool predictions agree, a

disc is colored half orange and half teal. The proportion of teal to orange changes with respect to the disagreement in the prediction of that clade's relative abundance between the two tools being compared. Highlighted blue text represents clades where the difference between the relative abundances of the prediction and ground truth exceeds 30%". I would suggest to have this description only for figure 1, and then have a shorter description for the following figures.
Response: We thank the reviewer for their comment. We have updated the figure legends accordingly.

COMMENT 3:
The second color is described sometimes as "green" and sometimes as "teal". For clarity, I would suggest using just one of the two.
Response: We thank the reviewer for their comment. We have replaced all occurrences of “teal” with green.

Close
