# Supplementary material for: TAMPA: interpretable analysis and visualization of metagenomics-based taxon abundance profiles
Source: Gigascience. 2023 Feb 28;12:giad008. doi: 10.1093/gigascience/giad008 (PMC9972184; doi:10.1093/gigascience/giad008)
Supplement: giad008_Reviewer_1_Report_Original_Submission — Alessio Milanese -- 5/26/2022 Reviewed [file giad008_reviewer_1_report_original_submission.pdf]

Reviewer Report

Title: TAMPA: interpretable analysis and visualization of metagenomics-based taxon abundance profiles

Version: Original Submission **Date:** 5/26/2022

Reviewer name: Alessio Milanese

Reviewer Comments to Author:

Sarwal et al. present a tool to visualise and compare taxonomic profiles produced by differentprofiling methods. They provide a command line tool to produce trees to visualise taxonomicprofiles.Study strength:- The tool is useful to compare two taxonomic profiles, and there is no method available at themoment to do this.- The study is well embedded in the CAMI community benchmark.- The manuscript is generally well written.Major issues:- The tool should be easy to install, I would suggest to add it to bioconda, which allowthe installation of all dependencies (see CODE EVALUATION below).- TAMPA has limited applicability when comparing to Ground Truth.The tool allow to compare two different profiles, but there are conceptually two differentevaluations: 1) compare two different profiles, and 2) compare a profile to theGround Truth. The current implementation works good for the first evaluation, but Ican see it can be improved for the second. For example, it would be interesting tovisualise as a red circle false positive taxa (for example in Figure 2 Cyanobacteria),in blue false negatives (Nitrospirae in Figure 2) and in a gradient of white to greenwhen the taxa expected from the Ground truth is measured: in white if the relative abundanceis the same and in green if the relative abundance is different between expected andmeasured (with the colour intensity proportional to the error). In this way all correct taxawill be white, and looking at the tree colours we could understand the issue: red (falsepositive), blue (false negative), green (measure the correct taxa, but with wrong relativeabundance). In this way it would be much easier to spot issues in the tree,which is one of the main application of TAMPA within the CAMI benchmark. This would be evenmore beneficial when there are large trees (example Figure S3), to quickly identify whichpart of the tree is the most problematic.Minor issues:- In the main text there are references to Figure 1b and 1c, but the figures arelabelled 2 and 3 instead.- The paper present a tool, as such I would expect a more detailed description of the toolimplementation and options in the methods. This would be helpful for people to use the tool.CODE EVALUATION --I downloaded with conda (`conda install -c vsarwal tampa`) and have the following error:$ profile_to_plotTraceback (most recent call last):File "/miniconda3/bin/profile_to_plot", line 33, in <module>sys.exit(load_entry_point('CAMIViz==0.1.0', 'console_scripts', 'profile_to_plot')())File "/miniconda3/bin/profile_to_plot", line 25, in importlib_load_entry_pointreturn next(matches).load()File "/miniconda3/lib/python3.7/site-packages/importlib_metadata/__init__.py", line 167, in loadmodule = import_module(match.group('module'))File "/miniconda3/lib/python3.7/importlib/__init__.py", line 127, in import_modulereturn _bootstrap._gcd_import(name[level:], package, level)File "<frozen importlib._bootstrap>", line 1006, in _gcd_importFile "<frozen importlib._bootstrap>", line 983, in _find_and_loadFile "<frozen

importlib._bootstrap>", line 967, in _find_and_load_unlockedFile "<frozen importlib._bootstrap>", line 677, in _load_unlockedFile "<frozen importlib._bootstrap_external>", line 728, in exec_moduleFile "<frozen importlib._bootstrap>", line 219, in _call_with_frames_removedFile "/miniconda3/lib/python3.7/site-packages/src/profile_to_plot.py", line 2, in <module>from ProfilesLayout import ProfilesLayoutModuleNotFoundError: No module named 'ProfilesLayout'I have the same error if I follow the installation in https://github.com/dkoslicki/TAMPA.In order to make the tool easier to use I would suggest to:- change the name of the executable from `profile_to_plot` to `tampa`- you could add the package to bioconda to have all the dependencies installed- change the version to 1.0.0 for the release---

Level of Interest

Please indicate how interesting you found the manuscript: Choose an item.

Quality of Written English

Please indicate the quality of language in the manuscript: Choose an item.

Declaration of Competing Interests

Please complete a declaration of competing interests, considering the following questions:

- Have you in the past five years received reimbursements, fees, funding, or salary from an organisation that may in any way gain or lose financially from the publication of this manuscript, either now or in the future?
- Do you hold any stocks or shares in an organisation that may in any way gain or lose financially from the publication of this manuscript, either now or in the future?
- Do you hold or are you currently applying for any patents relating to the content of the manuscript?
- Have you received reimbursements, fees, funding, or salary from an organization that holds or has applied for patents relating to the content of the manuscript?
- Do you have any other financial competing interests?
- Do you have any non-financial competing interests in relation to this paper?

If you can answer no to all of the above, write 'I declare that I have no competing interests' below. If your reply is yes to any, please give details below.

I declare that I have no competing interests

I agree to the open peer review policy of the journal. I understand that my name will be included on my report to the authors and, if the manuscript is accepted for publication, my named report including any attachments I upload will be posted on the website along with the authors' responses. I agree for my

report to be made available under an Open Access Creative Commons CC-BY license (http://creativecommons.org/licenses/by/4.0/). I understand that any comments which I do not wish to be included in my named report can be included as confidential comments to the editors, which will not be published.

Choose an item.

To further support our reviewers, we have joined with Publons, where you can gain additional credit to further highlight your hard work (see: https://publons.com/journal/530/gigascience). On publication of this paper, your review will be automatically added to Publons, you can then choose whether or not to claim your Publons credit. I understand this statement.

Yes Choose an item.
