# Supplementary material for: TAMPA: interpretable analysis and visualization of metagenomics-based taxon abundance profiles
Source: Gigascience. 2023 Feb 28;12:giad008. doi: 10.1093/gigascience/giad008 (PMC9972184; doi:10.1093/gigascience/giad008)
Supplement: giad008_Reviewer_1_Report_Revision_1 — Alessio Milanese -- 11/22/2022 Reviewed [file giad008_reviewer_1_report_revision_1.pdf]

Reviewer Report

Title: TAMPA: interpretable analysis and visualization of metagenomics-based taxon abundance profiles

Version: Revision 1 **Date:** 11/22/2022

Reviewer name: Alessio Milanese

Reviewer Comments to Author:

Many thanks to the authors for their detailed responses to my comments.The edits have improved the manuscript and I have only few minor comments.COMMENT 1:In Figure 4b I can see that "Tenericutes" and "Planctomycetes" are both in orange, meaning that they bothhave been measured only by mOTUs. But in the main text I read "mOTUs failed to detect theTenericutes group, while MetaPhlAn failed to detect Planctomycetes", which is wrong.COMMENT 2:I would improve the figure legends. In particular, the description of 4b is the same as in 2a and 3a and 1:"The size of the discs represents the total amount of relative abundance at the corresponding clade in theground truth, or the tool prediction if that clade is not in the ground truth. If the tool predictions agree,a disc is colored half orange and half teal. The proportion of teal to orange changes with respect to thedisagreement in the prediction of that clade's relative abundance between the two tools beingcompared. Highlighted blue text represents clades where the difference between the relativeabundances of the prediction and ground truth exceeds 30%".I would suggest to have this description only for figure 1, and then have a shorter description for thefollowing figures.COMMENT 3:The second color is described sometimes as "green" and sometimes as "teal". For clarity, I would suggestusing just one of the two.

Level of Interest

Please indicate how interesting you found the manuscript: Choose an item.

Quality of Written English

Please indicate the quality of language in the manuscript: Choose an item.

Declaration of Competing Interests

Please complete a declaration of competing interests, considering the following questions:

- Have you in the past five years received reimbursements, fees, funding, or salary from an organisation that may in any way gain or lose financially from the publication of this manuscript, either now or in the future?
- Do you hold any stocks or shares in an organisation that may in any way gain or lose financially from the publication of this manuscript, either now or in the future?

- Do you hold or are you currently applying for any patents relating to the content of the manuscript?
- Have you received reimbursements, fees, funding, or salary from an organization that holds or has applied for patents relating to the content of the manuscript?
- Do you have any other financial competing interests?
- Do you have any non-financial competing interests in relation to this paper?

If you can answer no to all of the above, write 'I declare that I have no competing interests' below. If your reply is yes to any, please give details below.

I declare that I have no competing interests

I agree to the open peer review policy of the journal. I understand that my name will be included on my report to the authors and, if the manuscript is accepted for publication, my named report including any attachments I upload will be posted on the website along with the authors' responses. I agree for my report to be made available under an Open Access Creative Commons CC-BY license (http://creativecommons.org/licenses/by/4.0/). I understand that any comments which I do not wish to be included in my named report can be included as confidential comments to the editors, which will not be published.

Choose an item.

To further support our reviewers, we have joined with Publons, where you can gain additional credit to further highlight your hard work (see: https://publons.com/journal/530/gigascience). On publication of this paper, your review will be automatically added to Publons, you can then choose whether or not to claim your Publons credit. I understand this statement.

Yes Choose an item.
