# Supplementary material for: TAMPA: interpretable analysis and visualization of metagenomics-based taxon abundance profiles
Source: Gigascience. 2023 Feb 28;12:giad008. doi: 10.1093/gigascience/giad008 (PMC9972184; doi:10.1093/gigascience/giad008)
Supplement: giad008_Reviewer_2_Report_Original_Submission — Francesco Asnicar, Ph.D. -- 6/7/2022 Reviewed [file giad008_reviewer_2_report_original_submission.pdf]

Reviewer Report

Title: TAMPA: interpretable analysis and visualization of metagenomics-based taxon abundance profiles

Version: Original Submission **Date:** 6/7/2022

Reviewer name: Francesco Asnicar, Ph.D.

Reviewer Comments to Author:

The manuscript by Sarwal et al. presents a novel tool for a standardized visualization of metagenomic taxonomic profiler tools, named TAMPA, that also enables a more general assessments of performances of taxonomic profiler tools by providing an extensive of different metrics.It would be interesting to see (if possible) the comparison of three (or more) taxonomic profiles at the same time. The evaluations shown are always binary, but in a real-case scenario where a user would like to evaluate 3 or 4 different taxonomic profiling tools on his community, it would be great to be able to do it.Other than the evaluation on the agreement between two (or more) taxonomic profiling tools, it is not clear how TAMPA can drive improvement over biologically-relevant question. Although it is clear, as the authors stated in the introduction, that different taxonomic profilers (with different parameters settings) can produce very different taxonomic representations, to support this statement it will be important to be able to show, at least one case, where TAMPA can suggest a different taxonomic interpretation of a microbial community that is also biologically relevant.Figures in general appear to be of low-quality and stretched, please consider improving them as they are the main point of TAMPA.

Level of Interest

Please indicate how interesting you found the manuscript: Choose an item.

Quality of Written English

Please indicate the quality of language in the manuscript: Choose an item.

Declaration of Competing Interests

Please complete a declaration of competing interests, considering the following questions:

- Have you in the past five years received reimbursements, fees, funding, or salary from an organisation that may in any way gain or lose financially from the publication of this manuscript, either now or in the future?
- Do you hold any stocks or shares in an organisation that may in any way gain or lose financially from the publication of this manuscript, either now or in the future?
- Do you hold or are you currently applying for any patents relating to the content of the manuscript?

- Have you received reimbursements, fees, funding, or salary from an organization that holds or has applied for patents relating to the content of the manuscript?
- Do you have any other financial competing interests?
- Do you have any non-financial competing interests in relation to this paper?

If you can answer no to all of the above, write 'I declare that I have no competing interests' below. If your reply is yes to any, please give details below.

I declare that I have no competing interests

I agree to the open peer review policy of the journal. I understand that my name will be included on my report to the authors and, if the manuscript is accepted for publication, my named report including any attachments I upload will be posted on the website along with the authors' responses. I agree for my report to be made available under an Open Access Creative Commons CC-BY license (http://creativecommons.org/licenses/by/4.0/). I understand that any comments which I do not wish to be included in my named report can be included as confidential comments to the editors, which will not be published.

Choose an item.

To further support our reviewers, we have joined with Publons, where you can gain additional credit to further highlight your hard work (see: https://publons.com/journal/530/gigascience). On publication of this paper, your review will be automatically added to Publons, you can then choose whether or not to claim your Publons credit. I understand this statement.

Yes Choose an item.
